# Bovine Teat Cistern Microbiota Composition and Richness Are Associated With the Immune and Microbial Responses During Transition to Once-Daily Milking

**DOI:** 10.3389/fmicb.2020.602404

**Published:** 2020-12-16

**Authors:** Lucie Rault, Pierre-Alexandre Lévêque, Sarah Barbey, Frederic Launay, Hélène Larroque, Yves Le Loir, Pierre Germon, Jocelyne Guinard-Flament, Sergine Even

**Affiliations:** ^1^INRAE, Institut Agro, STLO, Rennes, France; ^2^INRAE, Institut Agro, PEGASE, Saint Gilles, France; ^3^INRAE, Domaine Expérimental du Pin, Gouffern En Auge, France; ^4^GenPhySE, Université de Toulouse, INRAE, ENVT, Castanet-Tolosan, France; ^5^INRAE, Université François Rabelais, ISP, Tours, France

**Keywords:** rare species, milking frequency, mastitis, inflammation, milk microbiota, teat microbiota

## Abstract

The relationship between microbiota and health has been widely reported in humans and animals. We established a link between teat cistern microbiota composition and bovine mastitis, an inflammatory disease often due to bacterial infections. To further decipher the relationships between teat cistern microbiota and immune and microbial responses, a switch from twice- to once-daily milking (ODM) in 31 initially healthy quarters of dairy cows was used to trigger an udder perturbation. In this study, a temporal relationship was reported between initial teat cistern microbiota composition and richness, the immune response to ODM, and mastitis development. Quarters with a low initial microbiota richness and taxonomic markers such as Bacteroidetes and Proteobacteria were associated with a higher rate of mastitis during ODM. Quarters with a higher richness and taxonomic markers such as Firmicutes, including the Lachnospiraceae family, and genera such as *Bifidobacterium* and *Corynebacterium* displayed early inflammation following transition to ODM but without developing mastitis (no infection). Short-term compositional shifts of microbiota indicates that microbiotas with a higher initial richness were more strongly altered by transition to ODM, with notably the disappearance of rare OTUs. Microbiota modifications were associated with an early innate immune system stimulation, which, in turn, may have contributed to the prevention of mastitis development.

## Introduction

The relationship between microbiota and health has been widely reported in both humans and animals (Kaakoush et al., 2012; Dunne et al., 2014; Oikonomou et al., 2014; Gaeta et al., 2016; Rautava, 2016; Bicalho et al., 2017). In bovine, microbiota associated with several anatomic sites, including the rumen, vaginal, and uterine tracts as well as the udder, probably contributes to animal health and performance (Santos and Bicalho, 2012; Jami et al., 2014; Oikonomou et al., 2014; Lima et al., 2015, 2017; Bicalho et al., 2017). Microbiota dysbiosis has notably been associated with inflammatory diseases such as mastitis (Oikonomou et al., 2014; Addis et al., 2016; Lima et al., 2017; Derakhshani et al., 2018a). Mastitis is an inflammation of the mammary gland, which generally results from bacterial infections of the mammary gland. It is characterized by an increase in the somatic cell count (SCC) in milk, with a neutrophil recruitment to the mammary gland as a result of chemoattractant interleukin secretion in milk (Riollet et al., 2000). Among them, Interleukine 8 (IL-8) secretion in milk is an early marker of the inflammatory response (Vangroenweghe et al., 2004; Herry et al., 2017). Mastitis is among the most common diseases in dairy cattle that cannot be fully controlled at this time. Development of new preventive and curative strategies may rely on a better management of environmental risk factors but also on a better understanding of interactions between the mammary gland and pathogens, taking all the factors that interfere with disease development and the ability of the immune system to counteract pathogens into account. One important and so far neglected factor is the mammary gland-associated microbiota. It should be investigated with regard to its role in disease prevention, which may help to identify new prevention strategies against mastitis.

We previously established a relationship between bovine teat cistern microbiota composition and the history of the corresponding quarter with regard to mastitis (Falentin et al., 2016) (see Figure 4 for anatomic definitions). This microbiota is the first one encountered by a pathogen entering the teat and, like a sentinel, it constitutes the first potential microbiological barrier against pathogen entry. In this study, it was not demonstrated whether dysbiosis was a result of infection or a factor favoring the onset of infection. Such causal relationships have been poorly explored so far. A study performed on bovine colostrum microbiota established that it differed in primiparous cows that developed mastitis in the first month of lactation, suggesting that microbiota can, to some extent, exert a positive or negative effect on mastitis development, depending on its composition (Lima et al., 2017). Whether this effect relies on a direct barrier effect with regard to pathogens or on the impact on host immune response has not yet been established.

In the present study, we further explored the relationship between the microbiota associated with the bovine teat cistern and the innate immune and microbial responses of the corresponding quarter by using transition from twice-daily milking (TDM) to once-daily milking (ODM). Reducing milking frequency is not a common practice (Stelwagen et al., 2013). It notably results in lower milk yield (Rémond and Pomiès, 2007). Nevertheless, it offers several advantages including lower labor requirements and farm working expenses and it has been used in extensive dairy production systems (Clark et al., 2006). ODM may be used throughout the lactation or during specific periods, in early lactation to alleviate the negative energy balance of cows, or during periods of pasture deficit or to manage cessation of milking at dry-off (Kay et al., 2013; Stelwagen et al., 2013). ODM was also shown to improve reproductive performance and animal health and welfare, notably heat stress and lameness (Rémond and Pomiès, 2005; Stelwagen et al., 2013). In the present study, this transition was used to trigger a perturbation of the mammary gland ecosystem. Changes in milking frequency can indeed be seen as a perturbation of mammary gland homeostasis. ODM results in milk accumulation and stasis in the mammary gland for 24 h, and has been associated with, at least transiently, udder inflammation associated or not with an infection (Stelwagen et al., 2013; Charton et al., 2018). An increase in SCC was indeed previously reported during transition from TDM to ODM (Rémond and Pomiès, 2007; Stelwagen et al., 2013; Charton et al., 2018). In our study, quarters initially free of inflammation and infection (i.e., no mastitis) were classified based on both their inflammatory response (SCC and Interleukin-8 in milk) and the development of infection following transition to ODM. Our results showed a temporal relationship between the initial composition and richness of teat cistern microbiota and the immune and microbial responses to transition to ODM.

## Materials and Methods

### Experimental Design

This study consisted of a specific assay on a subset of 34 cows used in an experiment on ODM and performed at the INRAE Pin-Au-Haras experimental farm located in Normandy (doi: 10.15454/1.5483257052131956E12, France) and approved by national legislation on animal care (French Ministry of Agriculture certification no. 01810.02). All experiments were performed in accordance with relevant guidelines and regulations. Crossbred Prim’Holstein x Normande cows were kept indoors in deep litter housing with daily mulching and fed as previously described (Charton et al., 2018). Cows were milked twice daily at 06h45 and at 16h45 and, after the switch to ODM at D0, they were milked once daily at 7h45 for 3 weeks. Classical hygienic procedures included cleaning of teats with individual paper towels before milking and post-milking teat dipping in iodine solution. Sampling was performed on three groups of animals between December 2013 and April 2014, i.e., 84+/−10 days after calving. Only quarters without any sign of inflammation or infection at the first day of ODM (D0) were retained for microbiota analysis in order to address the effect of ODM on healthy quarters only. Both inflammatory and microbiological status were determined as follows: approximately 30 mL of the cisternal milk of individual quarters were collected for the determination of SCC, IL-8 concentration and microbiological analysis. SCC was determined using a Fossomatic cell counter (Fos, Hillerød, Denmark) (Lilano, Saint-Lô, France). IL-8 concentration in cisternal milk was determined by ELISA as previously described (Roussel et al., 2015). Quarters were considered to be inflamed when the SCC value was higher than 100,000 cells/mL and or when IL-8 was detected in milk. The microbiological status was determined by plating 100 μL of cisternal milk on Columbia II containing 5% sheep blood (BD, Le Pont de Claix, France) and aerobically incubating it for 24 h–48 h at 37°C. Samples were considered to be infected (I) if more than five colonies with the same morphology were observed on plates. Pathogen identification was achieved by PCR amplification of the 16S rRNA gene directly on one representative colony, as previously described (Bouchard et al., 2015), and sequencing of the PCR product was done by LGC Genomics (Berlin, Germany). Quarters that were both infected and inflamed were considered as developing a mastitis.

Ninety-three quarters of the thirty-four cows included in the study were healthy at D0, i.e., free of inflammation and infection. Forty of these healthy quarters were initially retained for microbiota analysis (one or two quarters per cow). Finally, teat cistern microbiota analysis was successful for 31 healthy (non-inflamed and non-infected) quarters corresponding to 29 animals (see “Results” section) (Supplementary Table S1). These 31 analyzed quarters are indicated by “s,” followed by a number (e.g., s1, s2) and presented in Supplementary Table S1.

### Sample Collection

The sampling procedure was performed at D0, D3, and D14 during the morning milking, essentially as previously described (Bouchard et al., 2015). Briefly, teats were thoroughly washed with water and cleaned with 70% ethanol and individual paper towels. Foremilk samples, corresponding to the milk stored in the teat cistern, were collected in sterile plastic tubes stored on ice until processing in the laboratory for microbiota analysis. Cisternal milk was then collected for SCC and IL-8 determination and microbiological analysis, as described in the experimental design section.

Foremilk samples (3 mL) were mixed with 1/3 V of sodium citrate (1M, pH 7.5) and centrifuged (20 min, 4°C, 18,000 g). The pellet was washed in 1 ml of sodium citrate (20 g/L, pH 7.5), centrifuged (15 min, 4°C, 18,000 g) and stored at −20°C until DNA extraction.

### DNA Extraction, PCR Amplification of the V3-4 Region of Bacterial 16S rRNA Genes and Amplicon Sequencing

DNA extraction was performed as previously described (Falentin et al., 2016). PCR amplification of the V3-4 region of 16S rRNA genes was done using the composite forward and reverse primers 5′-CTTTCCCTACACGACGCTCTTCCGATCT*CCTACGGGNG GCWGCAG*-3′ and 5′-GGAGTTCAGACGTGTGCTCTTCCGA TCT*GACTACHVGGGTATCTAATCC*-3′, respectively, where the italicized sequences are the universal primers S-D-Bact-0341-b-S-17 and S-D-Bact-0785-a-A-21 (Klindworth et al., 2013), and which included primers for the Illumina sequencing platform. The PCR amplification of 16S rRNA was performed using a Veriti 96-well thermal cycler (Applied Biosystems, Foster City, CA, United States) in a 50-μL final volume containing 0.5 μM primers, 5 μL DNA sample and 1x NEBNext High Fidelity PCR Mastermix (New England Biolabs, Evry, France). The PCR conditions were as follows: denaturation step at 95°C for 5 min, followed by 12 cycles of denaturation at 98°C for 10 s, annealing at 61°C for 30 s, and extension at 72°C for 30 s and 18 cycles of denaturation at 98°C for 30 s, and extension at 72°C for 30 s. A final extension step was performed for 5 min at 72°C. Blank controls in which no DNA was added to the reaction were performed. Amplicon quality was checked on 1% agarose gel in 0.5X TBE. No amplicons were visible with blank control. Amplicons were purified after migration on 1.75% agarose gel (Lonza GTG agarose, Verviers, Belgium) in 0.5x TBE buffer, using the Qiaquick Gel Extraction Kit (Qiagen). Purified amplicons were further processed by the Genotoul GetPlage platform (Toulouse, France). After standardization to the same concentration, they were pooled for sequencing on the Illumina MiSeq platform (Illumina Inc., San Diego, CA, United States) according to specific barcode primers, using the MiSeq Reagent Kit.

### Sequence Library Analysis

Data quality control and analyses were performed using the Galaxy-supported FROGS pipeline hosted on the INRAE GENOTOUL bioinformatics platform (Escudié et al., 2018). The FROGS pre-process tool was first used to remove sequences in which the two primers were not present and to trim the primers. The tool also filtered reads with regard to amplicon size using an amplicon size between 380 and 500 bp and removed all sequences containing an ambiguous base. A general overview of pre-processed sequences allowed checking the general quality of the sequencing. The FROGS clustering step was performed with Swarm (Mahé et al., 2014), with an aggregation distance of 3 and a denoising clustering step. The chimera removal tool uses VSEARCH, combined with original cross-sample validation. This tool allows first to detect chimeras independently in each sample and validates a sequence as a chimera only if it is flagged as a chimera in all samples where it is present. An additional filter tool of FROGS was used to apply an abundance filter before the taxonomic affiliation process and to keep OTU with a minimum proportion of 0.00005. Affiliation was performed with Blastn+ using the SILVA 16S database.

### Statistical Analysis

Further data analysis was performed using R software (R development Core Team, 2013) and the phyloseq, vegan, and DESeq2 R packages (McMurdie and Holmes, 2013; Love et al., 2014) hosted on the INRAE MIGALE bioinformatics platform, using the biom file generated by the FROGS affiliation tool and a phylogenetic tree that was built using the align_seqs tool followed by the make_phylogeny tool of the QIIME pipeline (Caporaso et al., 2010). Analysis of alpha and beta diversity was performed after rarefaction with the rarefy_even_depth function of phyloseq.

Alpha-diversity was determined using the estimate_richness function of the plot_richness function of phyloseq through the estimation of several widely used diversity indices, the observed richness, Chao1, Shannon, Simpson, and inverse Simpson indices. Representation of alpha-diversity was achieved with the plot_richness function of phyloseq. Statistical analyses was performed on alpha-diversity indices with regard to the different clusters (see results), groups or days, as well as the animal groups and DNA extraction serial numbers, by using a one-way ANOVA, followed by Tukey’s Multiple Comparison Test and considering a *P*-value of less than 0.05. Spearman’s correlation analyses were performed between alpha-diversity indices at D0 and IL-8 concentration or SCC at D3, with a *P*-value of less than 0.05.

Beta diversity was estimated through the measurement of the Jaccard, Bray-Curtis, UniFrac and wUniFrac, distances, followed by Multi-Dimensional Scaling (MDS). The two former distances are compositional and take the OTU count into account. The latter two are phylogenetic distances, the weighted UniFrac (wUniFrac) distance also taking into account the abundances. Only results obtained with the UniFrac distance, allowing the best separation of samples with regard to groups, are presented. Hierarchical clustering of samples to merge closest communities was performed based on UniFrac distance using the ward linkage function.

The impact of the several factors including the “microbial cluster,” “group,” “day,” “animal group,” “DNA extraction serial number,” “IL-8 concentration,” and “SCC,” on microbiota composition was assessed by permutational multivariate ANOVA (PERMANOVA), with a *P*-value of 0.05 using the Adonis function of the Vegan R package. Impact on variance was also assessed by permutational multivariate analysis of dispersions (PERMDISP) using the betadisper function of the Vegan package. Combining PERMANOVA and PERMDISP allowed discrimination between true changes in bacterial composition and changes in variance.

Analysis of beta diversity made it possible to separate samples at D0 into two main clusters, C1 and C2. Discriminant analysis was performed between these clusters or between sampling days using the linear discriminant analysis (LDA) effect size (LEfSe) pipeline, hosted on the INRAE galaxy GENOTOUL bioinformatics platform (Segata et al., 2011). Briefly, the first step of the LEfSe method tested whether abundance in the different clusters are differentially distributed, using a Kruskal-Wallis rank sum test. An LDA model was then built to estimate the effect size of each differentially abundant taxon, i.e., to rank the differentially abundant taxa according to their relative difference among clusters. This step resulted in a list of taxonomic units that are discriminative with respect to the clusters and ranked, using a score, according to the effect size with which they differentiate clusters. This analysis was supplemented using the DESeq2 R package to determine the list of OTUs whose abundance was significantly different between the two clusters (Love et al., 2014).

### Data Availability

DNA sequence datasets are available at the Sequence Read Archive of the National Center for Biotechnology Information under the accession number PRJNA596102.

## Results

### Quarters Displayed Differential Inflammatory and Infection Status in Response to Transition to ODM

This study on bovine teat cistern microbiota was performed on a subset of 34 cows used in an experiment on ODM (unpublished data). Ninety-three quarters of these thirty-four cows were initially healthy, i.e., free of inflammation and infection at day 0 (D0), just before transition to ODM. These ninety-three quarters were distributed into five groups based on their inflammatory and infection status at day 3 (D3) and day 14 (D14) after transition to ODM. Group G1 comprises thirteen quarters (13%) that developed mastitis at D3 and or D14, characterized by both inflammation and infection. The four other groups comprise quarters that did not show any infection during the ODM period but that nevertheless exhibited different immune responses. Group G2 contains 22 quarters (24%) that did not show any inflammation during the ODM experiment based on SCC and IL-8 concentrations. The eleven quarters of group G3 (12%) displayed inflammation at D14, as revealed by IL-8 secretion in milk and/or an increase in SCC. Finally, Groups G4 and G5 contained 25 (27%) and 22 quarters (24%), respectively, that exhibited early inflammation at D3, as revealed mainly by IL-8 secretion in milk, which persisted at D14 for G5. These results showed that quarters did not homogeneously react to the transition to ODM with contrasted inflammatory responses. With about two third of the 93 initially non-inflamed and non-infected quarters displaying early inflammation at day 3 (D3) (within G1, G4, and G5 groups), the inflammatory response was likely related to the transition to ODM rather than any other external event, although we cannot totally exclude it. This is also in agreement with previous studies reporting at least a transient udder inflammation following transition to ODM (Stelwagen et al., 2013; Charton et al., 2018).

### Sequencing Results

Forty out of the ninety-three non-inflamed and non-infected quarters at day 0 (D0) were initially retained for microbiota analysis, just prior to transition to ODM. Only one or two quarters per cow were retained to prevent overrepresentation of some animals, as the number of healthy quarters at D0 varied from one to four quarters per cow. Amplification of the 16S rRNA gene was successful at D0 only for 31 of these forty quarters, allowing teat cistern microbiota of these 31 quarters to be analyzed (see Supplementary Table S1 for a detailed presentation of the microbiological and immune status of these 31 quarters). These 31 quarters were further analyzed at D3 and D14. However, amplification of the 16S rRNA gene was successful at both D3 and D14 for 19 out of the 31 quarters selected for microbiota analysis at D0. One sample at D3 and five samples at D14 were then removed from analysis due to an insufficient number of reads (<10,000). Microbiota were thus analyzed at day 3 (D3) and day 14 (D14) only on a limited number of quarters, i.e., 18 and 14 quarters at D3 and D14, respectively. Sequencing led to a total of 2 214 665 reads that were processed and filtered with the FROGS pipeline (Escudié et al., 2018), resulting in a total of 1,479,007 reads that were used for analysis. This corresponded to an average of 23 476 reads per sample. Rarefaction curves were shown to flatten for each sample, indicating that sequencing was deep enough to estimate the microbiota composition (see Supplementary Figure S1).

Sequencing of the negative control (PCR without genomic DNA) resulted in 412 reads, which dropped down to 36 reads after pre-processing in FROGS (see Supplementary Figure S2 for the taxonomic profile given by these 36 reads). Negative control was not further analyzed.

### Groups of Quarters Eliciting Different Inflammatory and Microbial Responses to the Transition to ODM Are Distributed Into Two Main Clusters With Different Compositions of Their Teat Cistern Microbiota at D0

Teat cistern microbiota was analyzed just prior to transition to ODM, i.e., at D0, on quarters that were all free of inflammation and infection at that time. An overview of microbiota indicates that dominant genera included *Staphylococcus* (average abundance of 8.5%), *Corynebacterium* (7.1%), *Flavobacterium* (5.5%), *Acinetobacter* (3.1%), *Aerococcus* (2.5%), *Candidatus Rhodoluna* (3.2%), *Bifidobacterium* (1.9%), *Enterococcus* (1.2%), *Rhodanobacter* (1.4%), the hgcl Clade (1.5%), *Arthrobacter* (0.8%), *Streptococcus* (0.7%), *Bacteroides* (0.7%), *Kurthia* (0.7%), *Chryseobacterium* (0.7%), *Pseudomonas* (0.6%) as well as members of the *Lachnospiraceae* (2.3%), *Ruminococcaceae* (4.3%), and *Comamonadaceae* (2.1%) families (see Supplementary Figure S2).

As a control, in order to check for biases related to the study design, we evaluated whether microbiota composition was related to the animal group (animals were split into three groups for the assay; see section “Materials and Methods”). No significant effect of this factor was revealed using permutational multivariate ANOVA (*p* > 0.5), indicating that modifications of parameters such as ambient temperature, diet, bedding between the three groups of animals did not significantly affect microbiota composition. Similarly, no significant effect was observed on alpha-diversity. Likewise the DNA extraction serial number did not significantly affect the microbiota composition and diversity, indicating that, although we could not exclude the presence of contaminating sequences related to DNA extraction (“kitome”) in our dataset, this contamination could not account for differences in microbiota composition.

**FIGURE 1:**
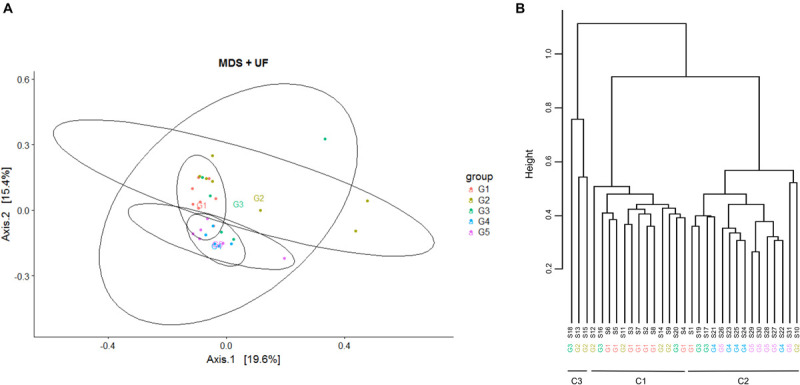
Multi-Dimensional Scaling (MDS) and hierarchical clustering on bovine teat cistern microbiota at day 0. **(A)** MDS was performed based on the measurement of the UniFrac distance. Samples are indicated by points and colored with regard to groups G1 to G5. Centroid positions are indicated for each group. **(B)** Hierarchical clustering of samples based on the UniFrac distance using the ward linkage function. Samples are distributed into two main clusters, C1 and C2, containing 13 and 15 quarters, respectively. Cluster C1 mainly includes quarters of groups G1 and G2, and cluster C2 mainly includes quarters of groups G4 and G5. An additional cluster (C3) includes only three samples, two from group G2 and one from G3. Quarters of group G3 are distributed among the three clusters.

In order to explore whether there was a relationship between microbiota composition and the response to transition to ODM, comparison of teat cistern microbiota composition between samples was further achieved by performing a Multi-Dimensional Scaling (MDS) as well as a hierarchical clustering with respect to groups as indicators of the inflammatory and microbial responses (Figure 1). Both MSD and hierarchical clustering revealed a separation, yet not perfect, between groups G1-G2 and G4-G5 and a strong dispersion of G3 quarter distribution. All G1 quarters but one that developed mastitis during ODM, and most of the G2 quarters that did not show any inflammation during the ODM experiment clustered together into cluster C1 (13 samples). All G4 and G5 quarters that exhibited early inflammation at D3 but without infection, clustered into cluster C2 along with one G2, one G1 and two G3 quarters (15 samples). Finally, an additional cluster, C3, contained only three samples: two G2 and one G3 quarter. Additional representation of samples using MSD with regard to those clusters is proposed on Supplementary Figure S3. This clustering suggested a complex structuration of the teat cistern microbiota population based on both the inflammatory and microbial responses. In particular, the initial teat cistern microbiota composition at D0 was not related to the inflammatory response to the transition to ODM solely, as quarters eliciting inflammation at D3 and or D14 were included in both clusters and C1 cluster included quarters eliciting inflammation (G1) or not (G2). This was further confirmed by permutational multivariate ANOVA as no significant effect of IL-8 concentration or SCC at D3 and D14 was observed on microbiota composition.

### Alpha-Diversity Analysis of Teat Cistern Microbiota at D0 Reveals a Higher Richness in C2 Quarters

**FIGURE 2:**
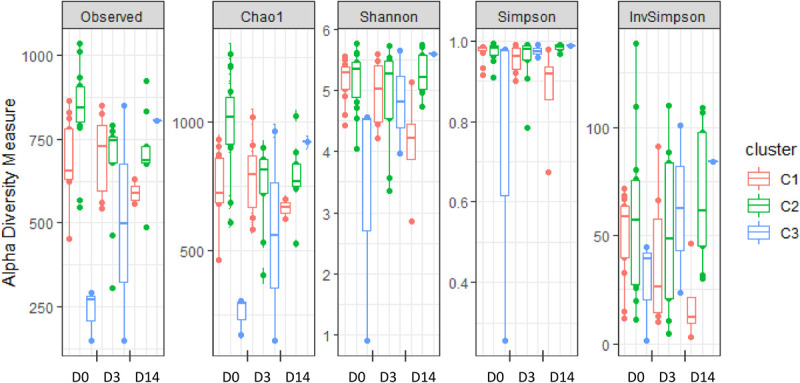
Alpha-diversity of teat cistern microbiota at day 0, day 3, and day 14 with regard to clusters, as illustrated by the distribution of several diversity indices: observed richness, Chao1, Shannon, Simpson, and Inverse Simpson indices.

Alpha-diversity of microbiota was estimated at D0 through the measurement of several indices: the observed richness, Chao1, Shannon, Simpson, and Inverse Simpson indices (Figure 2 and Supplementary Table S2). While the first two are indicators of species richness, the three other indicators measure both richness (the total number of OTUs) and evenness (the relative abundance of OTUs). Significant differences were observed between clusters for the observed richness, Chao1, Shannon and Simpson indices. In particular, the Chao1 index was significantly lower in C1 compared to C2, and a similar but not significant trend was observed for the observed richness. Moreover, the alpha-diversity of C3 quarters was significantly lower than that of C1 and C2 quarters, as revealed for all the indices except for the inverse Simpson. Altogether, these results indicate higher richness in C2 quarters, corresponding to those eliciting early inflammation at D3 without infection, compared to C1 quarters, corresponding to those that either developed mastitis or did not display any inflammation during ODM.

Significant differences were also observed when considering the “group” factor for the observed richness as well as Chao1. In particular, both indexes were significantly lower in quarters from G1 and G2 groups compared to quarters from G4 and G5. On the contrary, no significant differences were observed between groups for Shannon and Simpson indexes.

Relation between alpha-diversity and immune responses to transition to ODM was further explored through Spearman’s correlation analyses between alpha-diversity indices and IL-8 concentration or SCC at D3. A significant correlation was found between the observed richness, Chao1 and Shannon indices at D0 and IL-8 concentration at D3 and a similar trend was observed for the simpson index. A significant correlation was also found between the observed richness at D0 and SCC at D3, but not with the other indices. This result reveals a positive relation between the initial richness of teat cistern microbiota at D0 and the immune response following transition to ODM (at D3).

### Discriminant Analysis Revealed Differential Microbiota Composition Between C1 and C2 Quarters at D0

A general overview of the mean microbiota revealed several differences in the taxonomic composition of clusters C1 and C2 (Supplementary Figure S4), which was further confirmed by permutational multivariate ANOVA (PERMANOVA) using compositional distances (Jaccard, Bray-Curtis) as well as phylogenetic distances (UniFrac and wUniFrac) (*p* < 0.0001). Differences in the bacterial community composition were not associated to changes in variance between clusters C1 and C2, as revealed by permutational multivariate analysis of dispersions (PERMDISP), which confirmed the differential composition between C1 and C2 clusters. Of note, differences in the bacterial community composition was also revealed by PERMANOVA using these four distances when considering G1-G2 quarters vs. G4-G5 quarters (*p* < 0.01). However, this was also associated to changes in variance as revealed by PERMDISP for UniFrac and Jaccard distances, which did not allow to clearly confirm differences in bacterial community composition when considering groups of quarters (G1-G2 and G4-G5). This was probably related to the higher dispersion observed for G2 quarters (Figure 1).

**FIGURE 3:**
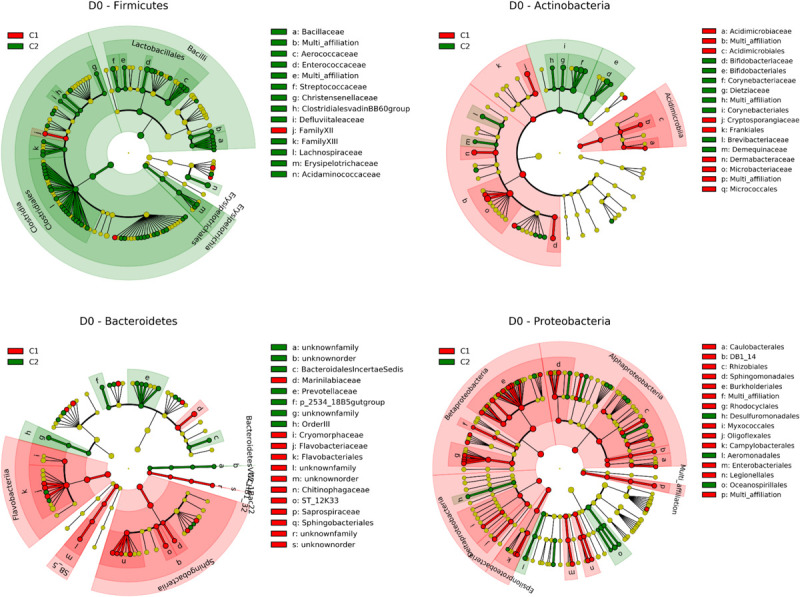
Taxonomic representation of differentially abundant taxa between C1 (contains mainly G1 and G2 quarters) and C2 (contains mainly G4 and G5 quarters) at day 0 for Firmicutes Actinobacteria Bacteroidetes and Proteobacteria as determined by the LEfSe pipeline. Differences are represented by the color of the cluster where the taxon is more abundant (red indicates C1 and green indicates C2). See Supplementary Table S3 for a complete list of discriminant taxa.

Data were thus subjected to discriminant analysis using the LEfSe pipeline in order to identify taxonomic units whose abundance was significantly different between C1 and C2 (Figure 3, Supplementary Table S3, and Supplementary Figure S5). Cluster C3, which contained only three quarters, was excluded from the analysis. Discrimination between clusters occurred at high taxonomic levels such as phylum. Bacteroidetes and Proteobacteria were more abundant in C1 than C2, with relative abundances of 20.2% and 13.8% in C1 and 13.5% and 11.7% in C2. This was notably related to a higher abundance of *Flavobacterium* and the Comamonadaceae family in C1. On the contrary, Firmicutes were more abundant in C2 than C1 (42.9 and 25.2%, respectively). This was related to a higher abundance of the Lachnospiraceae and Aerococcaceae families, including genera such as *Coprococcus, Blautia* and *Roseburia*, and *Aerococcus*. Among Actinobacteria, *Bifidobacterium* and *Corynebacterium*, were more abundant in C2, whereas *Candidatus Rhodoluna* were more abundant in C1. This discriminant analysis performed at different taxonomic levels was supplemented by the determination of the complete list of OTUs whose abundance was different between the two clusters (Supplementary Table S4). This analysis revealed that 442 OTUs were differentially abundant between the two clusters, 338 OTUs being more abundant in C2 than C1. Half of them (226 OTUs) belonged to the Firmicutes and corresponded to several genera including *Aerococcus* but also *Solibacillus*, *Staphylococcus, Facklamia, Enterococcus*, and *Streptococcus*, although these genera were not discriminant per se between C1 and C2. Fifty-one OTUs belonging to the Lachnospiraceae family, including several *Coprococcus* OTUs, and seventy OTUs belonging to the Ruminococcaceae family, including several *Ruminococcus* OTUs were also more abundant in C2. In addition, among OTUs whose abundance was higher in cluster C2, 54 OTUs belonged to the Actinobacteria, with several OTUs corresponding to the *Bifidobacterium, Corynebacterium*, and *Nocardiodes* genera, and 86 OTUs belonged to the Bacteroidetes including several *Bacteroides* OTUs. Endly, 45 OTUs corresponded to the Proteobacteria, including few *Acinetobacter* OTUs.

### Dynamics of Teat Cistern Microbiota in the Two Main Clusters of Samples in Response to ODM

Teat cistern microbiota composition and diversity were further explored in 18 quarters at D3 (seven C1, nine C2, and two C3 quarters) and 14 quarters at D14 (four C1, nine C2, and one C3 quarter) to evaluate the impact of transition to ODM on teat cistern microbiota. Due to the limited number of quarters in C3, analysis was only performed for C1 and C2. No significant differences of alpha-diversity indices occurred over time for C1, whereas a significant decrease in the Chao 1 index occurred at D3 compared to D0 for C2 (Figure 2). A similar but not significant trend was observed for the observed richness (*p* = 0.14). Likewise, the Chao 1 index tended to be lower at D14 compared to D0 for C2 (*p* = 0.07). No significant differences were observed between days for C2 for the other indices.

Comparison of microbiota revealed a day effect for the C2 cluster (*p* = 0.035) (Supplementary Figure S4). A similar but not significant trend was observed for C1 (*p* = 0.057). However, when considering only the short-term effects of transition to ODM, microbiota compositions were significantly different between D0 and D3 only for C2 (*p* = 0.025), but not for C1. Microbiota composition still tended to differ between C1 and C2 at D3 (*p* = 0.10), but not at D14. This may be due to the small number of C1 quarters at D14 that precludes statistical significance.

Considering the significant changes in microbiota composition between D0 and D3 only for the C2 cluster, a discriminant analysis was performed (Supplementary Figures S6, S5 and Supplementary Table S5). ODM resulted in a lower abundance of Actinobacteria (from 21 to 14.9%) compared to D0 in C2 quarters, including *Bifidobacterium* and *Nocardioides*. Bacteroidetes and Betaproteobacteria abundances increased at D3. On the contrary, within Firmicutes, all discriminant taxa but two were more abundant at D0 compared to D3.

## Discussion

Mammary gland inflammation has been mainly considered for a long time as the result of an infection by a pathogen. From the viewpoint of Koch’s postulates, inflammatory response may be driven by various factors such as host genetics, as well as pathogen factors such as virulence factors (Burvenich et al., 2003; Le Marechal et al., 2011; Vayssier-Taussat et al., 2014; Martin et al., 2018). Nevertheless, increasing evidence in several ecosystems supports a relationship between microbiota associated with a given anatomic site and the inflammatory status of this site, unbalanced microbiota possibly being associated with immune disorders (Schwiertz et al., 2010; Kaakoush et al., 2012; Oikonomou et al., 2014). This relationship is generally highlighted through correlation at the time of disorder. In this study, we used transition to ODM as a perturbation of the mammary gland system, starting from a healthy status (no infection, no inflammation), as a way to reveal (or not) the relationship between teat cistern microbiota and immune and microbial responses to the perturbation.

### Relationship Between Bovine Teat Cistern Microbiota Richness and Composition, and Immune and Microbial Responses to ODM

Bovine teat cistern microbiota of healthy quarters at D0 were distributed into two main clusters that are, to some extent, in agreement with groups that exhibit differential immune and microbial responses to ODM. Cluster C1 contained mainly G1 and G2 quarters while cluster C2 contained mainly G4 and G5 quarters. Differences in alpha and beta diversity between Clusters C1 and C2 were indeed confirmed when considering G1-G2 vs. G4-G5 groups. G3 quarters were distributed in all clusters, suggesting that G3 was not relevant to characterize response to ODM. The G3 group corresponded to quarters that displayed inflammation 14 days following transition to ODM. This inflammation may result either from transition to ODM or to another external event. We cannot exclude that some of these G3 quarters developed a mastitis at D14, although we did not identified any pathogen, or that it corresponded to a physical trauma of the udder.

**FIGURE 4:**
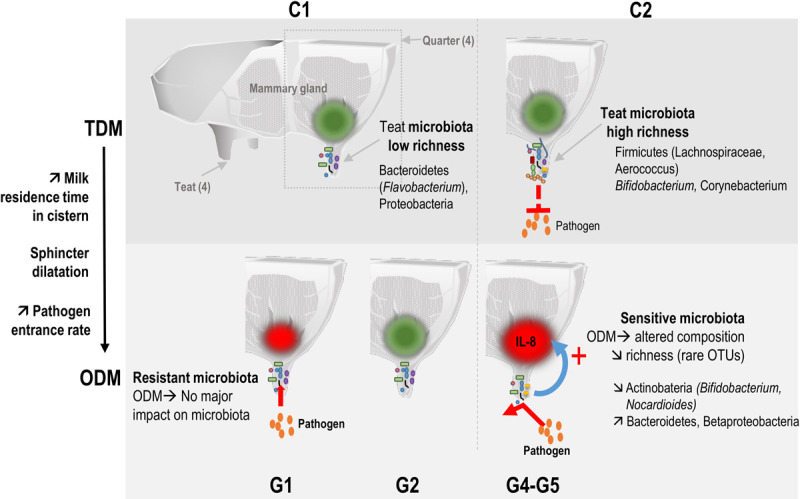
Temporal relationship between bovine teat cistern microbiota composition and richness and the immune and microbial responses during transition to once-daily milking. Microbiota of quarters were distributed into two main clusters. Cluster C1 included microbiota with low initial richness (day 0) exerting a low colonization resistance to pathogens. C1 microbiota were hardly affected by ODM-induced perturbation (resistant microbiota), which may have resulted in a lack of immune system stimulation (green spot). The higher rate of pathogen entrance through the teat canal thus resulted in a higher rate of mastitis cases in this cluster. Cluster C2 included microbiota with higher initial richness at day 0 that may have contributed to a higher colonization resistance. These C2 microbiota were more strongly altered in response to the transition to ODM (sensitive microbiota). Changes in microbial composition and the decrease in richness may have contributed to the stimulation of the innate immune system (red spot). This might have contributed to combatting pathogens entering the mammary gland, thus preventing the development of infection.

Our results, through the use of an induced perturbation of the mammary gland homeostasis, experimentally corroborate the general observation of a positive correlation between richness and protection against infections. A positive correlation between microbiota alpha-diversity and health was previously reported in the bovine mammary gland context at the time of infection (Braem et al., 2012) or at a distance, either before (Lima et al., 2017) or after the infection (Falentin et al., 2016). Higher richness can prevent the invasion of new species through “colonization resistance” by increasing the range of antagonistic activities and competition for nutrients, as observed in soil microbial communities (Stecher et al., 2013; Vivant et al., 2013; Mallon et al., 2015; Jousset et al., 2017). ODM probably favors colonization of teat skin by pathogens due to the lower frequency of cleaning. Moreover, ODM, due to increased intramammary pressure and sphincter dilatation, results in milk leakage and, consequently, favors the entrance of pathogens (Gleeson et al., 2007; Stelwagen et al., 2013). This higher rate of pathogen entrance, combined with the lower colonization resistance in relation to the lower richness in C1 quarters (G1-G2), may account for mastitis development in some C1 quarters (corresponding to G1 quarters) (Figure 4). Conversely, the higher richness in C2 quarters (G4-G5) may have contributed to a higher colonization resistance, thus protecting against pathogen entrance and mastitis development (G4 and G5 quarters).

Short-term compositional shifts of microbiota at D3 following transition to ODM suggested a possible link between microbiota richness, microbiota sensitivity to ODM-induced perturbation and the ability of the host innate immune system to sense and to respond to these microbiota variations (Figure 4). This hypothesis is supported by the correlation observed between the initial teat microbiota richness (et D0) and IL-8 secretion in milk following transition to ODM (at D3). Transition to ODM through an increase in the milking interval changes the residence time of bacteria in the teat and leads to modifications of milk composition (Charton et al., 2018) that can affect the growth capacities and competition for nutrients between microbiota members. A more diverse microbiota (in C2 compared to C1 quarters) would be more sensitive to such changes in environmental conditions, at least because more OTUs are likely to be affected. In agreement with this hypothesis, both microbiota composition and richness were significantly affected during transition to ODM between D0 and D3 for C2 but not C1 clusters. Changes in microbiota composition of C2 quarters may have been sensed by the innate immune system, leading to inflammation (IL-8 production at D3). Conversely, the lesser impact of transition to ODM on microbiota of C1 quarters precluded innate immune system stimulation (as observed in G2 quarters). An early stimulation of the innate immune system, as observed in groups G4 and G5, may have contributed to combatting pathogens entering the mammary gland, thus preventing the infection from developing. Of note, when considering full ongoing lactation, none of the C2 quarters developed clinical mastitis, whereas four C1 quarters developed one mastitis following the experiment, including one G2 quarter, suggesting a higher susceptibility to mastitis for C1 compared to C2 quarters. Additional experiments on a larger number of animals will be necessary to clarify this possible relationship between microbiota richness, their sensitivity or resistance to perturbation and, consequently, sensitivity of the immune system to these perturbations. The use of additional markers of the innate immune response would also be of interest to complete this first analysis. This could include additional cytokines or the differential SCC, which gives information on the different immune cell ratio in milk (polymorphonuclear neutrophils and lymphocytes vs. macrophages) and was proposed as a marker of early and low-level inflammation (Schwarz et al., 2011; Herry et al., 2017; Wall et al., 2018).

Our results point out differences in alpha-diversity for indicators of species richness only, such as the Chao1 index, suggesting that differences in diversity were mainly related to rare OTUs. This difference in richness was also illustrated when looking at OTUs whose abundance was significantly different between C1 and C2: more than 99% (438 out of the 442) of the differentially abundant OTUs between C1 and C2 were less abundant in C1 compared to C2. Besides, ∼69% of these differentially abundant OTUs (306 OTUs) displayed a very low abundance (<0.0005 of total reads at D0), i.e., 100-fold lower than the most abundant OTUs, suggesting as well a role of low abundant OTUs. Rare OTUs or species are increasingly recognized as key contributors to ecosystem functioning, leading to the concept of keystone species (Jousset et al., 2017). Rare species can drive key processes in ecosystem functioning and stability and they represent a reservoir of genes, allowing adaptation to changing conditions and, as a result, resilience of the ecosystem function (Jousset et al., 2017). They can affect community assembly through metabolic interaction by supporting the growth of dominant species (Gotoh et al., 2019) and contribute to host health by preventing the invasion of new species through “colonization resistance” (Mallon et al., 2015). Likewise, by increasing the range of interactions with the host, rare species probably contribute to the interaction with the immune system (stimulation/inhibition) (Stecher et al., 2013; Jousset et al., 2017). In agreement with this last point, our results suggest a key role of rare species within the teat cistern microbiota through direct colonization resistance and/or interaction with the immune system of the mammary gland.

### Taxonomic Markers of Quarters Eliciting Different Immune Responses During Transition to ODM

Apart from differences in richness, taxonomic composition was significantly different between C1 and C2 microbiota or when comparing G1-G2 with G4-G5 quarters, suggesting a relationship between specific taxonomic markers and responses to transition to ODM. In particular, the discriminant analysis and the identification of differentially abundant OTUs between C1 and C2 revealed a higher abundance of *Bifidobacterium*, *Nocardiodes* and members of the Ruminococcaceae and Lachnospiraceae families including *Blautia, Coprococcus*, and *Roseburia*, in C2 (Supplementary Figure S5). These taxa were previously reported as taxonomic markers of healthy quarters that had never undergone mastitis (Falentin et al., 2016). *Corynebacterium* were also more abundant in C2. Several *Corynebacterium* species have been associated with moderate mastitis (Braem et al., 2012; Derakhshani et al., 2018a). However, contradictory conclusions have been drawn on *Corynebacterium*. While Hogan observed a higher rate of environmental mastitis in quarters infected by *Corynebacterium* (Hogan et al., 1988), it was also hypothesized that it may confer protection against mastitis thanks to its ability to inhibit major pathogens (Woodward et al., 1987). Interestingly, our results partially corroborate the positive correlations observed by Derakhshani et al. (2018b) between specific taxa and richness of teat canal, milk, and colostrum microbiota as the abundance of Lachnospiraceae, several members of the Ruminococcaceae family, and *Bifidobacterium* was higher in C2 quarters with the highest richness.

Focusing on the short-term effect of transition to ODM, the modifications of the C2 microbiota composition, combined with the lower richness observed at D3, bring the C2 microbiota at D3 closer to microbiota observed at D0 in C1 (Supplementary Figure S5). Thus, ODM appears to reduce the abundance of taxa associated with healthy quarters, including *Bifidobacterium*, members of the Clostridiales (Falentin et al., 2016) and *Nocardiodes* (Oikonomou et al., 2014). These modifications of microbiota composition and richness might account for the higher risk of intramammary infection during ODM that has sometimes been reported, depending on the assay and the ODM duration (Lacy-Hulbert et al., 2005; Rémond and Pomiès, 2005; Stelwagen et al., 2013).

Several questions remain on teat cistern microbiota compositional shifts in response to ODM. The impact of ODM on teat cistern microbiota was not fully explored at D14 due to the limited number of samples at D14, especially for C1. Nevertheless, it is interesting to note that both the microbiota composition and richness of C2 quarters were significantly different at D14 compared to D0, suggesting that, beyond a short-term effect of ODM on teat cistern microbiota at D3, this “continuous perturbation” resulted in longer-term effects. Whether microbiota composition at D14 was still fluctuating or reached a new stable state resulting from adaptation remains to be determined. Likewise, the possible resilience of teat cistern microbiota following return to TDM will require additional experiments.

In conclusion, this study shows for the first time a temporal relationship between initial teat cistern microbiota richness and composition, the immune response to ODM, and the risk of developing mastitis. Moreover, our results provide new insights by suggesting a relationship between microbiota richness, its sensitivity to perturbation with notably the disappearance of rare OTUs, and the sensitivity of the innate immune system. Finally, this study corroborates previous results (Falentin et al., 2016) by pointing out similar taxonomic markers of a healthy state such as *Bifidobacterium* or members of the Clostridia class. This study was done on a limited number of animals. Additional experiments at a larger scale will thus be necessary to confirm this temporal relationship between teat cistern microbiota and mammary gland immune response and health in response to a perturbation and explore a possible causal relationship. A functional exploration using shotgun metagenomics or transcriptomics may also help to identify the functional alterations related to the modifications of teat microbiota composition and give new insights into the mechanisms that may contribute to the different responses to transition to ODM and the potential protective effect of some healthy-related taxa. Nevertheless, these results encourage us to seek levers to increase teat cistern microbiota richness or to favor specific taxonomic markers that could be tested for their potential to prevent mastitis in dairy herds.

## Data Availability Statement

The datasets presented in this study can be found in online repositories. The names of the repository/repositories and accession number(s) can be found below: https://www.ncbi.nlm.nih.gov/, PRJNA596102.

## Ethics Statement

The animal study was reviewed and approved by national legislation on animal care (French Ministry of Agriculture certification no. 01810.02).

## Author Contributions

LR and P-AL collected samples, carried out the experiments, and analyzed the data. SB and FL coordinated the *in vivo* assay and sample collection. HL designed the *in vivo* assay. YL contributed to study design and helped writing the manuscript. PG contributed to study design, carried out immunoassays, and helped writing the manuscript. JG-F designed the study and helped writing the manuscript. SE designed the study, carried out bioinformatics analyses, and wrote the manuscript. All authors read and gave final approval for publication.

## Conflict of Interest

The authors declare that the research was conducted in the absence of any commercial or financial relationships that could be construed as a potential conflict of interest.

## Supplementary Material

Supplementary Figure 1 Rarefaction curves.

Supplementary Figure 2 Mean bovine teat taxonomic profiles combining different taxonomic levels at day 0. The 20 dominant genera (17) or families (3) are presented. Other genera are pooled in the corresponding phylum and referred to as “other phylum.” Firmicutes are displayed in blue, Actinobacteria in orange/brown, Bacteroidetes in green, and Proteobacteria in red. As a comparison, the taxonomic profile corresponding to the negative control (36 reads only) is also presented. These reads corresponded to 24 different OTUs, with one or two reads per OTU. Half of these reads were within the 100 most abundant OTUs associated to samples as well, suggesting a possible, yet very limited, contamination of the negative control by samples during PCR and sequencing steps.

Supplementary Figure 3 Multi-Dimensional Scaling (MDS) on bovine teat cistern microbiota at day 0. MDS was performed based on the measurement of the UniFrac distance. Samples are indicated by points and colored with regard to clusters obtained by hierarchical clustering (see Figure 1B). Centroid positions are indicated for each cluster.

Supplementary Figure 4 Bovine teat taxonomic profiles combining different taxonomic levels at day 0, day 3 and day 14, with regard to clusters C1 and C2 (see “Results” section). The 20 dominant genera (17) or families (3) are presented. Other genera are pooled in the corresponding phylum and referred to as “other phylum.” Each bar represents a specific cluster at a specific day of sampling. Firmicutes are displayed in blue, Actinobacteria in orange/brown, Bacteroidetes in green, and Proteobacteria in red.

Supplementary Figure 5 Abundance distribution of differentially abundant taxonomic units between Clusters C1 and C2 at D0, or between D0 and D3 for quarters assigned to Cluster C2. The mean abundance is indicated by a cross.

Supplementary Figure 6 Taxonomic representation of differentially abundant taxa between day 0 and day 3 for quarters assigned to Cluster C2, as determined by the LEfSe pipeline. Differences are represented by the color of the day where the taxon is more abundant (red indicates day 0 and green indicates day 3).

Supplementary Table 1 Sample description and associated metadata.

Supplementary Table 2 Alpha-diversity indices of teat microbiota for each sample at day 0.

Supplementary Table 3 Differentially abundant taxonomic units between Clusters C1 and C2 at D0 as determined by the LEfSe pipeline. Average abundances (expressed as percentages) are presented for each cluster. LefSe results include the LDA score and *p*-val. Most discriminant taxa (with a LDA score > 4.5) are in bold.

Supplementary Table 4 Differentially abundant OTUs between Clusters C1 and C2 at D0 as determined by the DESeq2 package.

Supplementary Table 5 Differentially abundant taxonomic units between D0 and D3 for quarters assigned to Cluster C2, as determined by the LEfSe pipeline. Average abundances (expressed as percentages) are presented for each day. LefSe results include the LDA score and *p*-value. Most discriminant taxa (with a LDA score > 4) are in bold.
