# Supplementary material for: Bovine Teat Cistern Microbiota Composition and Richness Are Associated With the Immune and Microbial Responses During Transition to Once-Daily Milking
Source: Front Microbiol. 2020 Dec 16;11:602404. doi: 10.3389/fmicb.2020.602404 (PMC7772349; doi:10.3389/fmicb.2020.602404)
Supplement: Supplementary Figure 1 — Rarefaction curves. [file Data_Sheet_1.PDF]

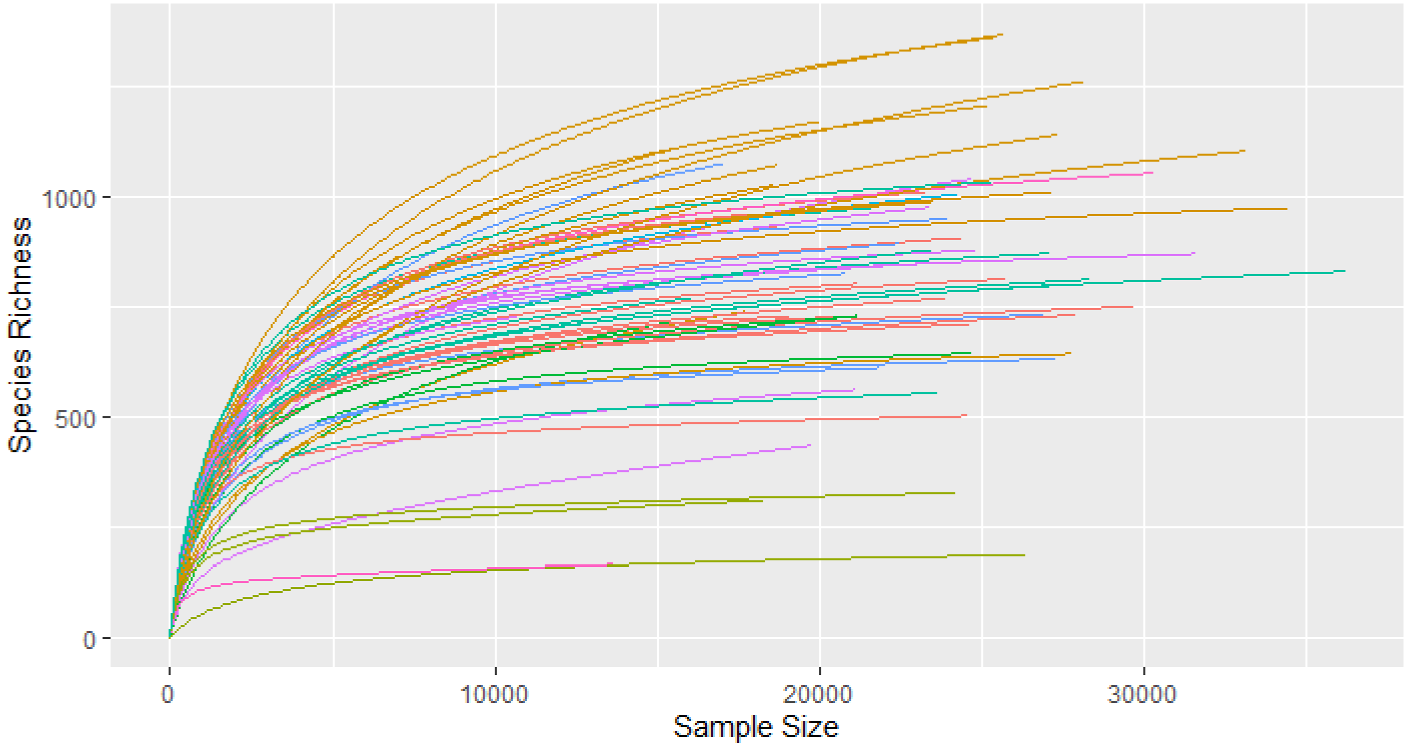

Figure S1: Rarefaction curves

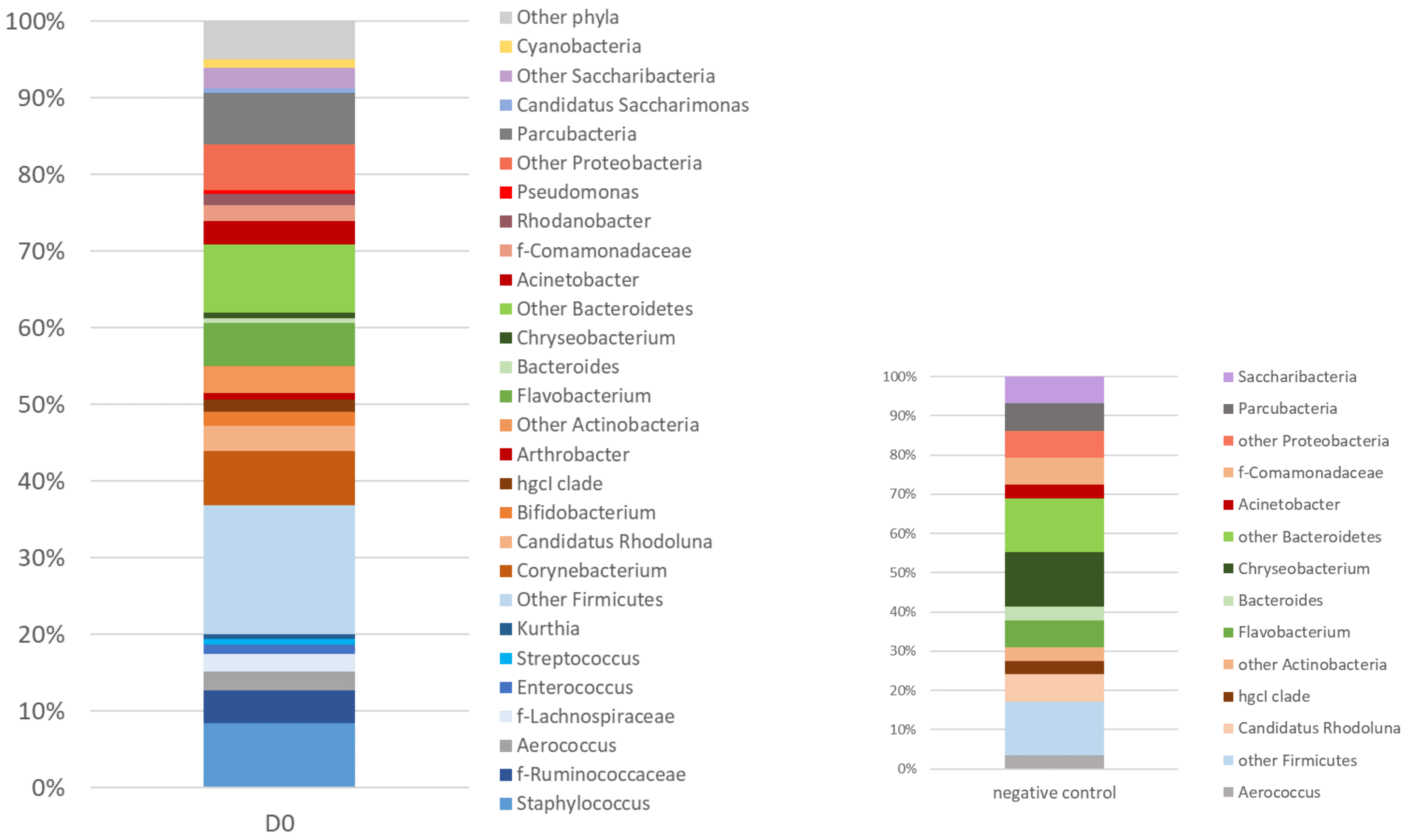

Figure S2. Mean bovine teat taxonomic profiles combining different taxonomic levels at day 0. The 20 dominant genera (17) or families (3) are presented. Other genera are pooled in the corresponding phylum and referred to as "other phylum". Firmicutes are displayed in blue, Actinobacteria in orange/brown, Bacteroidetes in green, and Proteobacteria in red.
As a comparison, the taxonomic profile corresponding to the negative control (36 reads only) is also presented. These reads corresponded to 24 different OTUs, with one or two reads per OTU. Half of these reads were within the 100 most abundant OTUs associated to samples as well, suggesting a possible, yet very limited, contamination of the negative control by samples during PCR and sequencing steps.

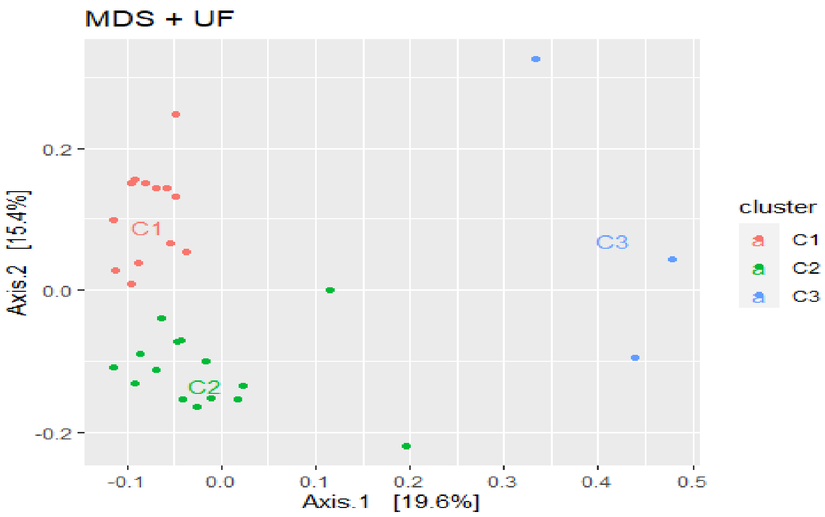

Figure S3. Multi-Dimensional Scaling (MDS) on bovine teat cistern microbiota at day 0. MDS was performed based on the measurement of the UniFrac distance. Samples are indicated by points and colored with regard to clusters obtained by hierarchical clustering (see Figure 1B). Centroid positions are indicated for each cluster.

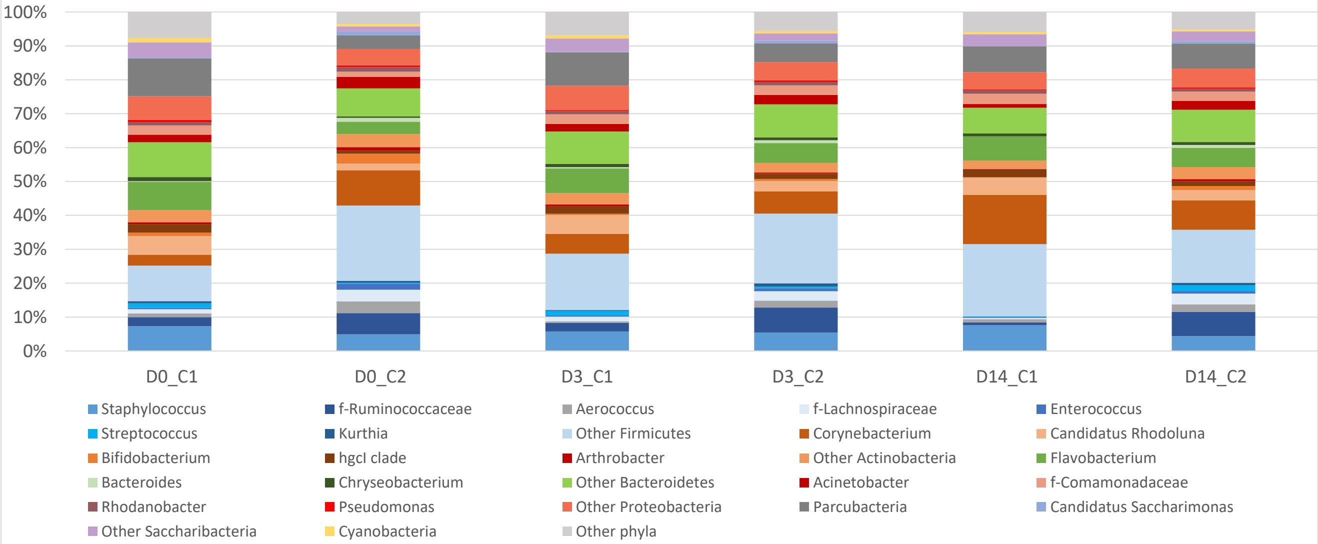

Figure S4: Bovine teat taxonomic profiles combining different taxonomic levels at day 0, day 3 and day 14, with regard to clusters C1 and C2 (see Results section). The 20 dominant genera (17) or families (3) are presented. Other genera are pooled in the c

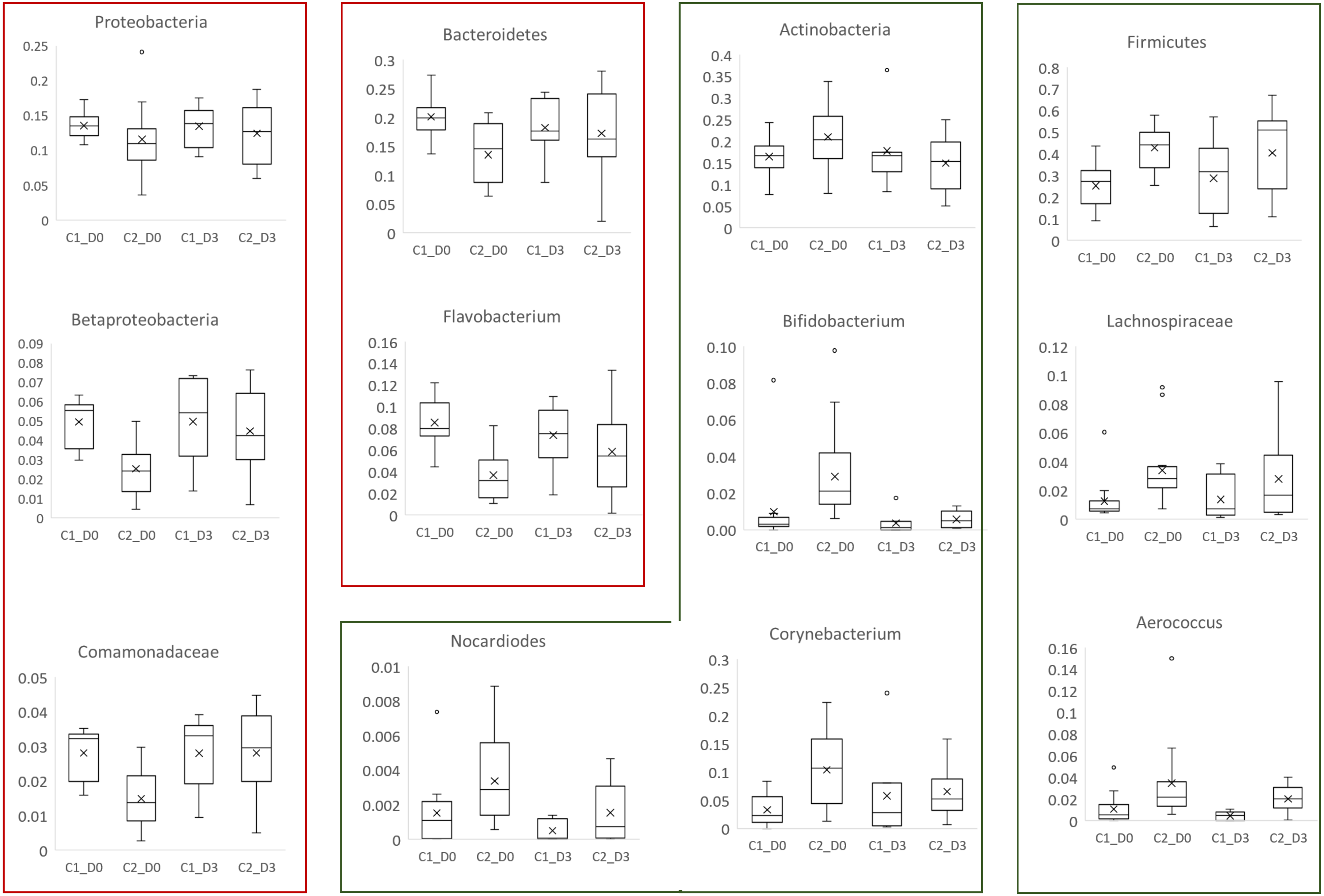

Figure S5. Abundance distribution of differentially abundant taxonomic units between Clusters C1 and C2 at D0, or between D0 and D3 for quarters assigned to Cluster C2. The mean abundance is indicated by a cross.

C2

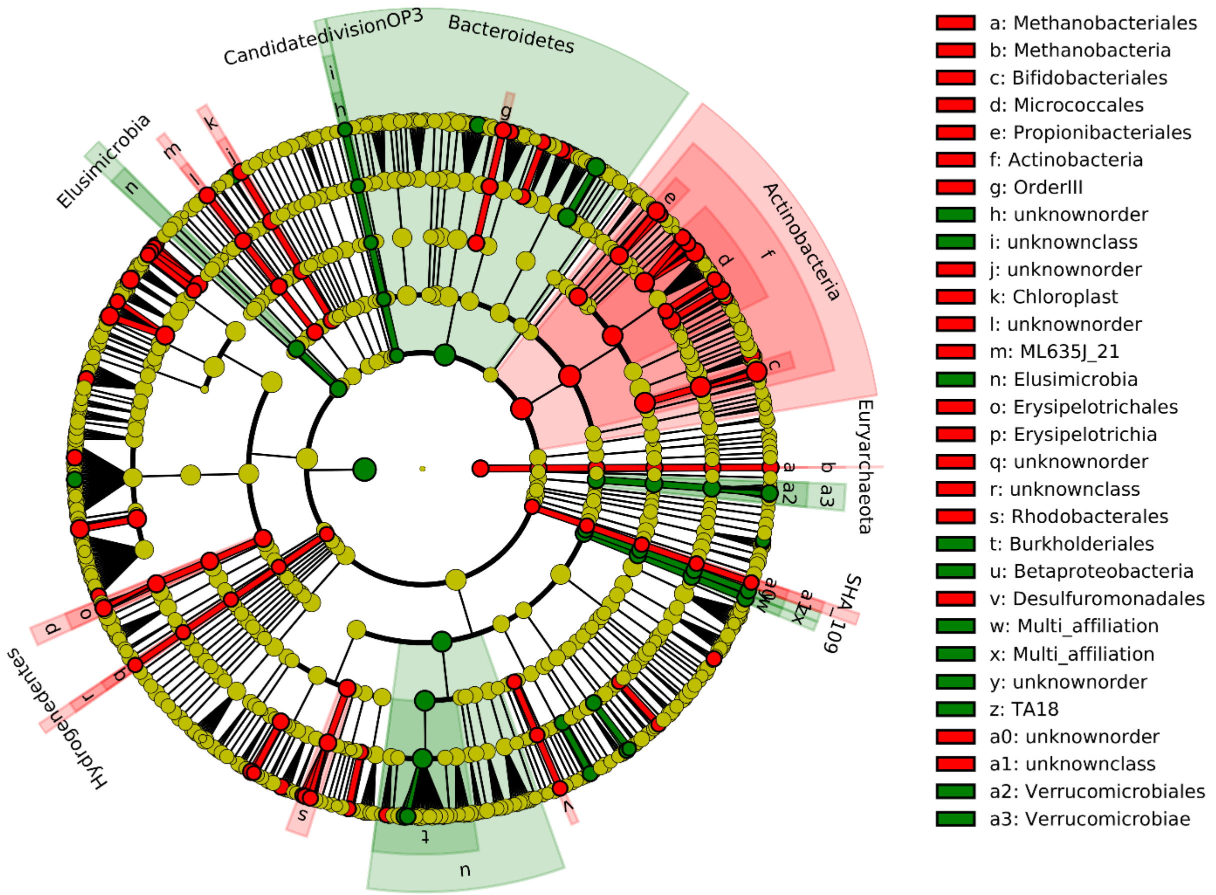

Figure S6: Taxonomic representation of differentially abundant taxa between day 0 and day 3 for quarters assigned to Cluster C2, as determined by the LEfSe pipeline. Differences are represented by the color of the day where the taxon is more abundant (red indicates day 0 and green indicates day 3).

Table S1. Sample description and associated metadata

quarter name (1)	group	cluster	lactation	Animal group (6)	DNA extraction Nb (7)	Day in milk	Day 0 (5)		Day 3			Day 14			Health status at sampling day (3)			health status of sampled quarter-ongoing lactation (4)		
							SCC cell/mL	IL8 pg/mL	microbiology	SCC cell/mL	IL8 pg/mL	microbiology	SCC cell/mL	IL8 pg/mL	D0	D3	D14	mastitis before transition to ODM	mastitis during transition to ODM	mastitis following period of ODM
s1	G1	C2	4	1	6	73	17 000	0		23 000	0	ND	4 274 000	106	NI-NInflam	NI-NInflam	ND-Inflam (2)		CM-D14	
s2	G1	C1	2	2	1	92	2 000	0	*Staphylococcus haemolyticus*	2 670 000	352	*Staphylococcus haemolyticus*	163 000	0	NI-NInflam	I-Inflam	I-Inflam		SCM-D3 and D14	
s3	G1	C1	3	3	7	89	30 000	0	*Corynebacterium camporealensis*	71 000	30		50 000	0	NI-NInflam	I-Inflam	NI-NInflam	CM	SCM-D3	
s4	G1	C1	2	2	4	107	6 000	0	*Staphylococcus pasteuri*	3 000	37		8 000	22	NI-NInflam	I-Inflam	NI-Inflam		SCM-D3 and D14	CM
s5	G1	C1	2	3	3	97	11 000	0		48 000	189	*Brevibacterium paucivorans*	9 000	33	NI-NInflam	NI-Inflam	I-Inflam		SCM-D14	
s6	G1	C1	2	1	3	71	10 000	0		17 000	23	*Aerococcus sp.*	3 815 000	497	NI-NInflam	NI-Inflam	I-Inflam		CM-D14	CM
s7	G1	C1	2	1	5	74	22 000	0		65 000	21	*Staphylococcus aureus*	11 000	0	NI-NInflam	NI-Inflam	I-NInflam		SCM-D14	CM
s8	G1	C1	2	1	7	82	23 000	0	*Staphylococcus devriesei*	82 000	45		38 000	0	NI-NInflam	I-Inflam	NI-NInflam		SCM-D3	
s9	G2	C1	2	1	4	77	21 000	0		18 000	0		22 000	0	NI-NInflam	NI-NInflam	NI-NInflam			CM
s10	G2	C2	2	2	5	84	3 000	0		6 000	0		3 000	0	NI-NInflam	NI-NInflam	NI-NInflam			
s11	G2	C1	2	3	1	89	11 000	0		13 000	0		10 000	0	NI-NInflam	NI-NInflam	NI-NInflam			
s12	G2	C1	3	3	6	85	3 000	0		3 000	0		3 000	0	NI-NInflam	NI-NInflam	NI-NInflam			
s13	G2	C3	2	2	8	78	6 000	0		5 000	0		3 000	0	NI-NInflam	NI-NInflam	NI-NInflam			
s14	G2	C1	2	1	2	86	7 000	0		71 000	0		9 000	0	NI-NInflam	NI-NInflam	NI-NInflam			
s15	G2	C3	3	3	9	85	3 000	0		3 000	0		2 000	0	NI-NInflam	NI-NInflam	NI-NInflam			
s16	G3	C1	2	1	4	84	43 000	0		25 000	0		126 000	58	NI-NInflam	NI-NInflam	NI-Inflam			
s17	G3	C2	2	2	6	98	8 000	0		5 000	0		131 000	37	NI-NInflam	NI-NInflam	NI-Inflam			
s18	G3	C3	2	3	5	63	6 000	0		24 000	0		8 000	22	NI-NInflam	NI-NInflam	NI-Inflam			
s19	G3	C2	4	1	7	81	14 000	0		7 000	0		45 000	39	NI-NInflam	NI-NInflam	NI-Inflam			
s20	G3	C1	2	3	2	88	21 000	0		21 000	0		36 000	40	NI-NInflam	NI-NInflam	NI-Inflam			
s21	G4	C2	2	2	8	101	6 000	0		16 000	65		16 000	0	NI-NInflam	NI-Inflam	NI-NInflam			
s22	G4	C2	2	2	1	99	19 000	0		33 000	39		21 000	0	NI-NInflam	NI-Inflam	NI-NInflam			
s23	G4	C2	2	3	8	90	4 000	0		67 000	78		11 000	0	NI-NInflam	NI-Inflam	NI-NInflam			
s24	G4	C2	2	3	8	77	5 000	0		25 000	35		5 000	0	NI-NInflam	NI-Inflam	NI-NInflam			
s25	G4	C2	2	3	9	82	4 000	0		23 000	23		11 000	0	NI-NInflam	NI-Inflam	NI-NInflam			
s26	G5	C2	2	3	1	87	16 000	0		15 000	56		49 000	46	NI-NInflam	NI-Inflam	NI-Inflam			
s27	G5	C2	2	3	4	85	17 000	0		47 000	148		9 000	75	NI-NInflam	NI-Inflam	NI-Inflam			
s28	G5	C2	3	2	2	93	17 000	0		21 000	27		7 000	20	NI-NInflam	NI-Inflam	NI-Inflam			
s29	G5	C2	2	3	3	65	9 000	0		64 000	80		93 000	172	NI-NInflam	NI-Inflam	NI-Inflam			
s30	G5	C2	3	3	5	82	15 000	0		42 000	41		19 000	30	NI-NInflam	NI-Inflam	NI-Inflam			
s31	G5	C2	2	3	9	65	6 000	0		26 000	41		24 000	34	NI-NInflam	NI-Inflam	NI-Inflam			

(1) all quarters correspond to different animals except s12 and s15 which correspond to the same animal and s29 and s31, which were also sampled on the same animal

(2) quarter treated with antibiotics before sampling for microbiology

(3) NI : non infected; I : Infected ; NInflam: no inflammation; Inflam: IL8>0 and or SCC>100000 cells/mL

(4) CM: clinical mastitis (recorded as such and treated with antibiotherapy); SCM: subclinical mastitis as determined by analysis at D3 and D14 (infection and inflammation, no antibitoic treatment)

(5) all quarters were free of infection at DO

(6) Sampling was performed on three groups of animals

(7) DNA extraction serial number

Table S2. Alpha diversity indices of teat microbiota for each sample

quarter	day	group	cluster	Observed	Chao1	Shannon	Simpson	InvSimpson
s1	D0	G1	C2	843	934	5.43	0.98	56.3
s2	D0	G1	C1	876	976	5.43	0.98	65.2
s3	D0	G1	C1	631	688	5.38	0.99	70.8
s4	D0	G1	C1	658	784	5.02	0.97	33.1
s5	D0	G1	C1	832	929	5.59	0.99	71.6
s6	D0	G1	C1	770	832	5.54	0.99	71.8
s7	D0	G1	C1	675	756	4.89	0.98	40.7
s8	D0	G1	C1	839	938	5.34	0.98	55.1
s9	D0	G2	C1	635	699	4.44	0.92	11.9
s10	D0	G2	C2	542	628	4.52	0.96	27.4
s11	D0	G2	C1	712	779	5.31	0.98	63.0
s12	D0	G2	C1	448	466	5.18	0.98	58.8
s13	D0	G2	C3	291	313	4.55	0.97	39.4
s14	D0	G2	C1	644	685	5.05	0.97	38.6
s15	D0	G2	C3	263	281	4.53	0.98	45.5
s16	D0	G3	C1	648	698	4.62	0.94	15.5
s17	D0	G3	C2	807	898	5.35	0.98	66.3
s18	D0	G3	C3	145	161	0.89	0.25	1.3
s19	D0	G3	C2	757	948	4.72	0.96	27.5
s20	D0	G3	C1	644	693	5.37	0.98	58.6
s21	D0	G4	C2	855	976	5.75	0.99	134.9
s22	D0	G4	C2	942	1079	5.48	0.99	78.4
s23	D0	G4	C2	787	1052	4.82	0.97	34.2
s24	D0	G4	C2	879	1127	5.15	0.98	56.8
s25	D0	G4	C2	875	1019	5.57	0.99	106.3
s26	D0	G5	C2	821	981	5.44	0.99	81.3
s27	D0	G5	C2	994	1238	4.93	0.95	20.3
s28	D0	G5	C2	926	1085	5.04	0.96	25.9
s29	D0	G5	C2	1045	1245	5.59	0.99	71.3
s30	D0	G5	C2	832	956	5.47	0.98	66.1
s31	D0	G5	C2	583	716	4.08	0.91	11.3
s1	D3	G1	C2	768	835	5.75	0.99	114.7
s2	D3	G1	C1	868	966	4.53	0.91	10.5
s3	D3	G1	C1	737	800	5.51	0.99	69.5
s4	D3	G1	C1	755	851	4.98	0.96	24.9
s5	D3	G1	C1	631	689	5.34	0.98	52.0
s9	D3	G2	C1	553	594	4.22	0.92	12.8
s10	D3	G2	C2	757	813	5.49	0.99	83.2
s11	D3	G2	C1	548	581	4.51	0.94	16.0
s13	D3	G2	C3	153	160	4.01	0.96	25.1
s16	D3	G3	C1	830	894	5.61	0.99	90.0
s17	D3	G3	C2	751	835	5.27	0.98	45.1
s18	D3	G3	C3	852	959	5.68	0.99	103.0
s21	D3	G4	C2	679	717	5.55	0.99	90.3
s22	D3	G4	C2	748	796	5.35	0.98	53.8
s23	D3	G4	C2	313	404	3.58	0.91	11.0
s26	D3	G5	C2	722	947	4.55	0.95	21.1

s27	D3	G5	C2	794	907	5.43	0.99	79.7
s28	D3	G5	C2	459	536	3.40	0.79	4.8
s1	D14	G1	C2	729	806	4.95	0.97	31.5
s2	D14	G1	C1	570	682	2.84	0.68	3.1
s4	D14	G1	C1	574	609	4.24	0.92	13.2
s9	D14	G2	C1	623	718	5.14	0.98	46.3
s10	D14	G2	C2	685	787	5.06	0.98	47.6
s11	D14	G2	C1	625	683	4.17	0.91	11.5
s17	D14	G3	C2	676	751	5.24	0.98	61.9
s18	D14	G3	C3	790	923	5.58	0.99	83.0
s21	D14	G4	C2	485	511	4.73	0.97	29.9
s22	D14	G4	C2	877	976	5.73	0.99	106.7
s23	D14	G4	C2	715	784	5.56	0.99	106.9
s26	D14	G5	C2	850	919	5.70	0.99	97.4
s27	D14	G5	C2	669	722	5.14	0.98	44.8
s28	D14	G5	C2	662	751	5.32	0.99	67.9

Table S3. Differentially abundant taxonomic units between Clusters C1 and C2 at D0 as determined by the LEfSe pipeline. Average abundances (expressed as percentages) are presented for each cluster. LefSe results include the LDA score and p-val. Most discriminant taxa (with a LDA score > 4.5) are in bold.

discriminant taxa									mean abundance	
phylum	class	order	family	genus	species	LDA score	more abun-dant in Cluster	pvalue	C1	C2
**Taxa more abundant in cluster C1**										
Acidobacteria						3.81	C1	0.0001	1.081%	0.453%
Acidobacteria	Acidobacteria					3.62	C1	0.0001	0.848%	0.381%
Acidobacteria	Acidobacteria	Subgroup4				3.32	C1	0.002	0.402%	0.166%
Acidobacteria	Acidobacteria	Subgroup4	11_24			2.71	C1	0.010	0.104%	0.024%
Acidobacteria	Acidobacteria	Subgroup4	11_24	unknown genus		2.71	C1	0.010	0.104%	0.024%
Acidobacteria	Acidobacteria	Subgroup6				2.83	C1	0.023	0.321%	0.164%
Acidobacteria	Acidobacteria	Subgroup6	unknown family			2.83	C1	0.023	0.321%	0.164%
Acidobacteria	Acidobacteria	Subgroup6	unknown family	unknown genus		2.83	C1	0.023	0.321%	0.164%
Acidobacteria	Holophagae					3.36	C1	0.008	0.233%	0.072%
Acidobacteria	Holophagae	Holophagales				3.36	C1	0.008	0.233%	0.072%
Acidobacteria	Holophagae	Holophagales	Holophagaceae			3.36	C1	0.008	0.233%	0.072%
Actinobacteria										
Actinobacteria	Acidimicrobiia					3.59	C1	0.003	0.227%	0.133%
Actinobacteria	Acidimicrobiia	Acidimicrobiales				3.59	C1	0.003	0.227%	0.133%
Actinobacteria	Acidimicrobiia	Acidimicrobiales	Acidimicrobiaceae			3.08	C1	0.007	0.037%	0.004%
Actinobacteria	Acidimicrobiia	Acidimicrobiales	Acidimicrobiaceae	CL500_29marinegroup		2.99	C1	0.026	0.029%	0.004%
Actinobacteria	Acidimicrobiia	Acidimicrobiales	Multi_affiliation			3.19	C1	0.001	0.048%	0.010%
Actinobacteria	Acidimicrobiia	Acidimicrobiales	Multi_affiliation	Multi_affiliation		3.19	C1	0.001	0.048%	0.010%
Actinobacteria	Actinobacteria	Actinomycetales	Actinomycetaceae	Actinomyces		3.01	C1	0.038	0.033%	0.002%
Actinobacteria	Actinobacteria	Frankiales				4.29	C1	0.001	3.107%	1.109%
Actinobacteria	Actinobacteria	Frankiales	Cryptosporangiaceae			4.22	C1	0.003	0.489%	0.205%
Actinobacteria	Actinobacteria	Frankiales	Cryptosporangiaceae	Fodinicola		4.22	C1	0.003	0.489%	0.205%
Actinobacteria	Actinobacteria	Frankiales	Geodermatophilaceae	unknown genus	Sporichthyasp_	2.75	C1	0.037	0.019%	0.017%
**Actinobacteria**	**Actinobacteria**	**Micrococcales**				**5.34**	**C1**	**0.00002**	**7.578%**	**4.616%**
Actinobacteria	Actinobacteria	Micrococcales	Dermabacteraceae			4.15	C1	0.012	0.621%	0.401%
Actinobacteria	Actinobacteria	Micrococcales	Dermabacteraceae	Brachybacterium		4.15	C1	0.012	0.621%	0.401%
**Actinobacteria**	**Actinobacteria**	**Micrococcales**	**Microbacteriaceae**			**5.27**	**C1**	**0.0001**	**5.947%**	**2.284%**
Actinobacteria	Actinobacteria	Micrococcales	Microbacteriaceae	CandidatusPlanktoluna		3.63	C1	0.002	0.127%	0.034%
**Actinobacteria**	**Actinobacteria**	**Micrococcales**	**Microbacteriaceae**	**CandidatusRhodoluna**		**5.24**	**C1**	**0.00005**	**5.442%**	**1.886%**
Actinobacteria	Actinobacteria	Micrococcales	Micrococcaceae	Arthrobacter		3.87	C1	0.002	0.538%	1.065%
Actinobacteria	Actinobacteria	Micrococcales	Multi_affiliation			3.85	C1	0.014	0.243%	0.118%
Actinobacteria	Actinobacteria	Micrococcales	Multi_affiliation	Multi_affiliation		3.85	C1	0.014	0.243%	0.118%
**Bacteroidetes**						**5.19**	**C1**	**0.005**	**20.200%**	**13.528%**
Bacteroidetes	Bacteroidia	Bacteroidales	Marinilabiaceae			4.15	C1	0.001	0.434%	0.140%
Bacteroidetes	Bacteroidia	Bacteroidales	Marinilabiaceae	Mangroviflexus		4.15	C1	0.001	0.434%	0.140%
Bacteroidetes	Bacteroidia	Bacteroidales	Porphyromonadaceae	Paludibacter		3.31	C1	0.012	0.221%	0.082%
Bacteroidetes	Bacteroidia	Bacteroidales	Rikenellaceae	Blvii28wastewater_sludgegroup		3.86	C1	0.036	0.265%	0.172%
Bacteroidetes	Cytophagia	Cytophagales	Cytophagaceae	Cytophaga		4.02	C1	0.009	0.419%	0.199%
Bacteroidetes	Cytophagia	Cytophagales	Cytophagaceae	Flectobacillus		3.33	C1	0.005	0.063%	0.022%
**Bacteroidetes**	**Flavobacteriia**					**4.79**	**C1**	**0.029**	**11.533%**	**5.861%**
**Bacteroidetes**	**Flavobacteriia**	**Flavobacteriales**				**4.79**	**C1**	**0.029**	**11.533%**	**5.861%**
Bacteroidetes	Flavobacteriia	Flavobacteriales	Cryomorphaceae			3.72	C1	0.045	0.427%	0.257%
**Bacteroidetes**	**Flavobacteriia**	**Flavobacteriales**	**Flavobacteriaceae**			**4.75**	**C1**	**0.032**	**11.027%**	**5.579%**
Bacteroidetes	Flavobacteriia	Flavobacteriales	Flavobacteriaceae	Chryseobacterium	Multi_affiliation	2.42	C1	0.023	1.208%	0.389%
Bacteroidetes	Flavobacteriia	Flavobacteriales	Flavobacteriaceae	Elizabethkingia		3.69	C1	0.0001	0.146%	0.033%
Bacteroidetes	Flavobacteriia	Flavobacteriales	Flavobacteriaceae	Flavobacterium		4.28	C1	0.001	8.525%	3.761%
Bacteroidetes	Flavobacteriia	Flavobacteriales	Flavobacteriaceae	Multi_affiliation		3.86	C1	0.0002	0.220%	0.053%
Bacteroidetes	SB_5					3.27	C1	0.016	0.051%	0.013%
Bacteroidetes	SB_5	unknown order				3.27	C1	0.016	0.051%	0.013%
Bacteroidetes	SB_5	unknown order	unknown family			3.27	C1	0.016	0.051%	0.013%
Bacteroidetes	SB_5	unknown order	unknown family	unknown genus		3.27	C1	0.016	0.051%	0.013%
**Bacteroidetes**	**Sphingobacteriia**					**4.59**	**C1**	**0.001**	**3.136%**	**1.839%**
**Bacteroidetes**	**Sphingobacteriia**	**Sphingobacteriales**				**4.59**	**C1**	**0.001**	**3.136%**	**1.839%**
Bacteroidetes	Sphingobacteriia	Sphingobacteriale	Chitinophagaceae			4.09	C1	0.001	0.879%	0.325%
Bacteroidetes	Sphingobacteriia	Sphingobacteriale	Chitinophagaceae	Chitinophaga		3.68	C1	0.003	0.145%	0.033%
Bacteroidetes	Sphingobacteriia	Sphingobacteriale	Chitinophagaceae	Ferruginibacter		3.00	C1	0.037	0.045%	0.006%
Bacteroidetes	Sphingobacteriia	Sphingobacteriale	Chitinophagaceae	Hydrotalea		2.79	C1	0.032	0.034%	0.000%
Bacteroidetes	Sphingobacteriia	Sphingobacteriale	Chitinophagaceae	Sediminibacterium		3.51	C1	0.012	0.233%	0.115%
Bacteroidetes	Sphingobacteriia	Sphingobacteriale	Saprospiraceae			3.17	C1	0.023	0.805%	0.361%
Bacteroidetes	Sphingobacteriia	Sphingobacteriale	Sphingobacteriaceae	Mucilaginibacter		3.43	C1	0.014	0.084%	0.015%
Bacteroidetes	Sphingobacteriia	Sphingobacteriale	Sphingobacteriaceae	Pedobacter		3.76	C1	0.001	0.378%	0.111%
Bacteroidetes	Sphingobacteriia	Sphingobacteriale	Sphingobacteriaceae	Sphingobacterium	bacteriumCC_YY411	2.70	C1	0.012	0.274%	0.576%
Bacteroidetes	Sphingobacteriia	Sphingobacteriale	ST_12K33			3.92	C1	0.004	0.254%	0.109%
Bacteroidetes	Sphingobacteriia	Sphingobacteriale	ST_12K33	unknown genus		3.92	C1	0.004	0.254%	0.109%
Bacteroidetes	WCHB1_32					3.04	C1	0.022	0.201%	0.064%
Bacteroidetes	WCHB1_32	unknown order				3.04	C1	0.022	0.201%	0.064%
Bacteroidetes	WCHB1_32	unknown order	unknown family			3.04	C1	0.022	0.201%	0.064%
Bacteroidetes	WCHB1_32	unknown order	unknown family	unknown genus		3.04	C1	0.022	0.201%	0.064%
CandidatedivisionOP3						2.73	C1	0.004	0.014%	0.003%
CandidatedivisionC	unknown class					2.73	C1	0.004	0.014%	0.003%
CandidatedivisionC	unknown class	unknown order				2.73	C1	0.004	0.014%	0.003%
CandidatedivisionC	unknown class	unknown order	unknown family			2.73	C1	0.004	0.014%	0.003%
CandidatedivisionC	unknown class	unknown order	unknown family	unknown genus		2.73	C1	0.004	0.014%	0.003%

Chlamydiae					4.01	C1	0.002	0.337%	0.141%
Chlamydiae	Chlamydiae				4.01	C1	0.002	0.337%	0.141%
Chlamydiae	Chlamydiae	Chlamydiales			4.01	C1	0.002	0.337%	0.141%
Chlamydiae	Chlamydiae	Chlamydiales	cvE6		2.71	C1	0.013	0.018%	0.002%
Chlamydiae	Chlamydiae	Chlamydiales	cvE6	unknown genus	2.71	C1	0.013	0.018%	0.002%
Chlamydiae	Chlamydiae	Chlamydiales	Simkaniaceae		3.94	C1	0.004	0.281%	0.130%
Chlamydiae	Chlamydiae	Chlamydiales	Simkaniaceae	Candidatus Rhabdochlamydia	3.85	C1	0.005	0.230%	0.114%
Chloroflexi					3.76	C1	0.002	0.264%	0.071%
Cyanobacteria	Cyanobacteria				3.61	C1	0.010	1.222%	0.751%
Cyanobacteria	Cyanobacteria	SubsectionI	FamilyI	Merismopedia	2.71	C1	0.023	0.014%	0.000%
Cyanobacteria	Melainabacteria	Vampirovibrionales			3.01	C1	0.019	0.034%	0.006%
Cyanobacteria	Melainabacteria	Vampirovibrional	unknown family		3.01	C1	0.019	0.034%	0.006%
Cyanobacteria	Melainabacteria	Vampirovibrional	unknown family	unknown genus	3.01	C1	0.019	0.034%	0.006%
Elusimicrobia					3.73	C1	0.004	0.160%	0.029%
Elusimicrobia	Elusimicrobia				3.73	C1	0.004	0.160%	0.029%
Elusimicrobia	Elusimicrobia	LineageIIb			3.63	C1	0.002	0.126%	0.027%
Elusimicrobia	Elusimicrobia	LineageIIb	unknown family		3.63	C1	0.002	0.126%	0.027%
Elusimicrobia	Elusimicrobia	LineageIIb	unknown family	unknown genus	3.63	C1	0.002	0.126%	0.027%
Firmicutes	Clostridia	Clostridiales	FamilyXII		3.52	C1	0.007	0.098%	0.022%
Firmicutes	Clostridia	Clostridiales	FamilyXII	Acidaminobacter	3.52	C1	0.007	0.098%	0.022%
Firmicutes	Clostridia	Clostridiales	Ruminococcaceae	Anaerobacterium	3.37	C1	0.043	0.068%	0.020%
Firmicutes	Negativicutes	Selenomonadales	Veillonellaceae	Anaerospora	2.83	C1	0.010	0.021%	0.007%
Firmicutes	Negativicutes	Selenomonadales	Veillonellaceae	Pelosinus	3.74	C1	0.018	0.167%	0.085%
Gracilibacteria					3.58	C1	0.013	0.872%	0.276%
Gracilibacteria	unknown class				3.58	C1	0.013	0.872%	0.276%
Gracilibacteria	unknown class	unknown order			3.58	C1	0.013	0.872%	0.276%
Gracilibacteria	unknown class	unknown order	unknown family		3.58	C1	0.013	0.872%	0.276%
Gracilibacteria	unknown class	unknown order	unknown family	unknown genus	3.58	C1	0.013	0.872%	0.276%
Planctomycetes					4.09	C1	0.029	1.359%	0.511%
Planctomycetes	Planctomycetacia	Planctomycetales	Planctomycetaceae	Rhodopirellula	3.00	C1	0.039	0.048%	0.027%
Planctomycetes	Planctomycetacia	Planctomycetales	Planctomycetaceae	Schlesneria	3.26	C1	0.005	0.191%	0.018%
Planctomycetes	Planctomycetacia	Planctomycetales	Planctomycetaceae	unknown genus	3.22	C1	0.050	0.210%	0.042%
**Proteobacteria**					**5.36**	**C1**	**0.002**	**13.755%**	**11.741%**
**Proteobacteria**	**Alphaproteobacteria**				**4.68**	**C1**	**0.002**	**2.285%**	**1.826%**
Proteobacteria	Alphaproteobacte	Caulobacterales			3.59	C1	0.025	0.122%	0.071%
Proteobacteria	Alphaproteobacte	Caulobacterales	Caulobacteraceae		3.45	C1	0.016	0.088%	0.051%
Proteobacteria	Alphaproteobacte	Caulobacterales	Caulobacteraceae	Asticcacaulis	2.85	C1	0.008	0.022%	0.004%
Proteobacteria	Alphaproteobacte	DB1_14			2.99	C1	0.0002	0.028%	0.002%
Proteobacteria	Alphaproteobacte	DB1_14	unknown family		2.99	C1	0.0002	0.028%	0.002%
Proteobacteria	Alphaproteobacte	DB1_14	unknown family	unknown genus	2.99	C1	0.0002	0.028%	0.002%
Proteobacteria	Alphaproteobacte	Rhizobiales			4.31	C1	0.023	1.094%	0.901%
Proteobacteria	Alphaproteobacte	Rhizobiales	Bradyrhizobiaceae		3.57	C1	0.040	0.120%	0.067%
Proteobacteria	Alphaproteobacte	Rhizobiales	Bradyrhizobiaceae	Bosea	3.25	C1	0.020	0.054%	0.010%
Proteobacteria	Alphaproteobacte	Rhizobiales	JG35_K1_AG5		3.35	C1	0.019	0.063%	0.006%
Proteobacteria	Alphaproteobacte	Rhizobiales	JG35_K1_AG5	unknown genus	3.35	C1	0.019	0.063%	0.006%
Proteobacteria	Alphaproteobacte	Rhizobiales	Rhizobiaceae		3.41	C1	0.023	0.175%	0.184%
Proteobacteria	Alphaproteobacte	Rickettsiales	SM2D12		2.43	C1	0.027	0.008%	0.004%
Proteobacteria	Alphaproteobacte	Rickettsiales	SM2D12	unknown genus	2.43	C1	0.027	0.008%	0.004%
Proteobacteria	Alphaproteobacte	Sphingomonadales			4.20	C1	0.020	0.586%	0.307%
Proteobacteria	Alphaproteobacte	Sphingomonadal	Sphingomonadaceae		4.17	C1	0.018	0.554%	0.274%
Proteobacteria	Alphaproteobacte	Sphingomonadal	Sphingomonadaceae	Zymomonas	3.52	C1	0.005	0.098%	0.027%
**Proteobacteria**	**Betaproteobacteria**				**4.92**	**C1**	**0.001**	**5.001%**	**2.600%**
**Proteobacteria**	**Betaproteobacte**	**Burkholderiales**			**4.81**	**C1**	**0.001**	**4.266%**	**2.244%**
Proteobacteria	Betaproteobacte	Burkholderiales	Alcaligenaceae	Achromobacter	2.88	C1	0.032	0.023%	0.000%
**Proteobacteria**	**Betaproteobacte**	**Burkholderiales**	**Comamonadaceae**		**4.58**	**C1**	**0.001**	**2.869%**	**1.588%**
Proteobacteria	Betaproteobacter	Burkholderiales	Comamonadaceae	Giesbergeria	3.30	C1	0.006	0.064%	0.013%
Proteobacteria	Betaproteobacter	Burkholderiales	Comamonadaceae	Limnohabitans	4.00	C1	0.001	0.438%	0.158%
Proteobacteria	Betaproteobacter	Burkholderiales	Comamonadaceae	Multi_affiliation	3.76	C1	0.003	1.153%	0.453%
Proteobacteria	Betaproteobacter	Burkholderiales	Comamonadaceae	Paucibacter	3.11	C1	0.024	0.121%	0.026%
Proteobacteria	Betaproteobacter	Burkholderiales	Comamonadaceae	Rhizobacter	3.33	C1	0.035	0.183%	0.068%
Proteobacteria	Betaproteobacter	Burkholderiales	Comamonadaceae	Sphaerotilus	3.02	C1	0.050	0.033%	0.004%
Proteobacteria	Betaproteobacter	Burkholderiales	Multi_affiliation		4.00	C1	0.0003	0.855%	0.319%
Proteobacteria	Betaproteobacter	Burkholderiales	Multi_affiliation	Multi_affiliation	4.00	C1	0.0003	0.855%	0.319%
Proteobacteria	Betaproteobacter	Burkholderiales	Oxalobacteraceae		4.10	C1	0.003	0.415%	0.222%
Proteobacteria	Betaproteobacter	Burkholderiales	Oxalobacteraceae	Herminiimonas	2.65	C1	0.017	0.013%	0.001%
Proteobacteria	Betaproteobacter	Burkholderiales	Oxalobacteraceae	Undibacterium	3.83	C1	0.008	0.243%	0.124%
Proteobacteria	Betaproteobacter	Multi_affiliation			2.36	C1	0.012	0.040%	0.022%
Proteobacteria	Betaproteobacter	Multi_affiliation	Multi_affiliation		2.36	C1	0.012	0.040%	0.022%
Proteobacteria	Betaproteobacter	Multi_affiliation	Multi_affiliation	Multi_affiliation	2.36	C1	0.012	0.040%	0.022%
Proteobacteria	Betaproteobacter	Neisseriales	Neisseriaceae	Aquaspirillum	2.64	C1	0.010	0.037%	0.003%
Proteobacteria	Betaproteobacter	Neisseriales	Neisseriaceae	unknown genus	3.10	C1	0.014	0.040%	0.009%
Proteobacteria	Betaproteobacter	Rhodocyclales			4.05	C1	0.002	0.372%	0.139%
Proteobacteria	Betaproteobacter	Rhodocyclales	Rhodocyclaceae		4.05	C1	0.002	0.372%	0.139%
Proteobacteria	Betaproteobacter	Rhodocyclales	Rhodocyclaceae	Dechloromonas	3.12	C1	0.007	0.039%	0.008%
Proteobacteria	Betaproteobacter	Rhodocyclales	Rhodocyclaceae	Methyloversatilis	3.35	C1	0.027	0.071%	0.029%
Proteobacteria	Betaproteobacter	Rhodocyclales	Rhodocyclaceae	unknown genus	3.24	C1	0.020	0.081%	0.027%
Proteobacteria	Betaproteobacter	Rhodocyclales	Rhodocyclaceae	Zoogloea	3.22	C1	0.021	0.051%	0.013%
Proteobacteria	Deltaproteobacteria				3.96	C1	0.014	0.686%	0.304%
Proteobacteria	Deltaproteobacte	Myxococcales			3.53	C1	0.021	0.131%	0.042%
Proteobacteria	Deltaproteobacte	Myxococcales	Sandaracinaceae		2.91	C1	0.039	0.024%	0.002%
Proteobacteria	Deltaproteobacte	Myxococcales	Sandaracinaceae	Sandaracinus	2.91	C1	0.039	0.024%	0.002%
Proteobacteria	Deltaproteobacte	Oligoflexales			3.48	C1	0.049	0.163%	0.054%
Proteobacteria	Deltaproteobacte	Oligoflexales	unknown family		3.45	C1	0.012	0.146%	0.038%
Proteobacteria	Deltaproteobacte	Oligoflexales	unknown family	unknown genus	3.45	C1	0.012	0.146%	0.038%
Proteobacteria	Epsilonproteobacteria				3.78	C1	0.029	0.231%	0.103%
Proteobacteria	Epsilonproteobac	Campylobacterales			3.78	C1	0.029	0.231%	0.103%
Proteobacteria	Epsilonproteobac	Campylobacteral	Helicobacteraceae		3.56	C1	0.00002	0.125%	0.005%
Proteobacteria	Epsilonproteobac	Campylobacteral	Helicobacteraceae	Sulfuricurvum	3.42	C1	0.00002	0.081%	0.000%
Proteobacteria	Epsilonproteobac	Campylobacteral	Helicobacteraceae	unknown genus	2.99	C1	0.004	0.044%	0.005%
Proteobacteria	Gammaproteobac	Enterobacteriales			3.73	C1	0.0003	0.166%	0.044%
Proteobacteria	Gammaproteobac	Enterobacteriales	Enterobacteriaceae		3.73	C1	0.0003	0.166%	0.044%

Proteobacteria	Gammaproteobac	Enterobacteriales	Enterobacteriaceae	Yersinia		3.49	C1	0.001	0.093%	0.021%
Proteobacteria	Gammaproteobac	Legionellales				3.75	C1	0.009	0.206%	0.108%
Proteobacteria	Gammaproteobac	Legionellales	Coxiellaceae			3.63	C1	0.040	0.155%	0.078%
Proteobacteria	Gammaproteobac	Legionellales	Coxiellaceae	Rickettsiella		3.61	C1	0.025	0.146%	0.073%
Proteobacteria	Gammaproteobac	Legionellales	Legionellaceae			3.11	C1	0.010	0.050%	0.030%
Proteobacteria	Gammaproteobac	Legionellales	Legionellaceae	Legionella		3.11	C1	0.010	0.050%	0.030%
Proteobacteria	Gammaproteobac	Xanthomonadale	Xanthomonadaceae	Lysobacter		2.84	C1	0.004	0.021%	0.001%
Proteobacteria	Multi_affiliation					3.50	C1	0.002	0.099%	0.025%
Proteobacteria	Multi_affiliation	Multi_affiliation				3.50	C1	0.002	0.099%	0.025%
Proteobacteria	Multi_affiliation	Multi_affiliation	Multi_affiliation			3.50	C1	0.002	0.099%	0.025%
Proteobacteria	Multi_affiliation	Multi_affiliation	Multi_affiliation	Multi_affiliation		3.50	C1	0.002	0.099%	0.025%
SM2F11						3.66	C1	0.002	0.225%	0.085%
SM2F11	unknown class					3.66	C1	0.002	0.225%	0.085%
SM2F11	unknown class	unknown order				3.66	C1	0.002	0.225%	0.085%
SM2F11	unknown class	unknown order	unknown family			3.66	C1	0.002	0.225%	0.085%
SM2F11	unknown class	unknown order	unknown family	unknown genus		3.66	C1	0.002	0.225%	0.085%
Spirochaetae						4.16	C1	0.016	0.504%	0.326%
Spirochaetae	Spirochaetes					4.16	C1	0.016	0.504%	0.326%
Spirochaetae	Spirochaetes	Spirochaetales				4.16	C1	0.016	0.504%	0.326%
Spirochaetae	Spirochaetes	Spirochaetales	Spirochaetaceae			4.15	C1	0.014	0.493%	0.316%
Spirochaetae	Spirochaetes	Spirochaetales	Spirochaetaceae	Spirochaeta2		4.11	C1	0.032	0.384%	0.267%
Verrucomicrobia						3.90	C1	0.000	0.534%	0.311%
Verrucomicrobia	Spartobacteria					3.28	C1	0.007	0.100%	0.054%
Verrucomicrobia	Spartobacteria	Chthoniobacterales				3.28	C1	0.007	0.100%	0.054%
Verrucomicrobia	Spartobacteria	Chthoniobacteral	Chthoniobacteraceae			3.28	C1	0.007	0.100%	0.054%
Verrucomicrobia	Spartobacteria	Chthoniobacteral	Chthoniobacteraceae	Chthoniobacter		3.28	C1	0.007	0.100%	0.054%
Verrucomicrobia	Verrucomicrobiae					3.74	C1	0.018	0.398%	0.244%
Verrucomicrobia	Verrucomicrobiae	Verrucomicrobiales				3.74	C1	0.018	0.398%	0.244%
Verrucomicrobia	Verrucomicrobiae	Verrucomicrobial	Verrucomicrobiaceae			3.74	C1	0.018	0.398%	0.244%
Verrucomicrobia	Verrucomicrobiae	Verrucomicrobial	Verrucomicrobiaceae	Haloferula		3.41	C1	0.009	0.080%	0.033%
**Taxa more abundant in Cluster C2**										
**Actinobacteria**	**Actinobacteria**	**Bifidobacteriales**				**4.88**	**C2**	**0.001**	**1.096%**	**3.019%**
**Actinobacteria**	**Actinobacteria**	**Bifidobacteriales**	**Bifidobacteriaceae**			**4.88**	**C2**	**0.001**	**1.096%**	**3.019%**
Actinobacteria	Actinobacteria	Bifidobacteriales	Bifidobacteriaceae	Aeriscardovia		2.82	C2	0.003	0.005%	0.023%
Actinobacteria	Actinobacteria	Bifidobacteriales	Bifidobacteriaceae	Aeriscardovia	unknown species	2.82	C2	0.003	1.013%	2.920%
**Actinobacteria**	**Actinobacteria**	**Bifidobacteriales**	**Bifidobacteriaceae**	**Bifidobacterium**		**4.86**	**C2**	**0.001**	**1.013%**	**2.920%**
**Actinobacteria**	**Actinobacteria**	**Corynebacteriales**				**5.12**	**C2**	**0.0004**	**3.803%**	**11.183%**
**Actinobacteria**	**Actinobacteria**	**Corynebacteriale**	**Corynebacteriaceae**			**5.10**	**C2**	**0.0004**	**3.525%**	**10.648%**
Actinobacteria	Actinobacteria	Corynebacteriale	Corynebacteriaceae	Corynebacterium		3.72	C2	0.002	0.045%	0.200%
**Actinobacteria**	**Actinobacteria**	**Corynebacteriale**	**Corynebacteriaceae**	**Corynebacterium1**		**5.05**	**C2**	**0.0004**	**3.278%**	**10.125%**
Actinobacteria	Actinobacteria	Corynebacteriale	Corynebacteriaceae	Multi_affiliation		3.87	C2	0.001	0.157%	0.300%
Actinobacteria	Actinobacteria	Corynebacteriale	Dietziaceae			3.85	C2	0.028	0.207%	0.479%
Actinobacteria	Actinobacteria	Corynebacteriale	Dietziaceae	Dietzia		3.85	C2	0.028	0.207%	0.479%
Actinobacteria	Actinobacteria	Corynebacteriale	Multi_affiliation			2.76	C2	0.006	0.069%	0.046%
Actinobacteria	Actinobacteria	Corynebacteriale	Multi_affiliation	Multi_affiliation		2.76	C2	0.006	0.069%	0.046%
Actinobacteria	Actinobacteria	Micrococcales	Brevibacteriaceae			3.44	C2	0.018	0.023%	0.096%
Actinobacteria	Actinobacteria	Micrococcales	Brevibacteriaceae	Brevibacterium		3.44	C2	0.018	0.023%	0.096%
Actinobacteria	Actinobacteria	Micrococcales	Demequinaceae			2.53	C2	0.030	0.003%	0.013%
Actinobacteria	Actinobacteria	Micrococcales	Demequinaceae	Demequina		2.53	C2	0.030	0.003%	0.013%
Actinobacteria	Actinobacteria	Micrococcales	Intrasporangiaceae	Ornithinimicrobium		3.35	C2	0.004	0.026%	0.082%
Actinobacteria	Actinobacteria	Micrococcales	Micrococcaceae	Enteractinococcus		2.52	C2	0.006	0.000%	0.012%
Actinobacteria	Actinobacteria	Micrococcales	Micrococcaceae	Nesterenkonia		2.63	C2	0.030	0.001%	0.015%
Actinobacteria	Actinobacteria	Micrococcales	Micrococcaceae	Rothia		2.99	C2	0.010	0.001%	0.040%
Actinobacteria	Actinobacteria	Micrococcales	Micrococcaceae	unknown genus		3.16	C2	0.001	0.004%	0.051%
Actinobacteria	Actinobacteria	Propionibacterial	Nocardioidaceae	Aeromicrobium		2.83	C2	0.003	0.059%	0.114%
Actinobacteria	Actinobacteria	Propionibacterial	Nocardioidaceae	Nocardioides	Multi_affiliation	3.01	C2	0.044	0.160%	0.360%
**Bacteroidetes**										
Bacteroidetes	BacteroidetesVC2_1Bac22					2.83	C2	0.036	0.167%	0.044%
Bacteroidetes	BacteroidetesVC2	unknown order				2.83	C2	0.036	0.167%	0.044%
Bacteroidetes	BacteroidetesVC2	unknown order	unknown family			2.83	C2	0.036	0.167%	0.044%
Bacteroidetes	BacteroidetesVC2	unknown order	unknown family	unknown genus		2.83	C2	0.036	0.167%	0.044%
Bacteroidetes	Bacteroidia	Bacteroidales	BacteroidalesIncertaeSedis			2.97	C2	0.006	0.007%	0.050%
Bacteroidetes	Bacteroidia	Bacteroidales	BacteroidalesIncertaeSe	Phocaeicola		2.97	C2	0.006	0.007%	0.050%
Bacteroidetes	Bacteroidia	Bacteroidales	p_2534_18B5gutgroup			2.89	C2	0.0002	0.040%	0.043%
Bacteroidetes	Bacteroidia	Bacteroidales	p_2534_18B5gutgroup	unknown genus		2.89	C2	0.0002	0.040%	0.043%
Bacteroidetes	Bacteroidia	Bacteroidales	Porphyromonadaceae	Parabacteroides		2.44	C2	0.035	0.001%	0.010%
Bacteroidetes	Bacteroidia	Bacteroidales	Porphyromonadaceae	Petrimonas		3.03	C2	0.004	0.047%	0.057%
Bacteroidetes	Bacteroidia	Bacteroidales	Prevotellaceae			3.92	C2	0.025	0.172%	0.651%
Bacteroidetes	Bacteroidia	Bacteroidales	Prevotellaceae	Alloprevotella		3.24	C2	0.044	0.022%	0.057%
Bacteroidetes	Bacteroidia	Bacteroidales	Prevotellaceae	Prevotella 1		3.29	C2	0.031	0.033%	0.212%
Bacteroidetes	Bacteroidia	Bacteroidales	Prevotellaceae	Prevotellaceae UCG_001		2.80	C2	0.002	0.012%	0.051%
Bacteroidetes	Bacteroidia	Bacteroidales	Prevotellaceae	Prevotellaceae UCG_003		3.26	C2	0.013	0.077%	0.244%
Bacteroidetes	Bacteroidia	Bacteroidales	Rikenellaceae	dgA_11 gut group		2.90	C2	0.026	0.009%	0.041%
Bacteroidetes	Bacteroidia	Bacteroidales	Rikenellaceae	Rikenellaceae RC9 gut group		3.06	C2	0.0001	0.081%	0.868%
Bacteroidetes	Cytophagia	Cytophagales	Cytophagaceae	Leadbetterella		3.55	C2	0.004	0.029%	0.136%
Bacteroidetes	Cytophagia	Cytophagales	Cytophagaceae	Persicitalea		2.99	C2	0.008	0.009%	0.053%
Bacteroidetes	Cytophagia	OrderIII				3.27	C2	0.033	0.013%	0.068%
Bacteroidetes	Cytophagia	OrderIII	unknown family			3.27	C2	0.033	0.013%	0.068%
Bacteroidetes	Cytophagia	OrderIII	unknown family	unknown genus		3.27	C2	0.033	0.013%	0.068%
Bacteroidetes	Flavobacteriia	Flavobacteriales	Flavobacteriaceae	Moheibacter		3.49	C2	0.005	0.208%	0.364%
Bacteroidetes	Flavobacteriia	Flavobacteriales	Flavobacteriaceae	Subsaxibacter		2.71	C2	0.047	0.017%	0.018%
Bacteroidetes	Sphingobacteriia	Sphingobacteriale	Sphingobacteriaceae	Olivibacter		2.83	C2	0.003	0.045%	0.125%
Bacteroidetes	Sphingobacteriia	Sphingobacteriale	Sphingobacteriaceae	Parapedobacter		2.38	C2	0.049	0.000%	0.008%
Chloroflexi	Thermomicrobia					2.64	C2	0.050	0.038%	0.027%
Chloroflexi	Thermomicrobia	JG30_KF_CM45				2.64	C2	0.050	0.038%	0.027%
Chloroflexi	Thermomicrobia	JG30_KF_CM45	unknown family			2.64	C2	0.050	0.038%	0.027%
Chloroflexi	Thermomicrobia	JG30_KF_CM45	unknown family	unknown genus		2.64	C2	0.050	0.038%	0.027%
Cyanobacteria	Chloroplast					3.65	C2	0.006	0.918%	0.566%
Cyanobacteria	Chloroplast	unknown order				3.65	C2	0.006	0.918%	0.566%
Cyanobacteria	Chloroplast	unknown order	unknown family			3.65	C2	0.006	0.918%	0.566%

Cyanobacteria	Chloroplast	unknown order	unknown family	unknown genus	3.65	C2	0.006	0.918%	0.566%
**Firmicutes**					**5.57**	**C2**	**0.000**	**25.216%**	**42.876%**
**Firmicutes**	**Bacilli**				**5.42**	**C2**	**0.004**	**17.539%**	**26.459%**
Firmicutes	Bacilli	Bacillales	Bacillaceae		3.82	C2	0.001	0.054%	0.308%
Firmicutes	Bacilli	Bacillales	Bacillaceae	Bacillus	2.83	C2	0.036	0.024%	0.039%
Firmicutes	Bacilli	Bacillales	Bacillaceae	Oceanobacillus	3.26	C2	0.007	0.029%	0.078%
Firmicutes	Bacilli	Bacillales	Bacillaceae	Paucisalibacillus	3.03	C2	0.0005	0.000%	0.046%
Firmicutes	Bacilli	Bacillales	Bacillaceae	unknown genus	3.40	C2	0.0001	0.000%	0.089%
Firmicutes	Bacilli	Bacillales	Bacillaceae	Ureibacillus	2.76	C2	0.012	0.001%	0.057%
Firmicutes	Bacilli	Bacillales	Multi_affiliation		3.20	C2	0.0005	5.648%	10.992%
Firmicutes	Bacilli	Bacillales	Multi_affiliation	Multi_affiliation	3.20	C2	0.0005	5.648%	10.992%
Firmicutes	Bacilli	Bacillales	Staphylococcaceae	Aliicoccus	2.49	C2	0.003	0.000%	0.011%
**Firmicutes**	**Bacilli**	**Lactobacillales**			**5.18**	**C2**	**0.001**	**3.697%**	**8.382%**
**Firmicutes**	**Bacilli**	**Lactobacillales**	**Aerococcaceae**		**5.01**	**C2**	**0.001**	**1.335%**	**4.331%**
**Firmicutes**	**Bacilli**	**Lactobacillales**	**Aerococcaceae**	**Aerococcus**	**4.95**	**C2**	**0.001**	**1.060%**	**3.532%**
Firmicutes	Bacilli	Lactobacillales	Aerococcaceae	Facklamia	3.73	C2	0.015	0.132%	0.511%
Firmicutes	Bacilli	Lactobacillales	Aerococcaceae	Globicatella	3.53	C2	0.002	0.030%	0.130%
Firmicutes	Bacilli	Lactobacillales	Carnobacteriaceae	Alloiococcus	2.94	C2	0.011	0.013%	0.234%
Firmicutes	Bacilli	Lactobacillales	Carnobacteriaceae	Lacticigenium	2.86	C2	0.003	0.000%	0.026%
Firmicutes	Bacilli	Lactobacillales	Carnobacteriaceae	Trichococcus	3.18	C2	0.043	0.253%	0.832%
Firmicutes	Bacilli	Lactobacillales	Carnobacteriaceae	unknown genus	4.04	C2	0.045	0.144%	0.436%
Firmicutes	Bacilli	Lactobacillales	Enterococcaceae		4.37	C2	0.032	0.475%	1.733%
Firmicutes	Bacilli	Lactobacillales	Enterococcaceae	Enterococcus	4.37	C2	0.032	0.475%	1.733%
Firmicutes	Bacilli	Lactobacillales	Multi_affiliation		2.84	C2	0.003	0.005%	0.035%
Firmicutes	Bacilli	Lactobacillales	Multi_affiliation	Multi_affiliation	2.84	C2	0.003	0.005%	0.035%
Firmicutes	Bacilli	Lactobacillales	Streptococcaceae		3.80	C2	0.032	1.352%	0.462%
Firmicutes	Bacilli	Lactobacillales	Streptococcaceae	Streptococcus	3.54	C2	0.037	1.191%	0.271%
**Firmicutes**	**Clostridia**				**4.90**	**C2**	**0.025**	**6.838%**	**14.761%**
**Firmicutes**	**Clostridia**	**Clostridiales**			**4.90**	**C2**	**0.025**	**6.838%**	**14.761%**
Firmicutes	Clostridia	Clostridiales	Christensenellaceae		2.91	C2	0.000	0.193%	0.494%
Firmicutes	Clostridia	Clostridiales	Christensenellaceae	Christensenellaceae R_7 group	2.91	C2	0.000	0.193%	0.494%
Firmicutes	Clostridia	Clostridiales	Clostridiaceae1	Multi_affiliation	3.44	C2	0.025	0.071%	0.102%
Firmicutes	Clostridia	Clostridiales	ClostridialesvadinBB60group		2.69	C2	0.012	0.000%	0.017%
Firmicutes	Clostridia	Clostridiales	Defluviitaleaceae		2.72	C2	0.017	0.008%	0.018%
Firmicutes	Clostridia	Clostridiales	Defluviitaleaceae	Defluviitaleaceae UCG_011	2.72	C2	0.017	0.008%	0.018%
Firmicutes	Clostridia	Clostridiales	Eubacteriaceae	Anaerofustis	2.43	C2	0.001	0.000%	0.009%
Firmicutes	Clostridia	Clostridiales	Eubacteriaceae	Eubacterium	2.76	C2	0.008	0.000%	0.021%
Firmicutes	Clostridia	Clostridiales	FamilyXI	Tissierella	2.80	C2	0.017	0.060%	0.161%
Firmicutes	Clostridia	Clostridiales	FamilyXIII		3.62	C2	0.032	0.178%	0.525%
Firmicutes	Clostridia	Clostridiales	FamilyXIII	Eubacterium_brachy group	2.83	C2	0.0005	0.058%	0.105%
Firmicutes	Clostridia	Clostridiales	FamilyXIII	Eubacterium_nodatum group	3.24	C2	0.001	0.010%	0.062%
Firmicutes	Clostridia	Clostridiales	FamilyXIII	Anaerovorax	2.87	C2	0.0212	0.009%	0.026%
Firmicutes	Clostridia	Clostridiales	Lachnospiraceae		4.44	C2	0.0004	1.287%	3.367%
Firmicutes	Clostridia	Clostridiales	Lachnospiraceae	Ruminococcus_gauvreauii group	3.11	C2	0.001	0.011%	0.098%
Firmicutes	Clostridia	Clostridiales	Lachnospiraceae	Anaerosporobacter	3.20	C2	0.040	0.011%	0.056%
Firmicutes	Clostridia	Clostridiales	Lachnospiraceae	Anaerostipes	2.57	C2	0.002	0.001%	0.013%
Firmicutes	Clostridia	Clostridiales	Lachnospiraceae	Blautia	2.87	C2	0.025	0.024%	0.060%
Firmicutes	Clostridia	Clostridiales	Lachnospiraceae	Cellulosilyticum	3.18	C2	0.001	0.114%	0.173%
Firmicutes	Clostridia	Clostridiales	Lachnospiraceae	Coprococcus1	3.26	C2	0.0002	0.031%	0.318%
Firmicutes	Clostridia	Clostridiales	Lachnospiraceae	Eisenbergiella	2.77	C2	0.030	0.002%	0.021%
Firmicutes	Clostridia	Clostridiales	Lachnospiraceae	Howardella	3.38	C2	0.033	0.052%	0.119%
Firmicutes	Clostridia	Clostridiales	Lachnospiraceae	Lachnospiraceae FCS020 group	3.21	C2	0.007	0.021%	0.082%
Firmicutes	Clostridia	Clostridiales	Lachnospiraceae	Lachnospiraceae NK4A136 group	2.78	C2	0.012	0.094%	0.209%
Firmicutes	Clostridia	Clostridiales	Lachnospiraceae	LachnospiraceaeUCG_008	3.32	C2	0.012	0.042%	0.093%
Firmicutes	Clostridia	Clostridiales	Lachnospiraceae	Marvinbryantia	2.81	C2	0.016	0.033%	0.165%
Firmicutes	Clostridia	Clostridiales	Lachnospiraceae	Mobilitalea	2.65	C2	0.012	0.000%	0.015%
Firmicutes	Clostridia	Clostridiales	Lachnospiraceae	Pseudobutyrivibrio	2.71	C2	0.026	0.004%	0.038%
Firmicutes	Clostridia	Clostridiales	Lachnospiraceae	Roseburia	3.73	C2	0.002	0.081%	0.238%
Firmicutes	Clostridia	Clostridiales	Lachnospiraceae	Syntrophococcus	2.84	C2	0.016	0.005%	0.025%
Firmicutes	Clostridia	Clostridiales	Lachnospiraceae	Tyzzerella4	3.26	C2	0.010	0.028%	0.093%
Firmicutes	Clostridia	Clostridiales	Ruminococcaceae	Eubacterium_coprostanoligenes group	2.76	C2	0.006	0.265%	0.546%
Firmicutes	Clostridia	Clostridiales	Ruminococcaceae	CandidatusSoleaferrea	3.04	C2	0.029	0.035%	0.038%
Firmicutes	Clostridia	Clostridiales	Ruminococcaceae	Oscillibacter	2.70	C2	0.001	0.000%	0.018%
Firmicutes	Clostridia	Clostridiales	Ruminococcaceae	Ruminiclostridium 5	2.52	C2	0.012	0.025%	0.070%
Firmicutes	Clostridia	Clostridiales	Ruminococcaceae	Ruminiclostridium 9	2.73	C2	0.012	0.012%	0.020%
Firmicutes	Clostridia	Clostridiales	Ruminococcaceae	Ruminococcaceae NK4A214 group	3.10	C2	0.016	0.097%	0.168%
Firmicutes	Clostridia	Clostridiales	Ruminococcaceae	Ruminococcaceae UCG_001	2.36	C2	0.012	0.000%	0.008%
Firmicutes	Clostridia	Clostridiales	Ruminococcaceae	Ruminococcaceae UCG_004	2.97	C2	0.005	0.006%	0.033%
Firmicutes	Clostridia	Clostridiales	Ruminococcaceae	Ruminococcaceae UCG_005	3.57	C2	0.036	0.787%	2.541%
Firmicutes	Clostridia	Clostridiales	Ruminococcaceae	Ruminococcaceae UCG_010	3.03	C2	0.042	0.105%	0.131%
Firmicutes	Clostridia	Clostridiales	Ruminococcaceae	Ruminococcaceae UCG_011	3.34	C2	0.0004	0.011%	0.076%
Firmicutes	Clostridia	Clostridiales	Ruminococcaceae	unknown genus	2.83	C2	0.016	0.126%	0.326%
Firmicutes	Erysipelotrichia				3.84	C2	0.001	0.056%	0.265%
Firmicutes	Erysipelotrichia	Erysipelotrichales			3.84	C2	0.001	0.056%	0.265%
Firmicutes	Erysipelotrichia	Erysipelotrichales	Erysipelotrichaceae		3.84	C2	0.001	0.056%	0.265%
Firmicutes	Erysipelotrichia	Erysipelotrichales	Erysipelotrichaceae	Faecalitalea	3.19	C2	0.013	0.004%	0.054%
Firmicutes	Erysipelotrichia	Erysipelotrichales	Erysipelotrichaceae	Turicibacter	3.48	C2	0.0002	0.008%	0.109%
Firmicutes	Negativicutes	Selenomonadales	Acidaminococcaceae		4.32	C2	0.001	0.101%	0.740%
Firmicutes	Negativicutes	Selenomonadales	Acidaminococcaceae	Phascolarctobacterium	4.32	C2	0.001	0.101%	0.740%
Firmicutes	Negativicutes	Selenomonadales	Veillonellaceae	Anaerovibrio	3.27	C2	0.005	0.010%	0.064%
Hydrogenedentes					2.75	C2	0.009	0.006%	0.028%
Hydrogenedentes	unknown class				2.75	C2	0.009	0.006%	0.028%
Hydrogenedentes	unknown class	unknown order			2.75	C2	0.009	0.006%	0.028%
Hydrogenedentes	unknown class	unknown order	unknown family		2.75	C2	0.009	0.006%	0.028%
Hydrogenedentes	unknown class	unknown order	unknown family	unknown genus	2.75	C2	0.009	0.006%	0.028%
Multi_affiliation					3.93	C2	0.001	0.124%	0.431%
Multi_affiliation	Multi_affiliation				3.93	C2	0.001	0.124%	0.431%
Multi_affiliation	Multi_affiliation	Multi_affiliation			3.93	C2	0.001	0.124%	0.431%
Multi_affiliation	Multi_affiliation	Multi_affiliation	Multi_affiliation		3.93	C2	0.001	0.124%	0.431%
Multi_affiliation	Multi_affiliation	Multi_affiliation	Multi_affiliation	Multi_affiliation	3.93	C2	0.001	0.124%	0.431%
Planctomycetes	Planctomycetacia	Planctomycetales	Planctomycetaceae	p_1088_a5 gut group	3.37	C2	0.000	0.006%	0.081%

Proteobacteria	Alphaproteobacte	Rhizobiales	Hyphomicrobiaceae			3.51	C2	0.018	0.134%	0.225%
Proteobacteria	Alphaproteobacte	Rhizobiales	Hyphomicrobiaceae	Devosia		3.45	C2	0.006	0.081%	0.205%
Proteobacteria	Alphaproteobacte	Rhizobiales	Phyllobacteriaceae	Aquamicrobium		3.29	C2	0.019	0.051%	0.072%
Proteobacteria	Alphaproteobacte	Rhizobiales	Phyllobacteriaceae	Multi_affiliation		2.80	C2	0.013	0.048%	0.104%
Proteobacteria	Alphaproteobacte	Rhodobacterales	Rhodobacteraceae	Ketogulonicigenium		3.09	C2	0.009	0.009%	0.051%
Proteobacteria	Alphaproteobacte	Rhodobacterales	Rhodobacteraceae	Paracocccus		2.89	C2	0.020	0.027%	0.040%
Proteobacteria	Alphaproteobacte	Rickettsiales	Rickettsiaceae			2.82	C2	0.039	0.059%	0.031%
Proteobacteria	Alphaproteobacte	Rickettsiales	Rickettsiaceae	unknown genus		2.82	C2	0.039	0.059%	0.031%
Proteobacteria	Alphaproteobacte	Sphingomonadal	Erythrobacteraceae			2.88	C2	0.017	0.011%	0.027%
Proteobacteria	Alphaproteobacte	Sphingomonadal	Erythrobacteraceae	Altererythrobacter		2.88	C2	0.017	0.011%	0.027%
Proteobacteria	Betaproteobacter	Burkholderiales	Alcaligenaceae	Oligella	Oligellaureolytica	2.26	C2	0.046	0.020%	0.033%
Proteobacteria	Betaproteobacter	Burkholderiales	Alcaligenaceae	Pusillimonas		3.23	C2	0.009	0.046%	0.064%
Proteobacteria	Betaproteobacter	Burkholderiales	Comamonadaceae	Hydrogenophaga		3.04	C2	0.049	0.008%	0.042%
Proteobacteria	Deltaproteobacte	Desulfuromonadales				2.79	C2	0.012	0.014%	0.051%
Proteobacteria	Deltaproteobacte	Desulfuromonad	GR_WP33_58			2.79	C2	0.012	0.014%	0.051%
Proteobacteria	Deltaproteobacte	Desulfuromonad	GR_WP33_58	unknown genus		2.79	C2	0.012	0.014%	0.051%
Proteobacteria	Epsilonproteobac	Campylobacteral	Campylobacteraceae	Arcobacter		2.08	C2	0.049	0.034%	0.038%
Proteobacteria	Gammaproteobac	Aeromonadales				3.18	C2	0.008	0.014%	0.049%
Proteobacteria	Gammaproteobac	Aeromonadales	Succinivibrionaceae			3.18	C2	0.008	0.014%	0.049%
Proteobacteria	Gammaproteobac	Aeromonadales	Succinivibrionaceae	Ruminobacter		3.08	C2	0.0002	0.005%	0.038%
Proteobacteria	Gammaproteobac	Cellvibrionales	Spongiibacteraceae			2.76	C2	0.001	0.000%	0.023%
Proteobacteria	Gammaproteobac	Cellvibrionales	Spongiibacteraceae	BD1_7clade		2.76	C2	0.001	0.000%	0.023%
Proteobacteria	Gammaproteobac	Oceanospirillales				3.87	C2	0.008	0.113%	0.295%
Proteobacteria	Gammaproteobac	Oceanospirillales	Alcanivoracaceae			1.95	C2	0.025	0.000%	0.003%
Proteobacteria	Gammaproteobac	Oceanospirillales	Alcanivoracaceae	Alcanivorax		1.95	C2	0.025	0.000%	0.003%
Proteobacteria	Gammaproteobac	Oceanospirillales	Halomonadaceae			3.82	C2	0.012	0.111%	0.266%
Proteobacteria	Gammaproteobac	Oceanospirillales	Halomonadaceae	Halomonas		3.82	C2	0.012	0.111%	0.266%
Proteobacteria	Gammaproteobac	Oceanospirillales	Oceanospirillaceae			2.82	C2	0.016	0.002%	0.026%
Proteobacteria	Gammaproteobac	Oceanospirillales	Oceanospirillaceae	Pseudospirillum		2.82	C2	0.016	0.002%	0.026%
Saccharibacteria						3.29	C2	0.035	4.755%	2.700%
Saccharibacteria	unknown class					3.29	C2	0.035	4.755%	2.700%
Saccharibacteria	unknown class	unknown order				3.29	C2	0.035	4.755%	2.700%
Saccharibacteria	unknown class	unknown order	unknown family			3.29	C2	0.035	4.755%	2.700%
Saccharibacteria	unknown class	unknown order	unknown family	Candidatus Saccharimonas		3.12	C2	0.020	0.264%	1.030%
**Tenericutes**						2.58	C2	0.001	0.000%	0.013%
Tenericutes	Mollicutes					2.58	C2	0.001	0.000%	0.013%
Tenericutes	Mollicutes	MollicutesRF9				2.58	C2	0.001	0.000%	0.013%
Tenericutes	Mollicutes	MollicutesRF9	unknown family			2.58	C2	0.001	0.000%	0.013%
Tenericutes	Mollicutes	MollicutesRF9	unknown family	unknown genus		2.58	C2	0.001	0.000%	0.013%
Verrucomicrobia	Verrucomicrobiae	Verrucomicrobial	Verrucomicrobiaceae	Akkermansia		2.99	C2	0.002	0.040%	0.072%

Table S4. Differentially abundant OTUs between Clusters C1 and C2 at D0 as determined by the DESeq2 R package.
Results include the Log2(C2/C1) abundance ratio for OTU with a padj<0.05.

OTU	Phylum	Class	Order	Family	Genus	Species	log2 fold change (C2/C1)	padj	abundance at D0
Cluster_969	Actinobacteria	Actinobacteria	Bifidobacteriales	Bifidobacteriaceae	Aeriscardovia	unknown species	4.41	3.85E-04	0.00015
Cluster_37	Actinobacteria	Actinobacteria	Bifidobacteriales	Bifidobacteriaceae	Bifidobacterium	Bifidobacterium merycicum	4.51	6.01E-09	0.00378
Cluster_128	Actinobacteria	Actinobacteria	Bifidobacteriales	Bifidobacteriaceae	Bifidobacterium	Multi-affiliation	4.88	6.06E-08	0.00143
Cluster_13	Actinobacteria	Actinobacteria	Bifidobacteriales	Bifidobacteriaceae	Bifidobacterium	Multi-affiliation	3.09	2.20E-04	0.01365
Cluster_249	Actinobacteria	Actinobacteria	Bifidobacteriales	Bifidobacteriaceae	unknown genus	unknown species	3.20	1.56E-03	0.00053
Cluster_895	Actinobacteria	Actinobacteria	Corynebacteriales	Corynebacteriaceae	Corynebacterium	Corynebacterium epidermidicanis	3.24	2.50E-02	0.00018
Cluster_111	Actinobacteria	Actinobacteria	Corynebacteriales	Corynebacteriaceae	Corynebacterium	Corynebacterium humireducens NBRC 106098 =	4.80	4.53E-05	0.00091
Cluster_745	Actinobacteria	Actinobacteria	Corynebacteriales	Corynebacteriaceae	Corynebacterium	Corynebacterium vitaeruminis	4.09	2.41E-02	0.00009
Cluster_791	Actinobacteria	Actinobacteria	Corynebacteriales	Corynebacteriaceae	Corynebacterium	unknown species	5.23	5.35E-03	0.00014
Cluster_10	Actinobacteria	Actinobacteria	Corynebacteriales	Corynebacteriaceae	Corynebacterium 1	Corynebacterium camporealensis	5.54	4.68E-09	0.01504
Cluster_29	Actinobacteria	Actinobacteria	Corynebacteriales	Corynebacteriaceae	Corynebacterium 1	Corynebacterium casei LMG S-19264	3.99	1.63E-06	0.00749
Cluster_59	Actinobacteria	Actinobacteria	Corynebacteriales	Corynebacteriaceae	Corynebacterium 1	Corynebacterium sp.	5.09	1.13E-08	0.00225
Cluster_914	Actinobacteria	Actinobacteria	Corynebacteriales	Corynebacteriaceae	Corynebacterium 1	Multi-affiliation	4.46	3.57E-02	0.00007
Cluster_788	Actinobacteria	Actinobacteria	Corynebacteriales	Corynebacteriaceae	Corynebacterium 1	Multi-affiliation	6.02	3.28E-04	0.00012
Cluster_304	Actinobacteria	Actinobacteria	Corynebacteriales	Corynebacteriaceae	Corynebacterium 1	Multi-affiliation	5.96	1.07E-03	0.00038
Cluster_294	Actinobacteria	Actinobacteria	Corynebacteriales	Corynebacteriaceae	Corynebacterium 1	Multi-affiliation	2.56	2.80E-02	0.00048
Cluster_50	Actinobacteria	Actinobacteria	Corynebacteriales	Corynebacteriaceae	Corynebacterium 1	Multi-affiliation	4.25	1.92E-07	0.00235
Cluster_16	Actinobacteria	Actinobacteria	Corynebacteriales	Corynebacteriaceae	Corynebacterium 1	Multi-affiliation	4.27	7.33E-08	0.00535
Cluster_12	Actinobacteria	Actinobacteria	Corynebacteriales	Corynebacteriaceae	Corynebacterium 1	Multi-affiliation	4.52	4.24E-06	0.01143
Cluster_1052	Actinobacteria	Actinobacteria	Corynebacteriales	Corynebacteriaceae	Corynebacterium 1	unknown species	6.42	1.00E-04	0.00020
Cluster_662	Actinobacteria	Actinobacteria	Corynebacteriales	Corynebacteriaceae	Corynebacterium 1	unknown species	4.46	3.63E-03	0.00025
Cluster_652	Actinobacteria	Actinobacteria	Corynebacteriales	Corynebacteriaceae	Corynebacterium 1	unknown species	5.33	1.83E-05	0.00030
Cluster_578	Actinobacteria	Actinobacteria	Corynebacteriales	Corynebacteriaceae	Corynebacterium 1	unknown species	4.76	3.43E-04	0.00035
Cluster_90	Actinobacteria	Actinobacteria	Corynebacteriales	Corynebacteriaceae	Corynebacterium 1	unknown species	3.41	3.17E-03	0.00113
Cluster_32	Actinobacteria	Actinobacteria	Corynebacteriales	Corynebacteriaceae	Corynebacterium 1	unknown species	3.87	7.79E-06	0.00507
Cluster_19	Actinobacteria	Actinobacteria	Corynebacteriales	Corynebacteriaceae	Corynebacterium 1	unknown species	2.71	1.19E-03	0.01501
Cluster_1586	Actinobacteria	Actinobacteria	Corynebacteriales	Corynebacteriaceae	Multi-affiliation	Multi-affiliation	4.89	1.16E-03	0.00010
Cluster_51	Actinobacteria	Actinobacteria	Corynebacteriales	Corynebacteriaceae	Multi-affiliation	Multi-affiliation	4.70	3.07E-07	0.00199
Cluster_74	Actinobacteria	Actinobacteria	Corynebacteriales	Dietziaceae	Dietzia	Multi-affiliation	3.77	1.61E-05	0.00155
Cluster_88	Actinobacteria	Actinobacteria	Corynebacteriales	Dietziaceae	Dietzia	Multi-affiliation	3.60	4.17E-04	0.00198
Cluster_825	Actinobacteria	Actinobacteria	Corynebacteriales	Multi-affiliation	Multi-affiliation	Multi-affiliation	6.05	2.43E-05	0.00017
Cluster_2438	Actinobacteria	Actinobacteria	Frankiales	Geodermatophilaceae	unknown genus	unknown species	3.95	4.95E-02	0.00010
Cluster_356	Actinobacteria	Actinobacteria	Micrococcales	Brevibacteriaceae	Brevibacterium	unknown species	3.22	9.95E-03	0.00057
Cluster_1050	Actinobacteria	Actinobacteria	Micrococcales	Demequinaceae	Demequina	unknown species	4.10	1.59E-02	0.00017
Cluster_73	Actinobacteria	Actinobacteria	Micrococcales	Dermabacteraceae	Brachybacterium	Multi-affiliation	3.00	2.29E-04	0.00509
Cluster_123	Actinobacteria	Actinobacteria	Micrococcales	Intrasporangiaceae	Ornithinimicrobium	Multi-affiliation	3.53	4.50E-04	0.00071
Cluster_1201	Actinobacteria	Actinobacteria	Micrococcales	Jonesiaceae	Jonesia	Jonesia denitrificans	3.18	5.19E-03	0.00015
Cluster_880	Actinobacteria	Actinobacteria	Micrococcales	Microbacteriaceae	Leucobacter	unknown species	2.48	2.96E-02	0.00033
Cluster_991	Actinobacteria	Actinobacteria	Micrococcales	Microbacteriaceae	Pseudoclavibacter	Pseudoclavibacter chungangensis	3.05	4.95E-02	0.00017
Cluster_25	Actinobacteria	Actinobacteria	Micrococcales	Micrococcaceae	Arthrobacter	Multi-affiliation	3.66	2.38E-06	0.00664
Cluster_1779	Actinobacteria	Actinobacteria	Micrococcales	Micrococcaceae	Enteractinococcus	Actinomycetales bacterium SSCS15	4.90	1.88E-02	0.00005
Cluster_413	Actinobacteria	Actinobacteria	Micrococcales	Micrococcaceae	Kocuria	Multi-affiliation	3.05	3.45E-03	0.00064
Cluster_250	Actinobacteria	Actinobacteria	Micrococcales	Micrococcaceae	Multi-affiliation	Multi-affiliation	4.91	4.24E-06	0.00099
Cluster_1203	Actinobacteria	Actinobacteria	Micrococcales	Micrococcaceae	Nesterenkonia	Multi-affiliation	4.86	1.79E-02	0.00015
Cluster_409	Actinobacteria	Actinobacteria	Micrococcales	Micrococcaceae	Rothia	Multi-affiliation	4.81	3.86E-03	0.00020
Cluster_540	Actinobacteria	Actinobacteria	Micrococcales	Micrococcaceae	unknown genus	unknown species	6.06	4.72E-06	0.00028
Cluster_102	Actinobacteria	Actinobacteria	Multi-affiliation	Multi-affiliation	Multi-affiliation	Multi-affiliation	3.88	1.65E-04	0.00119
Cluster_614	Actinobacteria	Actinobacteria	PeM15	unknown family	unknown genus	unknown species	2.00	2.31E-02	0.00021
Cluster_784	Actinobacteria	Actinobacteria	Propionibacteriales	Nocardioidaceae	Aeromicrobium	Multi-affiliation	3.31	2.99E-02	0.00014
Cluster_602	Actinobacteria	Actinobacteria	Propionibacteriales	Nocardioidaceae	Aeromicrobium	Multi-affiliation	2.90	6.30E-03	0.00035
Cluster_394	Actinobacteria	Actinobacteria	Propionibacteriales	Nocardioidaceae	Nocardioides	Multi-affiliation	3.44	3.38E-02	0.00031
Cluster_696	Actinobacteria	Actinobacteria	Propionibacteriales	Nocardioidaceae	Nocardioides	Multi-affiliation	3.28	2.79E-02	0.00032
Cluster_108	Actinobacteria	Actinobacteria	Propionibacteriales	Nocardioidaceae	Nocardioides	unknown species	3.10	1.51E-03	0.00136
Cluster_1542	Actinobacteria	Coriobacteriia	Coriobacteriales	Coriobacteriaceae	Olsenella	unknown species	5.43	6.62E-03	0.00007
Cluster_1018	Bacteroidetes	Bacteroidia	Bacteroidales	Bacteroidaceae	Bacteroides	unknown species	5.11	1.16E-03	0.00008
Cluster_1353	Bacteroidetes	Bacteroidia	Bacteroidales	Bacteroidaceae	Bacteroides	unknown species	5.34	3.21E-04	0.00010
Cluster_853	Bacteroidetes	Bacteroidia	Bacteroidales	Bacteroidaceae	Bacteroides	unknown species	3.74	2.01E-02	0.00017
Cluster_846	Bacteroidetes	Bacteroidia	Bacteroidales	Bacteroidaceae	Bacteroides	unknown species	5.16	1.00E-04	0.00017
Cluster_785	Bacteroidetes	Bacteroidia	Bacteroidales	Bacteroidaceae	Bacteroides	unknown species	5.44	6.16E-05	0.00019
Cluster_516	Bacteroidetes	Bacteroidia	Bacteroidales	Bacteroidaceae	Bacteroides	unknown species	5.02	3.54E-04	0.00032
Cluster_342	Bacteroidetes	Bacteroidia	Bacteroidales	Bacteroidaceae	Bacteroides	unknown species	3.85	3.08E-03	0.00039
Cluster_308	Bacteroidetes	Bacteroidia	Bacteroidales	Bacteroidaceae	Bacteroides	unknown species	4.91	5.05E-06	0.00048
Cluster_571	Bacteroidetes	Bacteroidia	Bacteroidales	Bacteroidaceae	Bacteroides	unknown species	4.27	1.63E-03	0.00048
Cluster_306	Bacteroidetes	Bacteroidia	Bacteroidales	Bacteroidaceae	Bacteroides	unknown species	3.86	4.97E-03	0.00051
Cluster_309	Bacteroidetes	Bacteroidia	Bacteroidales	Bacteroidaceae	Bacteroides	unknown species	3.24	2.63E-03	0.00064
Cluster_216	Bacteroidetes	Bacteroidia	Bacteroidales	Bacteroidaceae	Bacteroides	unknown species	4.06	7.73E-06	0.00090
Cluster_143	Bacteroidetes	Bacteroidia	Bacteroidales	Bacteroidaceae	Bacteroides	unknown species	4.57	7.33E-08	0.00233
Cluster_1330	Bacteroidetes	Bacteroidia	Bacteroidales	Bacteroidales Incertae	Phocaeicola	unknown species	5.13	4.97E-03	0.00012

Cluster_2427	Bacteroidetes	Bacteroidia	Bacteroidales	Bacteroidales S24-7 grc	unknown genus	unknown species	4.66	1.46E-02	0.00009
Cluster_665	Bacteroidetes	Bacteroidia	Bacteroidales	Bacteroidales S24-7 grc	unknown genus	unknown species	3.54	1.37E-02	0.00011
Cluster_797	Bacteroidetes	Bacteroidia	Bacteroidales	Bacteroidales S24-7 grc	unknown genus	unknown species	4.11	1.31E-03	0.00011
Cluster_1068	Bacteroidetes	Bacteroidia	Bacteroidales	Bacteroidales S24-7 grc	unknown genus	unknown species	6.30	2.32E-04	0.00018
Cluster_495	Bacteroidetes	Bacteroidia	Bacteroidales	Bacteroidales S24-7 grc	unknown genus	unknown species	5.82	2.76E-05	0.00035
Cluster_232	Bacteroidetes	Bacteroidia	Bacteroidales	Bacteroidales S24-7 grc	unknown genus	unknown species	5.16	1.61E-05	0.00083
Cluster_775	Bacteroidetes	Bacteroidia	Bacteroidales	p-2534-18B5 gut group	unknown genus	unknown species	5.92	9.74E-06	0.00013
Cluster_951	Bacteroidetes	Bacteroidia	Bacteroidales	Porphyromonadaceae	Parabacteroides	unknown species	4.20	9.65E-03	0.00007
Cluster_286	Bacteroidetes	Bacteroidia	Bacteroidales	Prevotellaceae	Alloprevotella	unknown species	3.31	6.04E-03	0.00040
Cluster_375	Bacteroidetes	Bacteroidia	Bacteroidales	Prevotellaceae	Prevotella 1	unknown species	5.39	4.53E-05	0.00013
Cluster_565	Bacteroidetes	Bacteroidia	Bacteroidales	Prevotellaceae	Prevotella 1	unknown species	4.40	4.17E-04	0.00034
Cluster_339	Bacteroidetes	Bacteroidia	Bacteroidales	Prevotellaceae	Prevotella 1	unknown species	4.08	2.11E-03	0.00060
Cluster_828	Bacteroidetes	Bacteroidia	Bacteroidales	Prevotellaceae	Prevotellaceae UCG-	unknown species	4.48	5.38E-03	0.00010
Cluster_252	Bacteroidetes	Bacteroidia	Bacteroidales	Prevotellaceae	Prevotellaceae UCG-	unknown species	4.21	1.14E-04	0.00026
Cluster_573	Bacteroidetes	Bacteroidia	Bacteroidales	Prevotellaceae	Prevotellaceae UCG-	unknown species	3.57	1.19E-03	0.00037
Cluster_313	Bacteroidetes	Bacteroidia	Bacteroidales	Prevotellaceae	Prevotellaceae UCG-	unknown species	2.57	3.67E-02	0.00045
Cluster_997	Bacteroidetes	Bacteroidia	Bacteroidales	Prevotellaceae	Prevotellaceae UCG-	unknown species	3.89	3.53E-03	0.00014
Cluster_1493	Bacteroidetes	Bacteroidia	Bacteroidales	Rikenellaceae	Alistipes	unknown species	4.45	2.54E-04	0.00018
Cluster_751	Bacteroidetes	Bacteroidia	Bacteroidales	Rikenellaceae	Alistipes	unknown species	3.28	1.05E-02	0.00023
Cluster_475	Bacteroidetes	Bacteroidia	Bacteroidales	Rikenellaceae	Alistipes	unknown species	4.04	1.23E-03	0.00028
Cluster_276	Bacteroidetes	Bacteroidia	Bacteroidales	Rikenellaceae	Alistipes	unknown species	3.55	8.79E-03	0.00048
Cluster_1711	Bacteroidetes	Bacteroidia	Bacteroidales	Rikenellaceae	dgA-11 gut group	unknown species	3.68	1.80E-02	0.00009
Cluster_1945	Bacteroidetes	Bacteroidia	Bacteroidales	Rikenellaceae	dgA-11 gut group	unknown species	4.07	8.79E-03	0.00014
Cluster_2591	Bacteroidetes	Bacteroidia	Bacteroidales	Rikenellaceae	Rikenellaceae RC9 gut	unknown species	5.34	9.20E-03	0.00008
Cluster_1391	Bacteroidetes	Bacteroidia	Bacteroidales	Rikenellaceae	Rikenellaceae RC9 gut	unknown species	5.35	5.85E-04	0.00009
Cluster_1186	Bacteroidetes	Bacteroidia	Bacteroidales	Rikenellaceae	Rikenellaceae RC9 gut	unknown species	5.15	1.26E-03	0.00009
Cluster_1328	Bacteroidetes	Bacteroidia	Bacteroidales	Rikenellaceae	Rikenellaceae RC9 gut	unknown species	4.89	6.55E-03	0.00010
Cluster_1677	Bacteroidetes	Bacteroidia	Bacteroidales	Rikenellaceae	Rikenellaceae RC9 gut	unknown species	4.49	3.60E-03	0.00011
Cluster_2826	Bacteroidetes	Bacteroidia	Bacteroidales	Rikenellaceae	Rikenellaceae RC9 gut	unknown species	5.37	1.27E-03	0.00015
Cluster_1051	Bacteroidetes	Bacteroidia	Bacteroidales	Rikenellaceae	Rikenellaceae RC9 gut	unknown species	4.07	1.36E-02	0.00016
Cluster_810	Bacteroidetes	Bacteroidia	Bacteroidales	Rikenellaceae	Rikenellaceae RC9 gut	unknown species	4.48	3.28E-03	0.00016
Cluster_632	Bacteroidetes	Bacteroidia	Bacteroidales	Rikenellaceae	Rikenellaceae RC9 gut	unknown species	5.86	7.68E-06	0.00017
Cluster_1322	Bacteroidetes	Bacteroidia	Bacteroidales	Rikenellaceae	Rikenellaceae RC9 gut	unknown species	6.35	1.31E-03	0.00017
Cluster_1397	Bacteroidetes	Bacteroidia	Bacteroidales	Rikenellaceae	Rikenellaceae RC9 gut	unknown species	3.71	3.26E-02	0.00018
Cluster_660	Bacteroidetes	Bacteroidia	Bacteroidales	Rikenellaceae	Rikenellaceae RC9 gut	unknown species	5.71	1.05E-04	0.00022
Cluster_663	Bacteroidetes	Bacteroidia	Bacteroidales	Rikenellaceae	Rikenellaceae RC9 gut	unknown species	4.23	7.34E-03	0.00023
Cluster_994	Bacteroidetes	Bacteroidia	Bacteroidales	Rikenellaceae	Rikenellaceae RC9 gut	unknown species	6.66	1.11E-06	0.00025
Cluster_669	Bacteroidetes	Bacteroidia	Bacteroidales	Rikenellaceae	Rikenellaceae RC9 gut	unknown species	6.24	6.06E-08	0.00032
Cluster_385	Bacteroidetes	Bacteroidia	Bacteroidales	Rikenellaceae	Rikenellaceae RC9 gut	unknown species	6.54	3.49E-09	0.00035
Cluster_471	Bacteroidetes	Bacteroidia	Bacteroidales	Rikenellaceae	Rikenellaceae RC9 gut	unknown species	5.02	4.78E-06	0.00039
Cluster_262	Bacteroidetes	Bacteroidia	Bacteroidales	Rikenellaceae	Rikenellaceae RC9 gut	unknown species	4.88	9.10E-06	0.00056
Cluster_280	Bacteroidetes	Bacteroidia	Bacteroidales	Rikenellaceae	Rikenellaceae RC9 gut	unknown species	4.32	1.13E-04	0.00064
Cluster_141	Bacteroidetes	Cytophagia	Cytophagales	Cytophagaceae	Leadbetterella	byssophila	4.13	1.00E-04	0.00105
Cluster_1034	Bacteroidetes	Cytophagia	Cytophagales	Cytophagaceae	Persicitalea	unknown species	4.75	9.80E-03	0.00008
Cluster_429	Bacteroidetes	Cytophagia	Cytophagales	Cytophagaceae	Persicitalea	unknown species	6.72	1.21E-08	0.00025
Cluster_658	Bacteroidetes	Cytophagia	Cytophagales	Cytophagaceae	unknown genus	unknown species	4.90	3.14E-02	0.00005
Cluster_396	Bacteroidetes	Cytophagia	Order III	unknown family	unknown genus	unknown species	3.69	9.03E-03	0.00053
Cluster_868	Bacteroidetes	Flavobacteriia	Flavobacteriales	Cryomorphaceae	Fluviicola	unknown species	3.80	1.11E-02	0.00011
Cluster_82	Bacteroidetes	Flavobacteriia	Flavobacteriales	Flavobacteriaceae	Flavobacterium	Multi-affiliation	4.57	3.11E-08	0.00174
Cluster_1231	Bacteroidetes	Flavobacteriia	Flavobacteriales	Flavobacteriaceae	Flavobacterium	unknown species	4.58	1.08E-02	0.00016
Cluster_1402	Bacteroidetes	Flavobacteriia	Flavobacteriales	Flavobacteriaceae	Flavobacterium	unknown species	3.48	3.56E-02	0.00020
Cluster_892	Bacteroidetes	Flavobacteriia	Flavobacteriales	Flavobacteriaceae	Flavobacterium	unknown species	4.41	2.21E-02	0.00021
Cluster_655	Bacteroidetes	Flavobacteriia	Flavobacteriales	Flavobacteriaceae	Flavobacterium	unknown species	4.30	2.18E-03	0.00023
Cluster_437	Bacteroidetes	Flavobacteriia	Flavobacteriales	Flavobacteriaceae	Flavobacterium	unknown species	4.12	1.16E-03	0.00026
Cluster_307	Bacteroidetes	Flavobacteriia	Flavobacteriales	Flavobacteriaceae	Flavobacterium	unknown species	3.19	1.06E-02	0.00036
Cluster_87	Bacteroidetes	Flavobacteriia	Flavobacteriales	Flavobacteriaceae	Flavobacterium	unknown species	-1.35	1.33E-02	0.00129
Cluster_388	Bacteroidetes	Flavobacteriia	Flavobacteriales	Flavobacteriaceae	Moheibacter	unknown species	3.96	4.24E-04	0.00046
Cluster_75	Bacteroidetes	Flavobacteriia	Flavobacteriales	Flavobacteriaceae	Moheibacter	unknown species	3.37	2.05E-03	0.00138
Cluster_1147	Bacteroidetes	Flavobacteriia	Flavobacteriales	Flavobacteriaceae	Subsaxibacter	unknown species	4.82	1.40E-03	0.00017
Cluster_18	Bacteroidetes	Flavobacteriia	Flavobacteriales	Flavobacteriaceae	unknown genus	Multi-affiliation	1.81	2.61E-02	0.00771
Cluster_1200	Bacteroidetes	Sphingobacterii	Sphingobacteriales	Chitinophagaceae	Taibaiella	unknown species	4.45	2.80E-02	0.00019
Cluster_610	Bacteroidetes	Sphingobacterii	Sphingobacteriales	Chitinophagaceae	Taibaiella	unknown species	5.91	1.18E-04	0.00020
Cluster_622	Bacteroidetes	Sphingobacterii	Sphingobacteriales	Chitinophagaceae	Taibaiella	unknown species	3.50	2.11E-02	0.00029
Cluster_1246	Bacteroidetes	Sphingobacterii	Sphingobacteriales	Sphingobacteriaceae	Olivibacter	unknown species	3.41	3.48E-02	0.00026
Cluster_604	Bacteroidetes	Sphingobacterii	Sphingobacteriales	Sphingobacteriaceae	Olivibacter	unknown species	3.52	3.56E-02	0.00029
Cluster_468	Bacteroidetes	Sphingobacterii	Sphingobacteriales	Sphingobacteriaceae	Sphingobacterium	bacterium CC-YY411	4.52	2.12E-03	0.00036
Cluster_1292	Bacteroidetes	Sphingobacterii	Sphingobacteriales	Sphingobacteriaceae	Sphingobacterium	Multi-affiliation	3.78	3.57E-02	0.00014
Cluster_220	Bacteroidetes	Sphingobacterii	Sphingobacteriales	Sphingobacteriaceae	Sphingobacterium	alimentarium	5.26	2.88E-08	0.00081
Cluster_1006	Bacteroidetes	Sphingobacterii	Sphingobacteriales	Sphingobacteriaceae	Sphingobacterium	wenxiniae	5.24	1.57E-02	0.00019
Cluster_1087	Bacteroidetes	Sphingobacterii	Sphingobacteriales	Sphingobacteriaceae	Sphingobacterium	unknown species	6.13	1.92E-05	0.00024
Cluster_285	Bacteroidetes	Sphingobacterii	Sphingobacteriales	Sphingobacteriaceae	Sphingobacterium	unknown species	3.02	4.06E-03	0.00076
Cluster_179	Bacteroidetes	Sphingobacterii	Sphingobacteriales	Sphingobacteriaceae	Sphingobacterium	unknown species	2.40	3.24E-02	0.00107
Cluster_170	Cyanobacteria	Chloroplast	unknown order	unknown family	unknown genus	Abies fabri	4.02	2.76E-03	0.00124
Cluster_434	Deinococcus-The	Deinococci	Deinococcales	Trueperaceae	Truepera	unknown species	4.28	5.40E-03	0.00017
Cluster_1624	Fibrobacteres	Fibrobacteria	Fibrobacterales	Fibrobacteraceae	Fibrobacter	unknown species	3.98	2.94E-02	0.00008
Cluster_1303	Firmicutes	Bacilli	Bacillales	Bacillaceae	Bacillus	unknown species	4.35	5.12E-03	0.00009
Cluster_1707	Firmicutes	Bacilli	Bacillales	Bacillaceae	Oceanobacillus	Multi-affiliation	3.94	3.74E-02	0.00004
Cluster_1734	Firmicutes	Bacilli	Bacillales	Bacillaceae	Oceanobacillus	Multi-affiliation	3.95	2.79E-02	0.00009
Cluster_1610	Firmicutes	Bacilli	Bacillales	Bacillaceae	Paucisalibacillus	unknown species	5.30	3.03E-03	0.00021
Cluster_737	Firmicutes	Bacilli	Bacillales	Bacillaceae	unknown genus	unknown species	7.49	4.44E-06	0.00035
Cluster_2536	Firmicutes	Bacilli	Bacillales	Bacillaceae	Ureibacillus	unknown species	6.47	4.30E-04	0.00016
Cluster_1207	Firmicutes	Bacilli	Bacillales	Bacillaceae	Ureibacillus	unknown species	4.03	3.77E-02	0.00018

Cluster_3510	Firmicutes	Bacilli	Bacillales	Multi-affiliation	Multi-affiliation	Multi-affiliation	3.75	2.01E-02	0.00013
Cluster_1525	Firmicutes	Bacilli	Bacillales	Multi-affiliation	Multi-affiliation	Multi-affiliation	4.01	4.26E-03	0.00015
Cluster_798	Firmicutes	Bacilli	Bacillales	Multi-affiliation	Multi-affiliation	Multi-affiliation	5.88	2.38E-06	0.00017
Cluster_904	Firmicutes	Bacilli	Bacillales	Multi-affiliation	Multi-affiliation	Multi-affiliation	7.13	5.61E-06	0.00030
Cluster_5	Firmicutes	Bacilli	Bacillales	Multi-affiliation	Multi-affiliation	Multi-affiliation	2.51	2.80E-04	0.02953
Cluster_1	Firmicutes	Bacilli	Bacillales	Multi-affiliation	Multi-affiliation	Multi-affiliation	2.18	2.44E-03	0.05354
Cluster_204	Firmicutes	Bacilli	Bacillales	Planococcaceae	Lysinibacillus	Multi-affiliation	2.49	2.80E-02	0.00086
Cluster_1191	Firmicutes	Bacilli	Bacillales	Planococcaceae	Solibacillus	Bacillus cecembensis	4.60	7.34E-03	0.00009
Cluster_1930	Firmicutes	Bacilli	Bacillales	Planococcaceae	Solibacillus	unknown species	3.49	2.16E-02	0.00013
Cluster_58	Firmicutes	Bacilli	Bacillales	Planococcaceae	Solibacillus	unknown species	2.93	5.53E-04	0.00265
Cluster_376	Firmicutes	Bacilli	Bacillales	Planococcaceae	unknown genus	unknown species	3.06	2.99E-02	0.00064
Cluster_185	Firmicutes	Bacilli	Bacillales	Planococcaceae	unknown genus	unknown species	6.20	2.31E-06	0.00082
Cluster_1136	Firmicutes	Bacilli	Bacillales	Staphylococcaceae	Aliicoccus	Multi-affiliation	3.76	1.96E-02	0.00005
Cluster_118	Firmicutes	Bacilli	Bacillales	Staphylococcaceae	Jeotgalicoccus	Multi-affiliation	3.81	2.42E-05	0.00107
Cluster_55	Firmicutes	Bacilli	Bacillales	Staphylococcaceae	Jeotgalicoccus	Multi-affiliation	2.61	2.17E-03	0.00247
Cluster_2148	Firmicutes	Bacilli	Bacillales	Staphylococcaceae	Staphylococcus	Multi-affiliation	4.97	2.53E-04	0.00016
Cluster_679	Firmicutes	Bacilli	Bacillales	Staphylococcaceae	Staphylococcus	Multi-affiliation	2.47	4.10E-02	0.00020
Cluster_534	Firmicutes	Bacilli	Bacillales	Staphylococcaceae	Staphylococcus	Multi-affiliation	4.52	3.92E-03	0.00035
Cluster_319	Firmicutes	Bacilli	Bacillales	Staphylococcaceae	Staphylococcus	Multi-affiliation	5.02	3.91E-05	0.00051
Cluster_259	Firmicutes	Bacilli	Bacillales	Staphylococcaceae	Staphylococcus	Multi-affiliation	3.29	8.87E-03	0.00061
Cluster_193	Firmicutes	Bacilli	Bacillales	Staphylococcaceae	Staphylococcus	Multi-affiliation	4.61	1.25E-03	0.00100
Cluster_7	Firmicutes	Bacilli	Bacillales	Staphylococcaceae	Staphylococcus	Multi-affiliation	2.82	2.53E-03	0.02074
Cluster_57	Firmicutes	Bacilli	Lactobacillales	Aerococcaceae	Aerococcus	Aerococcus suis	4.28	6.79E-06	0.00207
Cluster_254	Firmicutes	Bacilli	Lactobacillales	Aerococcaceae	Aerococcus	Aerococcus vaginalis	5.28	1.12E-05	0.00041
Cluster_6	Firmicutes	Bacilli	Lactobacillales	Aerococcaceae	Aerococcus	Multi-affiliation	3.16	2.03E-04	0.02232
Cluster_66	Firmicutes	Bacilli	Lactobacillales	Aerococcaceae	Facklamia	Facklamia sp.	3.50	2.27E-05	0.00176
Cluster_110	Firmicutes	Bacilli	Lactobacillales	Aerococcaceae	Facklamia	Multi-affiliation	3.59	3.28E-04	0.00111
Cluster_351	Firmicutes	Bacilli	Lactobacillales	Aerococcaceae	Facklamia	unknown species	3.83	1.12E-03	0.00036
Cluster_71	Firmicutes	Bacilli	Lactobacillales	Aerococcaceae	Globicatella	unknown species	3.89	2.29E-04	0.00147
Cluster_349	Firmicutes	Bacilli	Lactobacillales	Aerococcaceae	Ignavigranum	unknown species	2.98	4.48E-02	0.00035
Cluster_783	Firmicutes	Bacilli	Lactobacillales	Carnobacteriaceae	Alloiococcus	bacterium #1 from	4.92	1.18E-03	0.00014
Cluster_149	Firmicutes	Bacilli	Lactobacillales	Carnobacteriaceae	Alloiococcus	bacterium #1 from	5.43	1.90E-05	0.00095
Cluster_615	Firmicutes	Bacilli	Lactobacillales	Carnobacteriaceae	Lacticigenium	unknown species	4.65	1.21E-02	0.00024
Cluster_28	Firmicutes	Bacilli	Lactobacillales	Carnobacteriaceae	Trichococcus	unknown species	3.70	1.63E-06	0.00421
Cluster_70	Firmicutes	Bacilli	Lactobacillales	Carnobacteriaceae	unknown genus	unknown species	3.12	4.47E-03	0.00275
Cluster_360	Firmicutes	Bacilli	Lactobacillales	Enterococcaceae	Enterococcus	Multi-affiliation	3.87	4.20E-03	0.00058
Cluster_36	Firmicutes	Bacilli	Lactobacillales	Enterococcaceae	Enterococcus	Multi-affiliation	4.91	2.43E-05	0.00409
Cluster_20	Firmicutes	Bacilli	Lactobacillales	Enterococcaceae	Enterococcus	unknown species	3.09	4.74E-04	0.00643
Cluster_134	Firmicutes	Bacilli	Lactobacillales	Lactobacillaceae	Lactobacillus	Multi-affiliation	5.21	4.93E-04	0.00103
Cluster_799	Firmicutes	Bacilli	Lactobacillales	Multi-affiliation	Multi-affiliation	Multi-affiliation	5.66	1.71E-04	0.00017
Cluster_277	Firmicutes	Bacilli	Lactobacillales	Streptococcaceae	Multi-affiliation	Multi-affiliation	5.08	3.32E-02	0.00056
Cluster_993	Firmicutes	Bacilli	Lactobacillales	Streptococcaceae	Streptococcus	Multi-affiliation	4.89	9.38E-04	0.00021
Cluster_26	Firmicutes	Bacilli	Lactobacillales	Streptococcaceae	Streptococcus	Multi-affiliation	3.96	3.07E-03	0.00315
Cluster_113	Firmicutes	Bacilli	Lactobacillales	Streptococcaceae	Streptococcus	Streptococcus henryi	5.07	4.06E-03	0.00076
Cluster_1652	Firmicutes	Clostridia	Clostridiales	Christensenellaceae	Christensenellaceae R-7	unknown species	4.16	4.92E-02	0.00004
Cluster_1365	Firmicutes	Clostridia	Clostridiales	Christensenellaceae	Christensenellaceae R-7	unknown species	5.11	5.35E-03	0.00007
Cluster_1311	Firmicutes	Clostridia	Clostridiales	Christensenellaceae	Christensenellaceae R-7	unknown species	4.14	3.30E-02	0.00009
Cluster_2252	Firmicutes	Clostridia	Clostridiales	Christensenellaceae	Christensenellaceae R-7	unknown species	4.07	3.38E-02	0.00010
Cluster_1964	Firmicutes	Clostridia	Clostridiales	Christensenellaceae	Christensenellaceae R-7	unknown species	5.04	5.18E-04	0.00011
Cluster_635	Firmicutes	Clostridia	Clostridiales	Christensenellaceae	Christensenellaceae R-7	unknown species	4.78	2.77E-04	0.00011
Cluster_1020	Firmicutes	Clostridia	Clostridiales	Christensenellaceae	Christensenellaceae R-7	unknown species	3.80	5.11E-03	0.00012
Cluster_917	Firmicutes	Clostridia	Clostridiales	Christensenellaceae	Christensenellaceae R-7	unknown species	3.97	2.46E-04	0.00016
Cluster_995	Firmicutes	Clostridia	Clostridiales	Christensenellaceae	Christensenellaceae R-7	unknown species	5.52	1.00E-05	0.00018
Cluster_709	Firmicutes	Clostridia	Clostridiales	Christensenellaceae	Christensenellaceae R-7	unknown species	3.01	2.84E-02	0.00019
Cluster_341	Firmicutes	Clostridia	Clostridiales	Christensenellaceae	Christensenellaceae R-7	unknown species	3.83	2.80E-04	0.00029
Cluster_318	Firmicutes	Clostridia	Clostridiales	Christensenellaceae	Christensenellaceae R-7	unknown species	3.51	3.28E-04	0.00041
Cluster_258	Firmicutes	Clostridia	Clostridiales	Christensenellaceae	Christensenellaceae R-7	unknown species	4.07	4.19E-06	0.00048
Cluster_1131	Firmicutes	Clostridia	Clostridiales	Clostridiaceae 1	Clostridium sensu	Multi-affiliation	5.25	7.40E-03	0.00009
Cluster_152	Firmicutes	Clostridia	Clostridiales	Clostridiaceae 1	Multi-affiliation	Multi-affiliation	2.31	1.98E-02	0.00097
Cluster_1774	Firmicutes	Clostridia	Clostridiales	Clostridiales vadinBB60	unknown genus	unknown species	5.59	4.90E-03	0.00012
Cluster_1675	Firmicutes	Clostridia	Clostridiales	Eubacteriaceae	Anaerofustis	unknown species	3.80	3.30E-02	0.00005
Cluster_1134	Firmicutes	Clostridia	Clostridiales	Eubacteriaceae	Eubacterium	unknown species	5.01	1.14E-02	0.00009
Cluster_1007	Firmicutes	Clostridia	Clostridiales	Family XI	Tissierella	unknown species	6.73	1.01E-05	0.00017
Cluster_247	Firmicutes	Clostridia	Clostridiales	Family XI	Tissierella	unknown species	3.72	9.59E-04	0.00067
Cluster_977	Firmicutes	Clostridia	Clostridiales	Family XIII	[Eubacterium] brachy	unknown species	5.63	1.12E-05	0.00010
Cluster_1780	Firmicutes	Clostridia	Clostridiales	Family XIII	[Eubacterium] brachy	unknown species	3.80	2.11E-02	0.00011
Cluster_504	Firmicutes	Clostridia	Clostridiales	Family XIII	[Eubacterium] brachy	unknown species	2.36	2.06E-02	0.00025
Cluster_446	Firmicutes	Clostridia	Clostridiales	Family XIII	[Eubacterium] nodatum	unknown species	5.21	2.30E-06	0.00035
Cluster_421	Firmicutes	Clostridia	Clostridiales	Family XIII	Family XIII AD3011	unknown species	4.38	2.05E-04	0.00025
Cluster_273	Firmicutes	Clostridia	Clostridiales	Family XIII	Family XIII AD3011	unknown species	3.58	1.11E-04	0.00052
Cluster_561	Firmicutes	Clostridia	Clostridiales	Family XIII	Multi-affiliation	Multi-affiliation	4.45	3.85E-04	0.00026
Cluster_202	Firmicutes	Clostridia	Clostridiales	Family XIII	Multi-affiliation	Multi-affiliation	4.19	2.41E-04	0.00058
Cluster_862	Firmicutes	Clostridia	Clostridiales	Lachnospiraceae	[Ruminococcus] gauvreauii group	unknown species	5.59	4.08E-06	0.00022
Cluster_546	Firmicutes	Clostridia	Clostridiales	Lachnospiraceae	[Ruminococcus] gauvreauii group	unknown species	5.15	4.24E-06	0.00026
Cluster_1216	Firmicutes	Clostridia	Clostridiales	Lachnospiraceae	Acetitomaculum	unknown species	4.93	1.41E-02	0.00008
Cluster_2431	Firmicutes	Clostridia	Clostridiales	Lachnospiraceae	Anaerostipes	unknown species	3.99	7.30E-03	0.00006
Cluster_818	Firmicutes	Clostridia	Clostridiales	Lachnospiraceae	Blautia	unknown species	3.06	3.98E-02	0.00013
Cluster_657	Firmicutes	Clostridia	Clostridiales	Lachnospiraceae	Blautia	unknown species	5.67	3.23E-06	0.00014
Cluster_1560	Firmicutes	Clostridia	Clostridiales	Lachnospiraceae	Cellulosilyticum	bacterium PS7	6.41	6.45E-05	0.00011
Cluster_667	Firmicutes	Clostridia	Clostridiales	Lachnospiraceae	Cellulosilyticum	unknown species	4.75	1.85E-03	0.00020

Cluster_281	Firmicutes	Clostridia	Clostridiales	Lachnospiraceae	Coprococcus 1	unknown species	5.79	8.11E-05	0.00032
Cluster_197	Firmicutes	Clostridia	Clostridiales	Lachnospiraceae	Coprococcus 1	unknown species	4.11	7.26E-05	0.00048
Cluster_133	Firmicutes	Clostridia	Clostridiales	Lachnospiraceae	Coprococcus 1	unknown species	6.83	1.78E-12	0.00075
Cluster_1264	Firmicutes	Clostridia	Clostridiales	Lachnospiraceae	Coprococcus 2	unknown species	2.67	2.79E-02	0.00014
Cluster_399	Firmicutes	Clostridia	Clostridiales	Lachnospiraceae	Coprococcus 3	unknown species	3.76	7.43E-04	0.00037
Cluster_548	Firmicutes	Clostridia	Clostridiales	Lachnospiraceae	Coprococcus 3	unknown species	4.27	4.17E-04	0.00066
Cluster_135	Firmicutes	Clostridia	Clostridiales	Lachnospiraceae	Coprococcus 3	unknown species	4.01	2.79E-04	0.00078
Cluster_550	Firmicutes	Clostridia	Clostridiales	Lachnospiraceae	Howardella	unknown species	3.10	3.53E-03	0.00056
Cluster_456	Firmicutes	Clostridia	Clostridiales	Lachnospiraceae	Lachnospiraceae FCS020 group	unknown species	4.18	5.51E-03	0.00030
Cluster_1031	Firmicutes	Clostridia	Clostridiales	Lachnospiraceae	Lachnospiraceae NK3A20 group	unknown species	2.77	1.97E-02	0.00026
Cluster_425	Firmicutes	Clostridia	Clostridiales	Lachnospiraceae	Lachnospiraceae NK3A20 group	unknown species	2.35	2.43E-02	0.00037
Cluster_248	Firmicutes	Clostridia	Clostridiales	Lachnospiraceae	Lachnospiraceae NK3A20 group	unknown species	2.68	1.96E-03	0.00065
Cluster_203	Firmicutes	Clostridia	Clostridiales	Lachnospiraceae	Lachnospiraceae NK3A20 group	unknown species	2.72	2.03E-03	0.00087
Cluster_207	Firmicutes	Clostridia	Clostridiales	Lachnospiraceae	Lachnospiraceae NK3A20 group	unknown species	2.14	7.27E-04	0.00132
Cluster_1045	Firmicutes	Clostridia	Clostridiales	Lachnospiraceae	Lachnospiraceae NK4A136 group	unknown species	4.33	1.41E-02	0.00007
Cluster_483	Firmicutes	Clostridia	Clostridiales	Lachnospiraceae	Lachnospiraceae NK4A136 group	unknown species	4.37	3.26E-02	0.00007
Cluster_1269	Firmicutes	Clostridia	Clostridiales	Lachnospiraceae	Lachnospiraceae NK4A136 group	unknown species	5.81	5.57E-04	0.00010
Cluster_1002	Firmicutes	Clostridia	Clostridiales	Lachnospiraceae	Lachnospiraceae NK4A136 group	unknown species	5.11	4.53E-03	0.00014
Cluster_724	Firmicutes	Clostridia	Clostridiales	Lachnospiraceae	Lachnospiraceae NK4A136 group	unknown species	3.31	3.49E-02	0.00016
Cluster_1279	Firmicutes	Clostridia	Clostridiales	Lachnospiraceae	Lachnospiraceae NK4A136 group	unknown species	6.20	4.24E-06	0.00018
Cluster_1071	Firmicutes	Clostridia	Clostridiales	Lachnospiraceae	Lachnospiraceae NK4A136 group	unknown species	5.80	4.97E-04	0.00019
Cluster_201	Firmicutes	Clostridia	Clostridiales	Lachnospiraceae	Lachnospiraceae NK4A136 group	unknown species	2.64	2.35E-02	0.00060
Cluster_1540	Firmicutes	Clostridia	Clostridiales	Lachnospiraceae	Lachnospiraceae UCG-	unknown species	4.96	2.21E-05	0.00015
Cluster_236	Firmicutes	Clostridia	Clostridiales	Lachnospiraceae	Lachnospiraceae UCG-	unknown species	3.21	5.35E-03	0.00050
Cluster_693	Firmicutes	Clostridia	Clostridiales	Lachnospiraceae	Marvinbryantia	unknown species	4.32	2.39E-02	0.00013
Cluster_1022	Firmicutes	Clostridia	Clostridiales	Lachnospiraceae	Marvinbryantia	unknown species	6.57	8.58E-06	0.00019
Cluster_334	Firmicutes	Clostridia	Clostridiales	Lachnospiraceae	Marvinbryantia	unknown species	4.38	6.91E-06	0.00069
Cluster_1706	Firmicutes	Clostridia	Clostridiales	Lachnospiraceae	Mobilitalea	unknown species	5.24	1.79E-02	0.00005
Cluster_2244	Firmicutes	Clostridia	Clostridiales	Lachnospiraceae	Multi-affiliation	Multi-affiliation	4.79	9.30E-03	0.00006
Cluster_2327	Firmicutes	Clostridia	Clostridiales	Lachnospiraceae	Multi-affiliation	Multi-affiliation	5.59	1.40E-03	0.00013
Cluster_378	Firmicutes	Clostridia	Clostridiales	Lachnospiraceae	Multi-affiliation	Multi-affiliation	4.82	5.50E-04	0.00019
Cluster_357	Firmicutes	Clostridia	Clostridiales	Lachnospiraceae	Multi-affiliation	Multi-affiliation	2.98	9.03E-03	0.00036
Cluster_163	Firmicutes	Clostridia	Clostridiales	Lachnospiraceae	Multi-affiliation	Multi-affiliation	3.77	1.65E-04	0.00048
Cluster_1370	Firmicutes	Clostridia	Clostridiales	Lachnospiraceae	Pseudobutyrivibrio	unknown species	4.06	4.44E-02	0.00009
Cluster_974	Firmicutes	Clostridia	Clostridiales	Lachnospiraceae	Pseudobutyrivibrio	unknown species	4.31	1.09E-02	0.00014
Cluster_93	Firmicutes	Clostridia	Clostridiales	Lachnospiraceae	Roseburia	unknown species	3.57	7.73E-06	0.00124
Cluster_747	Firmicutes	Clostridia	Clostridiales	Lachnospiraceae	Syntrophococcus	unknown species	3.67	2.06E-02	0.00013
Cluster_1272	Firmicutes	Clostridia	Clostridiales	Lachnospiraceae	Tyzzerella 4	unknown species	5.62	5.21E-05	0.00016
Cluster_332	Firmicutes	Clostridia	Clostridiales	Lachnospiraceae	Tyzzerella 4	unknown species	3.14	5.96E-03	0.00041
Cluster_1685	Firmicutes	Clostridia	Clostridiales	Lachnospiraceae	unknown genus	unknown species	3.90	2.39E-02	0.00012
Cluster_547	Firmicutes	Clostridia	Clostridiales	Lachnospiraceae	unknown genus	unknown species	4.76	6.63E-04	0.00015
Cluster_354	Firmicutes	Clostridia	Clostridiales	Lachnospiraceae	unknown genus	unknown species	3.46	2.77E-04	0.00040
Cluster_124	Firmicutes	Clostridia	Clostridiales	Lachnospiraceae	unknown genus	unknown species	4.24	2.68E-04	0.00112
Cluster_2395	Firmicutes	Clostridia	Clostridiales	Multi-affiliation	Multi-affiliation	Multi-affiliation	4.76	4.82E-03	0.00008
Cluster_1093	Firmicutes	Clostridia	Clostridiales	Multi-affiliation	Multi-affiliation	Multi-affiliation	5.15	2.14E-03	0.00011
Cluster_778	Firmicutes	Clostridia	Clostridiales	Multi-affiliation	Multi-affiliation	Multi-affiliation	4.78	6.04E-03	0.00011
Cluster_1230	Firmicutes	Clostridia	Clostridiales	Multi-affiliation	Multi-affiliation	Multi-affiliation	5.27	6.04E-03	0.00012
Cluster_681	Firmicutes	Clostridia	Clostridiales	Multi-affiliation	Multi-affiliation	Multi-affiliation	5.73	1.01E-04	0.00017
Cluster_1408	Firmicutes	Clostridia	Clostridiales	Multi-affiliation	Multi-affiliation	Multi-affiliation	5.12	4.09E-03	0.00018
Cluster_722	Firmicutes	Clostridia	Clostridiales	Multi-affiliation	Multi-affiliation	Multi-affiliation	6.32	4.68E-09	0.00030
Cluster_209	Firmicutes	Clostridia	Clostridiales	Multi-affiliation	Multi-affiliation	Multi-affiliation	4.27	9.95E-07	0.00043
Cluster_166	Firmicutes	Clostridia	Clostridiales	Multi-affiliation	Multi-affiliation	Multi-affiliation	2.52	3.58E-02	0.00067
Cluster_211	Firmicutes	Clostridia	Clostridiales	Multi-affiliation	Multi-affiliation	Multi-affiliation	2.66	4.93E-03	0.00076
Cluster_129	Firmicutes	Clostridia	Clostridiales	Multi-affiliation	Multi-affiliation	Multi-affiliation	3.88	2.83E-04	0.00089
Cluster_44	Firmicutes	Clostridia	Clostridiales	Multi-affiliation	Multi-affiliation	Multi-affiliation	3.56	4.08E-06	0.00333
Cluster_23	Firmicutes	Clostridia	Clostridiales	Multi-affiliation	Multi-affiliation	Multi-affiliation	3.66	1.40E-06	0.00924
Cluster_224	Firmicutes	Clostridia	Clostridiales	Peptostreptococcaceae	Multi-affiliation	Multi-affiliation	3.00	9.32E-03	0.00059
Cluster_142	Firmicutes	Clostridia	Clostridiales	Peptostreptococcaceae	Multi-affiliation	Multi-affiliation	3.19	6.08E-04	0.00114
Cluster_41	Firmicutes	Clostridia	Clostridiales	Peptostreptococcaceae	Multi-affiliation	Multi-affiliation	2.29	1.58E-03	0.00429
Cluster_1106	Firmicutes	Clostridia	Clostridiales	Ruminococcaceae	[Eubacterium] coprostanoligenes	unknown species	2.83	4.73E-02	0.00009
Cluster_338	Firmicutes	Clostridia	Clostridiales	Ruminococcaceae	[Eubacterium] coprostanoligenes	unknown species	3.54	3.42E-02	0.00010
Cluster_597	Firmicutes	Clostridia	Clostridiales	Ruminococcaceae	[Eubacterium] coprostanoligenes	unknown species	3.73	1.85E-03	0.00015
Cluster_1844	Firmicutes	Clostridia	Clostridiales	Ruminococcaceae	[Eubacterium] coprostanoligenes	unknown species	5.54	1.19E-02	0.00016

Cluster_890	Firmicutes	Clostridia	Clostridiales	Ruminococcaceae	[Eubacterium] coprostanoligenes	unknown species	3.97	3.51E-03	0.00016
Cluster_792	Firmicutes	Clostridia	Clostridiales	Ruminococcaceae	[Eubacterium] coprostanoligenes	unknown species	3.20	2.90E-02	0.00019
Cluster_400	Firmicutes	Clostridia	Clostridiales	Ruminococcaceae	[Eubacterium] coprostanoligenes	unknown species	3.55	2.16E-03	0.00028
Cluster_272	Firmicutes	Clostridia	Clostridiales	Ruminococcaceae	[Eubacterium] coprostanoligenes	unknown species	2.43	1.88E-02	0.00031
Cluster_199	Firmicutes	Clostridia	Clostridiales	Ruminococcaceae	[Eubacterium] coprostanoligenes	unknown species	3.89	4.02E-03	0.00061
Cluster_2234	Firmicutes	Clostridia	Clostridiales	Ruminococcaceae	Oscillibacter	unknown species	4.87	2.76E-03	0.00008
Cluster_530	Firmicutes	Clostridia	Clostridiales	Ruminococcaceae	Ruminiclostridium	unknown species	3.44	3.78E-02	0.00035
Cluster_824	Firmicutes	Clostridia	Clostridiales	Ruminococcaceae	Ruminococcaceae NK4A214 group	unknown species	4.32	4.95E-04	0.00025
Cluster_241	Firmicutes	Clostridia	Clostridiales	Ruminococcaceae	Ruminococcaceae NK4A214 group	unknown species	3.05	1.56E-03	0.00074
Cluster_474	Firmicutes	Clostridia	Clostridiales	Ruminococcaceae	Ruminococcaceae UCG-	unknown species	3.32	2.08E-02	0.00018
Cluster_759	Firmicutes	Clostridia	Clostridiales	Ruminococcaceae	Ruminococcaceae UCG-	unknown species	4.63	2.08E-06	0.00023
Cluster_467	Firmicutes	Clostridia	Clostridiales	Ruminococcaceae	Ruminococcaceae UCG-	unknown species	4.04	3.57E-03	0.00008
Cluster_637	Firmicutes	Clostridia	Clostridiales	Ruminococcaceae	Ruminococcaceae UCG-	unknown species	4.51	2.17E-03	0.00008
Cluster_698	Firmicutes	Clostridia	Clostridiales	Ruminococcaceae	Ruminococcaceae UCG-	unknown species	3.93	8.91E-03	0.00013
Cluster_1309	Firmicutes	Clostridia	Clostridiales	Ruminococcaceae	Ruminococcaceae UCG-	unknown species	4.34	4.20E-03	0.00015
Cluster_369	Firmicutes	Clostridia	Clostridiales	Ruminococcaceae	Ruminococcaceae UCG-	unknown species	3.93	2.43E-06	0.00039
Cluster_256	Firmicutes	Clostridia	Clostridiales	Ruminococcaceae	Ruminococcaceae UCG-	unknown species	2.70	1.25E-03	0.00048
Cluster_435	Firmicutes	Clostridia	Clostridiales	Ruminococcaceae	Ruminococcaceae UCG-	unknown species	5.37	2.11E-06	0.00064
Cluster_114	Firmicutes	Clostridia	Clostridiales	Ruminococcaceae	Ruminococcaceae UCG-	unknown species	2.84	6.04E-03	0.00094
Cluster_91	Firmicutes	Clostridia	Clostridiales	Ruminococcaceae	Ruminococcaceae UCG-	unknown species	2.40	3.94E-03	0.00100
Cluster_215	Firmicutes	Clostridia	Clostridiales	Ruminococcaceae	Ruminococcaceae UCG-	unknown species	2.98	6.41E-05	0.00114
Cluster_184	Firmicutes	Clostridia	Clostridiales	Ruminococcaceae	Ruminococcaceae UCG-	unknown species	4.38	4.45E-08	0.00167
Cluster_39	Firmicutes	Clostridia	Clostridiales	Ruminococcaceae	Ruminococcaceae UCG-	unknown species	3.88	7.99E-09	0.00895
Cluster_570	Firmicutes	Clostridia	Clostridiales	Ruminococcaceae	Ruminococcaceae UCG-	unknown species	4.02	1.97E-02	0.00022
Cluster_407	Firmicutes	Clostridia	Clostridiales	Ruminococcaceae	Ruminococcaceae UCG-	unknown species	3.08	2.56E-02	0.00023
Cluster_688	Firmicutes	Clostridia	Clostridiales	Ruminococcaceae	Ruminococcaceae UCG-	unknown species	2.84	1.40E-02	0.00029
Cluster_289	Firmicutes	Clostridia	Clostridiales	Ruminococcaceae	Ruminococcaceae UCG-	unknown species	4.38	9.74E-06	0.00036
Cluster_2630	Firmicutes	Clostridia	Clostridiales	Ruminococcaceae	Ruminococcaceae UCG-	unknown species	4.60	7.62E-03	0.00005
Cluster_568	Firmicutes	Clostridia	Clostridiales	Ruminococcaceae	Ruminococcaceae UCG-	unknown species	3.82	8.93E-03	0.00006
Cluster_1448	Firmicutes	Clostridia	Clostridiales	Ruminococcaceae	Ruminococcaceae UCG-	unknown species	4.69	1.93E-02	0.00006
Cluster_1149	Firmicutes	Clostridia	Clostridiales	Ruminococcaceae	Ruminococcaceae UCG-	unknown species	4.47	8.12E-03	0.00006
Cluster_1222	Firmicutes	Clostridia	Clostridiales	Ruminococcaceae	Ruminococcaceae UCG-	unknown species	4.82	7.34E-03	0.00008
Cluster_1569	Firmicutes	Clostridia	Clostridiales	Ruminococcaceae	Ruminococcaceae UCG-	unknown species	5.12	4.97E-03	0.00008
Cluster_1981	Firmicutes	Clostridia	Clostridiales	Ruminococcaceae	Ruminococcaceae UCG-	unknown species	4.58	2.55E-02	0.00008
Cluster_1363	Firmicutes	Clostridia	Clostridiales	Ruminococcaceae	Ruminococcaceae UCG-	unknown species	4.23	2.80E-02	0.00013
Cluster_620	Firmicutes	Clostridia	Clostridiales	Ruminococcaceae	Ruminococcaceae UCG-	unknown species	6.48	1.40E-06	0.00021
Cluster_912	Firmicutes	Clostridia	Clostridiales	Ruminococcaceae	Ruminococcaceae UCG-	unknown species	2.70	2.96E-02	0.00022
Cluster_1296	Firmicutes	Clostridia	Clostridiales	Ruminococcaceae	Ruminococcaceae UCG-	unknown species	4.88	9.09E-05	0.00035
Cluster_303	Firmicutes	Clostridia	Clostridiales	Ruminococcaceae	Ruminococcaceae UCG-	unknown species	2.57	7.98E-03	0.00061
Cluster_2121	Firmicutes	Clostridia	Clostridiales	Ruminococcaceae	Ruminococcaceae UCG-	unknown species	4.28	4.73E-02	0.00006
Cluster_1761	Firmicutes	Clostridia	Clostridiales	Ruminococcaceae	Ruminococcaceae UCG-	unknown species	4.73	4.34E-03	0.00014
Cluster_764	Firmicutes	Clostridia	Clostridiales	Ruminococcaceae	Ruminococcaceae UCG-	unknown species	4.45	9.03E-03	0.00014
Cluster_2710	Firmicutes	Clostridia	Clostridiales	Ruminococcaceae	Ruminococcaceae UCG-	unknown species	5.58	1.96E-03	0.00015
Cluster_574	Firmicutes	Clostridia	Clostridiales	Ruminococcaceae	Ruminococcaceae UCG-	unknown species	4.75	8.99E-05	0.00019
Cluster_706	Firmicutes	Clostridia	Clostridiales	Ruminococcaceae	Ruminococcaceae UCG-	unknown species	7.09	5.46E-09	0.00025
Cluster_553	Firmicutes	Clostridia	Clostridiales	Ruminococcaceae	Ruminococcaceae UCG-	unknown species	6.65	5.23E-08	0.00030
Cluster_774	Firmicutes	Clostridia	Clostridiales	Ruminococcaceae	Ruminococcaceae UCG-	unknown species	3.27	3.43E-03	0.00034
Cluster_509	Firmicutes	Clostridia	Clostridiales	Ruminococcaceae	Ruminococcaceae UCG-	unknown species	3.54	3.43E-03	0.00041
Cluster_153	Firmicutes	Clostridia	Clostridiales	Ruminococcaceae	Ruminococcaceae UCG-	unknown species	4.02	6.01E-05	0.00103
Cluster_103	Firmicutes	Clostridia	Clostridiales	Ruminococcaceae	Ruminococcaceae UCG-	unknown species	4.45	1.40E-06	0.00117
Cluster_1297	Firmicutes	Clostridia	Clostridiales	Ruminococcaceae	Ruminococcus 1	unknown species	4.79	8.45E-03	0.00009
Cluster_1047	Firmicutes	Clostridia	Clostridiales	Ruminococcaceae	Ruminococcus 2	unknown species	4.19	6.62E-03	0.00011
Cluster_600	Firmicutes	Clostridia	Clostridiales	Ruminococcaceae	Ruminococcus 2	unknown species	4.67	3.28E-04	0.00025
Cluster_214	Firmicutes	Clostridia	Clostridiales	Ruminococcaceae	Ruminococcus 2	unknown species	3.07	5.26E-03	0.00064
Cluster_189	Firmicutes	Clostridia	Clostridiales	Ruminococcaceae	Ruminococcus 2	unknown species	4.67	1.52E-05	0.00080
Cluster_92	Firmicutes	Clostridia	Clostridiales	Ruminococcaceae	Ruminococcus 2	unknown species	2.78	3.43E-03	0.00173
Cluster_1579	Firmicutes	Clostridia	Clostridiales	Ruminococcaceae	Saccharofermentans	Multi-affiliation	3.72	1.56E-02	0.00029
Cluster_1522	Firmicutes	Clostridia	Clostridiales	Ruminococcaceae	unknown genus	unknown species	4.59	1.24E-02	0.00006
Cluster_789	Firmicutes	Clostridia	Clostridiales	Ruminococcaceae	unknown genus	unknown species	4.22	4.82E-03	0.00013
Cluster_1316	Firmicutes	Clostridia	Clostridiales	Ruminococcaceae	unknown genus	unknown species	4.59	7.27E-04	0.00013
Cluster_1388	Firmicutes	Clostridia	Clostridiales	Ruminococcaceae	unknown genus	unknown species	3.82	1.27E-02	0.00015
Cluster_1289	Firmicutes	Clostridia	Clostridiales	Ruminococcaceae	unknown genus	unknown species	6.23	6.20E-05	0.00018
Cluster_1103	Firmicutes	Clostridia	Clostridiales	Ruminococcaceae	unknown genus	unknown species	4.40	4.62E-03	0.00025
Cluster_350	Firmicutes	Clostridia	Clostridiales	Ruminococcaceae	unknown genus	unknown species	2.61	3.30E-02	0.00028
Cluster_755	Firmicutes	Clostridia	Clostridiales	Ruminococcaceae	unknown genus	unknown species	3.08	4.40E-02	0.00028
Cluster_287	Firmicutes	Clostridia	Clostridiales	Ruminococcaceae	unknown genus	unknown species	3.41	2.86E-03	0.00044
Cluster_956	Firmicutes	Erysipelotrichia	Erysipelotrichales	Erysipelotrichaceae	[Anaerorhabdus] furcosa group	unknown species	4.46	3.77E-02	0.00007
Cluster_1544	Firmicutes	Erysipelotrichia	Erysipelotrichales	Erysipelotrichaceae	[Anaerorhabdus] furcosa group	unknown species	4.27	4.06E-03	0.00007
Cluster_336	Firmicutes	Erysipelotrichia	Erysipelotrichales	Erysipelotrichaceae	Erysipelothrix	Multi-affiliation	2.58	2.39E-02	0.00029
Cluster_581	Firmicutes	Erysipelotrichia	Erysipelotrichales	Erysipelotrichaceae	Faecalitalea	unknown species	5.56	1.35E-03	0.00022
Cluster_299	Firmicutes	Erysipelotrichia	Erysipelotrichales	Erysipelotrichaceae	Turicibacter	unknown species	5.10	4.26E-06	0.00055
Cluster_541	Firmicutes	Multi-affiliation	Multi-affiliation	Multi-affiliation	Multi-affiliation	Multi-affiliation	4.51	3.06E-03	0.00022
Cluster_692	Firmicutes	Multi-affiliation	Multi-affiliation	Multi-affiliation	Multi-affiliation	Multi-affiliation	3.39	4.00E-02	0.00034

Cluster_61	Firmicutes	Multi-affiliation	Multi-affiliation	Multi-affiliation	Multi-affiliation	Multi-affiliation	2.82	1.01E-04	0.00241
Cluster_40	Firmicutes	Negativicutes	Selenomonadales	Acidaminococcaceae	Phascolarctobacterium	unknown species	4.59	5.36E-06	0.00391
Cluster_499	Firmicutes	Negativicutes	Selenomonadales	Veillonellaceae	Anaerovibrio	unknown species	4.79	1.55E-04	0.00035
Cluster_729	Hydrogenedente	unknown class	unknown order	unknown family	unknown genus	unknown species	4.24	1.59E-02	0.00025
Cluster_221	Multi-affiliation	Multi-affiliation	Multi-affiliation	Multi-affiliation	Multi-affiliation	Multi-affiliation	5.01	9.20E-06	0.00065
Cluster_46	Multi-affiliation	Multi-affiliation	Multi-affiliation	Multi-affiliation	Multi-affiliation	Multi-affiliation	4.05	6.01E-09	0.00177
Cluster_1120	Parcubacteria	unknown class	unknown order	unknown family	unknown genus	unknown species	4.04	9.03E-03	0.00006
Cluster_1490	Parcubacteria	unknown class	unknown order	unknown family	unknown genus	unknown species	3.90	2.09E-02	0.00010
Cluster_1287	Parcubacteria	unknown class	unknown order	unknown family	unknown genus	unknown species	3.74	6.03E-03	0.00016
Cluster_630	Parcubacteria	unknown class	unknown order	unknown family	unknown genus	unknown species	-2.58	4.48E-02	0.00017
Cluster_743	Parcubacteria	unknown class	unknown order	unknown family	unknown genus	unknown species	-2.19	3.79E-02	0.00039
Cluster_140	Planctomycetes	Planctomycetac	Planctomycetales	Planctomycetaceae	p-1088-a5 gut group	unknown species	5.91	9.21E-09	0.00053
Cluster_590	Planctomycetes	Planctomycetac	Planctomycetales	Planctomycetaceae	Rhodopirellula	unknown species	5.13	1.27E-03	0.00034
Cluster_1015	Proteobacteria	Alphaproteobac	Caulobacterales	Caulobacteraceae	Brevundimonas	Multi-affiliation	2.85	1.79E-02	0.00018
Cluster_358	Proteobacteria	Alphaproteobac	Rhizobiales	Hyphomicrobiaceae	Devosia	Multi-affiliation	3.00	2.31E-03	0.00049
Cluster_498	Proteobacteria	Alphaproteobac	Rhizobiales	Hyphomicrobiaceae	Devosia	unknown species	4.02	1.13E-04	0.00029
Cluster_708	Proteobacteria	Alphaproteobac	Rhizobiales	Hyphomicrobiaceae	Devosia	unknown species	4.96	2.20E-04	0.00031
Cluster_1559	Proteobacteria	Alphaproteobac	Rhizobiales	Phyllobacteriaceae	Aminobacter	Multi-affiliation	5.35	1.67E-02	0.00007
Cluster_758	Proteobacteria	Alphaproteobac	Rhizobiales	Phyllobacteriaceae	Multi-affiliation	Multi-affiliation	5.47	2.29E-04	0.00022
Cluster_116	Proteobacteria	Alphaproteobac	Rhizobiales	Rhizobiaceae	Rhizobium	unknown species	3.00	2.44E-03	0.00138
Cluster_1654	Proteobacteria	Alphaproteobac	Rhodobacterales	Rhodobacteraceae	Defluviimonas	unknown species	3.95	4.76E-02	0.00006
Cluster_160	Proteobacteria	Alphaproteobac	Rhodobacterales	Rhodobacteraceae	Defluviimonas	unknown species	2.12	3.55E-02	0.00126
Cluster_849	Proteobacteria	Alphaproteobac	Rhodobacterales	Rhodobacteraceae	Ketogulonicigenium	Multi-affiliation	3.78	5.57E-04	0.00040
Cluster_1509	Proteobacteria	Alphaproteobac	Rhodobacterales	Rhodobacteraceae	Rhodobacter	unknown species	4.13	9.58E-03	0.00017
Cluster_1215	Proteobacteria	Alphaproteobac	Rhodobacterales	Rhodobacteraceae	unknown genus	unknown species	3.40	3.86E-03	0.00024
Cluster_2129	Proteobacteria	Alphaproteobac	Rickettsiales	Rickettsiaceae	unknown genus	unknown species	3.58	1.60E-02	0.00010
Cluster_1064	Proteobacteria	Alphaproteobac	Sphingomonadales	Sphingomonadaceae	Sphingomonas	Multi-affiliation	3.15	3.67E-02	0.00015
Cluster_1498	Proteobacteria	Betaproteobact	Burkholderiales	Alcaligenaceae	Oligella	unknown species	3.74	2.74E-02	0.00010
Cluster_115	Proteobacteria	Betaproteobact	Burkholderiales	Comamonadaceae	Comamonas	Multi-affiliation	2.53	4.06E-03	0.00257
Cluster_104	Proteobacteria	Betaproteobact	Burkholderiales	Comamonadaceae	Comamonas	unknown species	3.01	3.43E-03	0.00102
Cluster_542	Proteobacteria	Betaproteobact	Burkholderiales	Comamonadaceae	Hydrogenophaga	Hydrogenophaga sp.	3.93	2.91E-02	0.00009
Cluster_710	Proteobacteria	Betaproteobact	Burkholderiales	Comamonadaceae	Hydrogenophaga	Multi-affiliation	5.30	5.75E-04	0.00025
Cluster_1182	Proteobacteria	Betaproteobact	Burkholderiales	Multi-affiliation	Multi-affiliation	Multi-affiliation	4.12	4.10E-02	0.00007
Cluster_684	Proteobacteria	Deltaproteobact	Desulfovibrionales	Desulfovibrionaceae	Desulfovibrio	unknown species	6.28	1.17E-06	0.00016
Cluster_756	Proteobacteria	Deltaproteobact	Desulfuromonadales	GR-WP33-58	unknown genus	unknown species	3.38	2.79E-02	0.00013
Cluster_311	Proteobacteria	Epsilonproteoba	Campylobacterales	Helicobacteraceae	Sulfuricurvum	Multi-affiliation	-3.70	7.26E-03	0.00029
Cluster_420	Proteobacteria	Gammaproteob	Aeromonadales	Succinivibrionaceae	Ruminobacter	unknown species	3.31	1.73E-02	0.00022
Cluster_1023	Proteobacteria	Gammaproteob	Cellvibrionales	Cellvibrionaceae	Cellvibrio	unknown species	4.53	1.31E-02	0.00015
Cluster_1129	Proteobacteria	Gammaproteob	Cellvibrionales	Spongiibacteraceae	BD1-7 clade	unknown species	5.64	2.68E-05	0.00010
Cluster_48	Proteobacteria	Gammaproteob	Oceanospirillales	Halomonadaceae	Halomonas	unknown species	2.72	5.96E-03	0.00235
Cluster_1211	Proteobacteria	Gammaproteob	Pasteurellales	Pasteurellaceae	Pasteurella	Pasteurella testudinis	4.39	4.17E-02	0.00016
Cluster_227	Proteobacteria	Gammaproteob	Pseudomonadales	Moraxellaceae	Acinetobacter	Acinetobacter sp.	2.83	1.64E-02	0.00066
Cluster_310	Proteobacteria	Gammaproteob	Pseudomonadales	Moraxellaceae	Acinetobacter	Multi-affiliation	3.27	4.62E-03	0.00057
Cluster_278	Proteobacteria	Gammaproteob	Pseudomonadales	Moraxellaceae	Acinetobacter	Multi-affiliation	3.24	3.85E-04	0.00077
Cluster_180	Proteobacteria	Gammaproteob	Pseudomonadales	Moraxellaceae	Acinetobacter	Multi-affiliation	2.03	2.75E-02	0.00083
Cluster_151	Proteobacteria	Gammaproteob	Pseudomonadales	Moraxellaceae	Acinetobacter	Multi-affiliation	2.78	5.19E-03	0.00128
Cluster_127	Proteobacteria	Gammaproteob	Pseudomonadales	Moraxellaceae	Acinetobacter	Multi-affiliation	3.39	5.32E-04	0.00134
Cluster_98	Proteobacteria	Gammaproteob	Pseudomonadales	Moraxellaceae	Acinetobacter	Multi-affiliation	2.18	1.91E-02	0.00154
Cluster_60	Proteobacteria	Gammaproteob	Pseudomonadales	Moraxellaceae	Acinetobacter	Multi-affiliation	4.14	7.61E-06	0.00184
Cluster_17	Proteobacteria	Gammaproteob	Pseudomonadales	Moraxellaceae	Acinetobacter	Multi-affiliation	2.95	5.30E-04	0.00578
Cluster_587	Proteobacteria	Gammaproteob	Pseudomonadales	Moraxellaceae	Acinetobacter	unknown species	2.86	4.80E-02	0.00039
Cluster_234	Proteobacteria	Gammaproteob	Pseudomonadales	Moraxellaceae	Acinetobacter	unknown species	3.00	3.81E-02	0.00084
Cluster_485	Proteobacteria	Gammaproteob	Pseudomonadales	Pseudomonadaceae	Pseudomonas	Multi-affiliation	3.83	1.45E-02	0.00045
Cluster_125	Proteobacteria	Gammaproteob	Pseudomonadales	Pseudomonadaceae	Pseudomonas	Multi-affiliation	2.85	6.49E-03	0.00075
Cluster_445	Proteobacteria	Gammaproteob	Xanthomonadales	Xanthomonadaceae	Pseudoxanthomonas	Multi-affiliation	3.16	2.85E-02	0.00021
Cluster_11	Proteobacteria	Gammaproteob	Xanthomonadales	Xanthomonadaceae	Rhodanobacter	Multi-affiliation	2.44	5.66E-04	0.01377
Cluster_1026	Proteobacteria	Gammaproteob	Xanthomonadales	Xanthomonadaceae	Stenotrophomonas	Multi-affiliation	5.22	1.58E-03	0.00033
Cluster_148	Proteobacteria	Gammaproteob	Xanthomonadales	Xanthomonadaceae	Stenotrophomonas	unknown species	2.78	3.03E-03	0.00212
Cluster_1961	Saccharibacteria	unknown class	unknown order	unknown family	Candidatus	unknown species	4.00	2.68E-02	0.00008
Cluster_1334	Saccharibacteria	unknown class	unknown order	unknown family	Candidatus	unknown species	4.06	2.16E-02	0.00011
Cluster_1381	Saccharibacteria	unknown class	unknown order	unknown family	Candidatus	unknown species	6.28	2.11E-04	0.00011
Cluster_1286	Saccharibacteria	unknown class	unknown order	unknown family	Candidatus	unknown species	5.06	1.95E-04	0.00011
Cluster_720	Saccharibacteria	unknown class	unknown order	unknown family	Candidatus	unknown species	6.53	4.40E-05	0.00027
Cluster_293	Saccharibacteria	unknown class	unknown order	unknown family	Candidatus	unknown species	2.48	1.79E-02	0.00051
Cluster_231	Saccharibacteria	unknown class	unknown order	unknown family	Candidatus	unknown species	4.19	1.57E-03	0.00051
Cluster_222	Saccharibacteria	unknown class	unknown order	unknown family	Candidatus	unknown species	5.14	2.19E-06	0.00075
Cluster_47	Saccharibacteria	unknown class	unknown order	unknown family	Candidatus	unknown species	4.14	6.06E-06	0.00373
Cluster_1037	Saccharibacteria	unknown class	unknown order	unknown family	unknown genus	unknown species	5.23	1.30E-02	0.00010
Cluster_1336	SHA-109	unknown class	unknown order	unknown family	unknown genus	unknown species	5.22	1.58E-03	0.00015
Cluster_1083	SHA-109	unknown class	unknown order	unknown family	unknown genus	unknown species	5.16	2.16E-02	0.00020
Cluster_2491	Tenericutes	Mollicutes	Mollicutes RF9	unknown family	unknown genus	unknown species	5.35	2.95E-04	0.00010
Cluster_330	Verrucomicrobia	Verrucomicrobia	Verrucomicrobiales	Verrucomicrobiaceae	Akkermansia	unknown species	6.01	1.48E-04	0.00017
Cluster_1411	Verrucomicrobia	Verrucomicrobia	Verrucomicrobiales	Verrucomicrobiaceae	Verrucomicrobium	unknown species	4.41	1.13E-03	0.00011
Cluster_734	Euryarchaeota	Methanobacteri	Methanobacteriales	Methanobacteriaceae	Methanobrevibacter	unknown species	4.10	9.02E-04	0.00013
Cluster_944	Euryarchaeota	Methanobacteri	Methanobacteriales	Methanobacteriaceae	Methanobrevibacter	Multi-affiliation	3.78	7.34E-03	0.00014
Cluster_563	Euryarchaeota	Methanobacteri	Methanobacteriales	Methanobacteriaceae	Methanobrevibacter	Multi-affiliation	3.95	3.05E-05	0.00040

Table S5. Differentially abundant taxonomic units between D0 and D3 for quarters assigned to Cluster C2, as determined by the LEfSe pipeline. Average abundances (expressed as percentages) are presented for each day. LefSe results include the LDA score and p-val. Most discriminant taxa (with a LDA score > 4) are in bold.

	discriminant taxa						more abundant	LDA		mean abundance	
kingdom	phylum	class	order	family	genus	species	at Day	score	pvalue	D0_C2	D3_C2
**Bacteria**	**Actinobacteria**						**D0**	**4.80**	**0.0217**	**21.045%**	**14.944%**
**Bacteria**	**Actinobacteria**	**Actinobacteria**					**D0**	**4.80**	**0.0217**	**20.849%**	**14.760%**
**Bacteria**	**Actinobacteria**	**Actinobacteria**	**Bifidobacteriales**				**D0**	**4.49**	**0.0008**	**3.012%**	**0.628%**
**Bacteria**	**Actinobacteria**	**Actinobacteria**	**Bifidobacteriales**	**Bifidobacteriaceae**			**D0**	**4.49**	**0.0008**	**3.012%**	**0.628%**
Bacteria	Actinobacteria	Actinobacteria	Bifidobacteriales	Bifidobacteriaceae	Aeriscardovia		D0	2.84	0.0006	0.026%	0.000%
Bacteria	Actinobacteria	Actinobacteria	Bifidobacteriales	Bifidobacteriaceae	Aeriscardovia	unknownspec	D0	2.84	0.0006	0.026%	0.000%
**Bacteria**	**Actinobacteria**	**Actinobacteria**	**Bifidobacteriales**	**Bifidobacteriaceae**	**Bifidobacterium**		**D0**	**4.49**	**0.0009**	**2.914%**	**0.579%**
**Bacteria**	**Actinobacteria**	**Actinobacteria**	**Bifidobacteriales**	**Bifidobacteriaceae**	**Bifidobacterium**	**Multi_affiliat**	**D0**	**4.38**	**0.0003**	**2.226%**	**0.325%**
Bacteria	Actinobacteria	Actinobacteria	Corynebacteriales	Corynebacteriaceae	Corynebacterium1	unknownspec	D0	3.10	0.0037	3.097%	1.456%
Bacteria	Actinobacteria	Actinobacteria	Corynebacteriales	Corynebacteriaceae	Multi_affiliation		D0	3.33	0.0397	0.281%	0.116%
Bacteria	Actinobacteria	Actinobacteria	Corynebacteriales	Corynebacteriaceae	Multi_affiliation	Multi_affiliati	D0	3.33	0.0397	0.281%	0.116%
**Bacteria**	**Actinobacteria**	**Actinobacteria**	**Micrococcales**				**D0**	**4.07**	**0.0343**	**4.663%**	**4.625%**
Bacteria	Actinobacteria	Actinobacteria	Micrococcales	Cellulomonadaceae	Multi_affiliation		D0	2.88	0.0343	0.007%	0.000%
Bacteria	Actinobacteria	Actinobacteria	Micrococcales	Cellulomonadaceae	Multi_affiliation	Multi_affiliati	D0	2.89	0.0343	0.007%	0.000%
Bacteria	Actinobacteria	Actinobacteria	Micrococcales	Dermabacteraceae			D0	3.59	0.0217	0.395%	0.129%
Bacteria	Actinobacteria	Actinobacteria	Micrococcales	Dermabacteraceae	Brachybacterium		D0	3.59	0.0217	0.395%	0.129%
Bacteria	Actinobacteria	Actinobacteria	Micrococcales	Dermabacteraceae	Brachybacterium	Multi_affiliati	D0	3.59	0.0217	0.395%	0.129%
Bacteria	Actinobacteria	Actinobacteria	Micrococcales	Jonesiaceae			D0	2.79	0.0024	0.022%	0.002%
Bacteria	Actinobacteria	Actinobacteria	Micrococcales	Jonesiaceae	Jonesia		D0	2.78	0.0024	0.022%	0.002%
Bacteria	Actinobacteria	Actinobacteria	Micrococcales	Jonesiaceae	Jonesia	Jonesiadenitri	D0	2.79	0.0024	0.022%	0.002%
Bacteria	Actinobacteria	Actinobacteria	Micrococcales	Microbacteriaceae	Leucobacter	unknownspec	D0	2.59	0.0310	0.035%	0.025%
Bacteria	Actinobacteria	Actinobacteria	Micrococcales	Micrococcaceae			D0	3.99	0.0026	1.495%	0.578%
**Bacteria**	**Actinobacteria**	**Actinobacteria**	**Micrococcales**	**Micrococcaceae**	**Arthrobacter**		**D0**	**4.03**	**0.0003**	**1.063%**	**0.332%**
**Bacteria**	**Actinobacteria**	**Actinobacteria**	**Micrococcales**	**Micrococcaceae**	**Arthrobacter**	**Multi_affiliat**	**D0**	**4.03**	**0.0003**	**1.056%**	**0.332%**
Bacteria	Actinobacteria	Actinobacteria	Micrococcales	Micrococcaceae	Multi_affiliation		D0	2.81	0.0030	0.202%	0.040%
Bacteria	Actinobacteria	Actinobacteria	Micrococcales	Micrococcaceae	Multi_affiliation	Multi_affiliati	D0	2.81	0.0030	0.202%	0.040%
Bacteria	Actinobacteria	Actinobacteria	Micrococcales	Micrococcaceae	unknowngenus		D0	2.95	0.0110	0.050%	0.013%
Bacteria	Actinobacteria	Actinobacteria	Micrococcales	Micrococcaceae	unknowngenus	unknownspec	D0	2.96	0.0110	0.117%	0.162%
Bacteria	Actinobacteria	Actinobacteria	Propionibacteriales				D0	3.40	0.0396	0.485%	0.191%
Bacteria	Actinobacteria	Actinobacteria	Propionibacteriales	Nocardioidaceae			D0	3.38	0.0341	0.471%	0.187%
Bacteria	Actinobacteria	Actinobacteria	Propionibacteriales	Nocardioidaceae	Aeromicrobium		D0	2.69	0.0013	0.117%	0.025%
Bacteria	Actinobacteria	Actinobacteria	Propionibacteriales	Nocardioidaceae	Aeromicrobium	Multi_affiliati	D0	2.69	0.0013	0.117%	0.025%
Bacteria	Actinobacteria	Actinobacteria	Propionibacteriales	Nocardioidaceae	Nocardioides		D0	3.30	0.0456	0.337%	0.155%
Bacteria	Actinobacteria	Actinobacteria	Propionibacteriales	Nocardioidaceae	Nocardioides	Multi_affiliati	D0	2.84	0.0027	0.134%	0.034%
Bacteria	Actinobacteria	Actinobacteria	Propionibacteriales	Nocardioidaceae	Nocardioides	unknownspec	D0	3.21	0.0292	0.175%	0.089%
Bacteria	Actinobacteria	Actinobacteria	RL185_aaj71c12	unknownfamily	unknowngenus	unknownspec	D0	2.95	0.0483	0.035%	0.017%
Bacteria	Bacteroidetes	Bacteroidia	Bacteroidales	p_2534_18B5gutgroup			D0	2.70	0.0416	0.048%	0.067%
Bacteria	Bacteroidetes	Bacteroidia	Bacteroidales	p_2534_18B5gutgroup	unknowngenus		D0	2.70	0.0416	0.048%	0.067%
Bacteria	Bacteroidetes	Bacteroidia	Bacteroidales	p_2534_18B5gutgroup	unknowngenus	unknownspec	D0	2.70	0.0416	0.048%	0.067%
Bacteria	Bacteroidetes	Bacteroidia	Bacteroidales	Prevotellaceae	Multi_affiliation		D0	2.70	0.0194	0.021%	0.000%
Bacteria	Bacteroidetes	Bacteroidia	Bacteroidales	Prevotellaceae	Multi_affiliation	Multi_affiliati	D0	2.70	0.0194	0.021%	0.000%
Bacteria	Bacteroidetes	Bacteroidia	Bacteroidales	Prevotellaceae	Prevotella1		D0	2.90	0.0133	0.190%	0.057%
Bacteria	Bacteroidetes	Bacteroidia	Bacteroidales	Prevotellaceae	Prevotella1	unknownspec	D0	2.90	0.0133	0.190%	0.057%
Bacteria	Bacteroidetes	Bacteroidia	Bacteroidales	Prevotellaceae	PrevotellaceaeUCG_003		D0	2.89	0.0321	0.231%	0.201%
Bacteria	Bacteroidetes	Bacteroidia	Bacteroidales	Prevotellaceae	PrevotellaceaeUCG_	unknownspec	D0	2.88	0.0321	0.231%	0.201%
Bacteria	Bacteroidetes	Cytophagia	Cytophagales	Cytophagaceae	Pseudarcicella		D0	2.82	0.0431	0.033%	0.002%
Bacteria	Bacteroidetes	Cytophagia	Cytophagales	Cytophagaceae	Pseudarcicella	unknownspec	D0	2.82	0.0431	0.033%	0.002%
Bacteria	Bacteroidetes	Cytophagia	OrderIII				D0	3.00	0.0406	0.071%	0.005%
Bacteria	Bacteroidetes	Cytophagia	OrderIII	unknownfamily			D0	3.00	0.0406	0.071%	0.005%
Bacteria	Bacteroidetes	Cytophagia	OrderIII	unknownfamily	unknowngenus		D0	3.00	0.0406	0.071%	0.005%
Bacteria	Bacteroidetes	Cytophagia	OrderIII	unknownfamily	unknowngenus	unknownspec	D0	3.00	0.0406	0.071%	0.005%
Bacteria	Bacteroidetes	Sphingobacteriia	Sphingobacteriales	Sphingobacteriaceae	Sphingobacterium	Sphingobacte	D0	3.14	0.0052	0.129%	0.023%
Bacteria	Cyanobacteria	Chloroplast					D0	3.09	0.0211	0.723%	0.705%
Bacteria	Cyanobacteria	Chloroplast	unknownorder				D0	3.09	0.0211	0.547%	0.526%
Bacteria	Cyanobacteria	Chloroplast	unknownorder	unknownfamily			D0	3.09	0.0211	0.547%	0.526%
Bacteria	Cyanobacteria	Chloroplast	unknownorder	unknownfamily	unknowngenus		D0	3.09	0.0211	0.547%	0.526%
Bacteria	Cyanobacteria	Chloroplast	unknownorder	unknownfamily	unknowngenus	Abiesfabri	D0	3.11	0.0103	0.547%	0.526%
Bacteria	Cyanobacteria	ML635J_21					D0	2.96	0.0483	0.068%	0.021%
Bacteria	Cyanobacteria	ML635J_21	unknownorder				D0	2.96	0.0483	0.068%	0.021%
Bacteria	Cyanobacteria	ML635J_21	unknownorder	unknownfamily			D0	2.96	0.0483	0.068%	0.021%
Bacteria	Cyanobacteria	ML635J_21	unknownorder	unknownfamily	unknowngenus		D0	2.97	0.0483	0.068%	0.021%
Bacteria	Cyanobacteria	ML635J_21	unknownorder	unknownfamily	unknowngenus	unknownspec	D0	2.96	0.0483	0.068%	0.021%
Bacteria	Firmicutes	Bacilli	Bacillales	Bacillaceae			D0	3.37	0.0077	0.302%	0.097%
Bacteria	Firmicutes	Bacilli	Bacillales	Bacillaceae	Oceanobacillus		D0	2.81	0.0357	0.074%	0.074%
Bacteria	Firmicutes	Bacilli	Bacillales	Bacillaceae	Oceanobacillus	unknownspec	D0	2.94	0.0013	0.050%	0.000%
Bacteria	Firmicutes	Bacilli	Bacillales	Bacillaceae	Paucisalibacillus		D0	2.86	0.0308	0.043%	0.001%
Bacteria	Firmicutes	Bacilli	Bacillales	Bacillaceae	Paucisalibacillus	unknownspec	D0	2.86	0.0308	0.043%	0.001%
Bacteria	Firmicutes	Bacilli	Bacillales	Bacillaceae	unknowngenus		D0	3.05	0.0055	0.073%	0.000%
Bacteria	Firmicutes	Bacilli	Bacillales	Bacillaceae	unknowngenus	unknownspec	D0	3.05	0.0055	0.073%	0.000%
Bacteria	Firmicutes	Bacilli	Bacillales	Staphylococcaceae	Jeotgalicoccus		D0	3.46	0.0157	0.488%	0.242%
Bacteria	Firmicutes	Bacilli	Bacillales	Staphylococcaceae	Jeotgalicoccus	Multi_affiliati	D0	3.46	0.0157	0.488%	0.242%
Bacteria	Firmicutes	Bacilli	Lactobacillales	Aerococcaceae	Aerococcus	Aerococcusva	D0	3.11	0.0093	0.079%	0.008%
Bacteria	Firmicutes	Bacilli	Lactobacillales	Carnobacteriaceae			D0	3.76	0.0095	1.624%	0.517%
Bacteria	Firmicutes	Bacilli	Lactobacillales	Carnobacteriaceae	Alloiococcus		D0	2.73	0.0216	0.216%	0.119%
Bacteria	Firmicutes	Bacilli	Lactobacillales	Carnobacteriaceae	Alloiococcus	bacterium1frc	D0	2.73	0.0216	0.216%	0.119%
Bacteria	Firmicutes	Bacilli	Lactobacillales	Carnobacteriaceae	unknowngenus		D0	3.60	0.0093	0.434%	0.089%
Bacteria	Firmicutes	Bacilli	Lactobacillales	Carnobacteriaceae	unknowngenus	unknownspec	D0	3.60	0.0093	0.434%	0.089%
Bacteria	Firmicutes	Bacilli	Lactobacillales	Enterococcaceae	Enterococcus	Multi_affiliati	D0	2.98	0.0305	0.856%	0.455%
Bacteria	Firmicutes	Clostridia	Clostridiales	Clostridiaceae1	Fonticella		D0	2.82	0.0411	0.235%	0.271%
Bacteria	Firmicutes	Clostridia	Clostridiales	Clostridiaceae1	Fonticella	unknownspec	D0	2.82	0.0411	0.050%	0.016%
Bacteria	Firmicutes	Clostridia	Clostridiales	Lachnospiraceae	Cellulosilyticum	unknownspec	D0	2.96	0.0335	0.143%	0.036%
Bacteria	Firmicutes	Clostridia	Clostridiales	Lachnospiraceae	Coprococcus1		D0	2.75	0.0416	0.290%	0.216%
Bacteria	Firmicutes	Clostridia	Clostridiales	Lachnospiraceae	Coprococcus1	unknownspec	D0	2.75	0.0416	0.290%	0.216%
Bacteria	Firmicutes	Clostridia	Clostridiales	Peptostreptococcaceae			D0	3.72	0.0021	0.749%	0.286%
Bacteria	Firmicutes	Clostridia	Clostridiales	Peptostreptococcaceae	Multi_affiliation		D0	3.72	0.0021	0.749%	0.286%
Bacteria	Firmicutes	Clostridia	Clostridiales	Peptostreptococcaceae	Multi_affiliation	Multi_affiliati	D0	3.72	0.0021	0.749%	0.286%
Bacteria	Firmicutes	Clostridia	Clostridiales	Ruminococcaceae	unknowngenus		D0	2.84	0.0482	0.341%	0.253%
Bacteria	Firmicutes	Clostridia	Clostridiales	Ruminococcaceae	unknowngenus	unknownspec	D0	2.85	0.0482	0.341%	0.253%
Bacteria	Firmicutes	Erysipelotrichia					D0	3.25	0.0339	0.256%	0.106%

Bacteria	Firmicutes	Erysipelotrichia	Erysipelotrichales				D0	3.25	0.0339	0.256%	0.106%
Bacteria	Firmicutes	Erysipelotrichia	Erysipelotrichales	Erysipelotrichaceae			D0	3.25	0.0339	0.256%	0.106%
Bacteria	Firmicutes	Erysipelotrichia	Erysipelotrichales	Erysipelotrichaceae	Turicibacter		D0	3.12	0.0139	0.104%	0.020%
Bacteria	Firmicutes	Erysipelotrichia	Erysipelotrichales	Erysipelotrichaceae	Turicibacter	unknownspec	D0	3.12	0.0139	0.104%	0.020%
Bacteria	Hydrogenedentes						D0	2.72	0.0194	0.024%	0.042%
Bacteria	Hydrogenedentes	unknownclass					D0	2.72	0.0194	0.024%	0.042%
Bacteria	Hydrogenedentes	unknownclass	unknownorder				D0	2.72	0.0194	0.024%	0.042%
Bacteria	Hydrogenedentes	unknownclass	unknownorder	unknownfamily			D0	2.72	0.0194	0.024%	0.042%
Bacteria	Hydrogenedentes	unknownclass	unknownorder	unknownfamily	unknowngenus		D0	2.72	0.0194	0.024%	0.042%
Bacteria	Hydrogenedentes	unknownclass	unknownorder	unknownfamily	unknowngenus	unknownspec	D0	2.72	0.0194	0.024%	0.042%
Bacteria	Proteobacteria	Alphaproteobacteri	Rhizobiales	Hyphomicrobiaceae			D0	3.27	0.0001	0.213%	0.086%
Bacteria	Proteobacteria	Alphaproteobacteri	Rhizobiales	Hyphomicrobiaceae	Devosia		D0	3.23	0.0001	0.196%	0.028%
Bacteria	Proteobacteria	Alphaproteobacteri	Rhizobiales	Hyphomicrobiaceae	Devosia	Multi_affiliati	D0	3.00	0.0001	0.058%	0.002%
Bacteria	Proteobacteria	Alphaproteobacteri	Rhizobiales	Hyphomicrobiaceae	Devosia	unknownspec	D0	2.98	0.0008	0.138%	0.026%
Bacteria	Proteobacteria	Alphaproteobacteri	Rhizobiales	Hyphomicrobiaceae	Hyphomicrobium		D0	2.76	0.0257	0.017%	0.058%
Bacteria	Proteobacteria	Alphaproteobacteri	Rhizobiales	Hyphomicrobiaceae	Hyphomicrobium	unknownspec	D0	2.79	0.0055	0.017%	0.057%
Bacteria	Proteobacteria	Alphaproteobacteri	Rhizobiales	Phyllobacteriaceae	Aquamicrobium		D0	2.65	0.0060	0.063%	0.026%
Bacteria	Proteobacteria	Alphaproteobacteri	Rhizobiales	Phyllobacteriaceae	Aquamicrobium	Multi_affiliati	D0	2.64	0.0060	0.063%	0.026%
Bacteria	Proteobacteria	Alphaproteobacteri	Rhodobacterales				D0	3.05	0.0252	0.495%	0.245%
Bacteria	Proteobacteria	Alphaproteobacteri	Rhodobacterales	Rhodobacteraceae			D0	3.05	0.0252	0.495%	0.245%
Bacteria	Proteobacteria	Alphaproteobacteri	Rhodobacterales	Rhodobacteraceae	Defluviimonas		D0	2.65	0.0181	0.226%	0.066%
Bacteria	Proteobacteria	Alphaproteobacteri	Rhodobacterales	Rhodobacteraceae	Defluviimonas	unknownspec	D0	2.65	0.0181	0.226%	0.066%
Bacteria	Proteobacteria	Alphaproteobacteri	Rhodobacterales	Rhodobacteraceae	Ketogulonicigenium		D0	2.76	0.0064	0.048%	0.011%
Bacteria	Proteobacteria	Alphaproteobacteri	Rhodobacterales	Rhodobacteraceae	Ketogulonicigenium	Multi_affiliati	D0	2.76	0.0064	0.048%	0.011%
Bacteria	Proteobacteria	Alphaproteobacteri	Rhodobacterales	Rhodobacteraceae	Paracocccus		D0	2.89	0.0012	0.039%	0.014%
Bacteria	Proteobacteria	Alphaproteobacteri	Rhodobacterales	Rhodobacteraceae	Paracocccus	Multi_affiliati	D0	2.89	0.0012	0.039%	0.014%
Bacteria	Proteobacteria	Alphaproteobacteri	Sphingomonadales	Erythrobacteraceae			D0	2.76	0.0027	0.029%	0.000%
Bacteria	Proteobacteria	Alphaproteobacteri	Sphingomonadales	Erythrobacteraceae	Altererythrobacter		D0	2.76	0.0027	0.029%	0.000%
Bacteria	Proteobacteria	Alphaproteobacteri	Sphingomonadales	Erythrobacteraceae	Altererythrobacter	bacteriumenc	D0	2.76	0.0027	0.029%	0.000%
Bacteria	Proteobacteria	Betaproteobacteria	Burkholderiales	Alcaligenaceae	Oligella		D0	2.76	0.0015	0.045%	0.045%
Bacteria	Proteobacteria	Betaproteobacteria	Burkholderiales	Alcaligenaceae	Oligella	unknownspec	D0	2.62	0.0194	0.037%	0.043%
Bacteria	Proteobacteria	Betaproteobacteria	Burkholderiales	Comamonadaceae	Hydrogenophaga	Multi_affiliati	D0	2.69	0.0431	0.028%	0.003%
Bacteria	Proteobacteria	Deltaproteobacteria	Desulfuromonadales				D0	2.63	0.0027	0.042%	0.016%
Bacteria	Proteobacteria	Deltaproteobacteria	Desulfuromonadales	GR_WP33_58			D0	2.63	0.0027	0.042%	0.016%
Bacteria	Proteobacteria	Deltaproteobacteria	Desulfuromonadales	GR_WP33_58	unknowngenus		D0	2.63	0.0027	0.042%	0.016%
Bacteria	Proteobacteria	Deltaproteobacteria	Desulfuromonadales	GR_WP33_58	unknowngenus	unknownspec	D0	2.63	0.0027	0.042%	0.016%
Bacteria	Proteobacteria	Gammaproteobacte	Cellvibrionales	Spongiibacteraceae			D0	2.71	0.0013	0.019%	0.000%
Bacteria	Proteobacteria	Gammaproteobacte	Cellvibrionales	Spongiibacteraceae	BD1_7clade		D0	2.71	0.0013	0.019%	0.000%
Bacteria	Proteobacteria	Gammaproteobacte	Cellvibrionales	Spongiibacteraceae	BD1_7clade	unknownspec	D0	2.71	0.0013	0.019%	0.000%
Bacteria	Proteobacteria	Gammaproteobacte	Pasteurellales	Pasteurellaceae	Haemophilus		D0	2.78	0.0105	0.024%	0.000%
Bacteria	Proteobacteria	Gammaproteobacte	Pasteurellales	Pasteurellaceae	Haemophilus	Multi_affiliati	D0	2.79	0.0194	0.020%	0.000%
Bacteria	Proteobacteria	Gammaproteobacte	Pseudomonadales	Moraxellaceae	Acinetobacter	unknownspec	D0	2.99	0.0055	0.200%	0.176%
Bacteria	Proteobacteria	Gammaproteobacte	Xanthomonadales	Xanthomonadaceae	Stenotrophomonas	Multi_affiliati	D0	2.96	0.0194	0.086%	0.016%
Bacteria	SHA_109						D0	2.74	0.0105	0.052%	0.001%
Bacteria	SHA_109	unknownclass					D0	2.74	0.0105	0.052%	0.001%
Bacteria	SHA_109	unknownclass	unknownorder				D0	2.74	0.0105	0.052%	0.001%
Bacteria	SHA_109	unknownclass	unknownorder	unknownfamily			D0	2.74	0.0105	0.052%	0.001%
Bacteria	SHA_109	unknownclass	unknownorder	unknownfamily	unknowngenus		D0	2.74	0.0105	0.052%	0.001%
Bacteria	SHA_109	unknownclass	unknownorder	unknownfamily	unknowngenus	unknownspec	D0	2.74	0.0105	0.052%	0.001%
Bacteria							D3	2.62	0.0157	99.890%	99.943%
Bacteria	Actinobacteria	Actinobacteria	Frankiales	Geodermatophilaceae	unknowngenus	Sporichthyasp	D3	3.21	0.0196	0.000%	0.004%
**Bacteria**	**Bacteroidetes**						**D3**	**4.51**	**0.0458**	**13.529%**	**17.269%**
Bacteria	Bacteroidetes	Bacteroidia	Bacteroidales	Marinilabiaceae			D3	3.67	0.0295	0.152%	0.387%
Bacteria	Bacteroidetes	Bacteroidia	Bacteroidales	Marinilabiaceae	Mangroviflexus		D3	3.67	0.0295	0.152%	0.387%
Bacteria	Bacteroidetes	Bacteroidia	Bacteroidales	Marinilabiaceae	Mangroviflexus	unknownspec	D3	3.67	0.0295	0.152%	0.387%
Bacteria	Bacteroidetes	Flavobacteriia	Flavobacteriales	Flavobacteriaceae	Chryseobacterium	Multi_affiliati	D3	3.33	0.0253	0.185%	0.407%
Bacteria	Bacteroidetes	Flavobacteriia	Flavobacteriales	Flavobacteriaceae	Elizabethkingia		D3	3.15	0.0294	0.037%	0.098%
Bacteria	Bacteroidetes	Flavobacteriia	Flavobacteriales	Flavobacteriaceae	Elizabethkingia	Elizabethkingi	D3	3.15	0.0294	0.037%	0.098%
Bacteria	CandidatedivisionOP3						D3	2.71	0.0323	0.001%	0.008%
Bacteria	CandidatedivisionC	unknownclass					D3	2.70	0.0323	0.001%	0.008%
Bacteria	CandidatedivisionC	unknownclass	unknownorder				D3	2.71	0.0323	0.001%	0.008%
Bacteria	CandidatedivisionC	unknownclass	unknownorder	unknownfamily			D3	2.70	0.0323	0.001%	0.008%
Bacteria	CandidatedivisionC	unknownclass	unknownorder	unknownfamily	unknowngenus		D3	2.70	0.0323	0.001%	0.008%
Bacteria	CandidatedivisionC	unknownclass	unknownorder	unknownfamily	unknowngenus	unknownspec	D3	2.72	0.0323	0.001%	0.008%
Bacteria	Cyanobacteria	Cyanobacteria	SubsectionI	FamilyI	Merismopedia		D3	2.78	0.0196	0.000%	0.020%
Bacteria	Cyanobacteria	Cyanobacteria	SubsectionI	FamilyI	Merismopedia	Multi_affiliati	D3	2.77	0.0196	0.000%	0.020%
Bacteria	Elusimicrobia						D3	3.02	0.0373	0.027%	0.084%
Bacteria	Elusimicrobia	Elusimicrobia					D3	3.02	0.0373	0.027%	0.084%
Bacteria	Firmicutes	Clostridia	Clostridiales	Lachnospiraceae	LachnospiraceaeNC2004group		D3	2.91	0.0158	0.002%	0.042%
Bacteria	Firmicutes	Clostridia	Clostridiales	Lachnospiraceae	LachnospiraceaeNC2	unknownspec	D3	2.91	0.0158	0.002%	0.042%
Bacteria	Proteobacteria	Alphaproteobacteri	Sphingomonadales	Sphingomonadaceae	Sphingomonas	Multi_affiliati	D3	2.70	0.0347	0.045%	0.042%
**Bacteria**	**Proteobacteria**	**Betaproteobacteria**					**D3**	**4.27**	**0.0185**	**2.544%**	**4.490%**
**Bacteria**	**Proteobacteria**	**Betaproteobacteria**	**Burkholderiales**				**D3**	**4.22**	**0.0133**	**2.162%**	**3.989%**
**Bacteria**	**Proteobacteria**	**Betaproteobacteria**	**Burkholderiales**	**Comamonadaceae**			**D3**	**4.11**	**0.0157**	**1.491%**	**2.815%**
Bacteria	Proteobacteria	Betaproteobacteria	Burkholderiales	Comamonadaceae	Curvibacter		D3	3.22	0.0457	0.048%	0.127%
Bacteria	Proteobacteria	Betaproteobacteria	Burkholderiales	Comamonadaceae	Curvibacter	Multi_affiliati	D3	3.22	0.0457	0.048%	0.127%
Bacteria	Proteobacteria	Betaproteobacteria	Burkholderiales	Comamonadaceae	Giesbergeria		D3	2.96	0.0177	0.008%	0.052%
Bacteria	Proteobacteria	Betaproteobacteria	Burkholderiales	Comamonadaceae	Giesbergeria	Multi_affiliati	D3	2.96	0.0177	0.008%	0.052%
Bacteria	Proteobacteria	Deltaproteobacteria	Desulfovibrionales	Desulfovibrionaceae	Desulfovibrio	unknownspec	D3	2.48	0.0354	0.032%	0.027%
Bacteria	Proteobacteria	Deltaproteobacteria	Myxococcales	Sandaracinaceae			D3	2.83	0.0158	0.001%	0.023%
Bacteria	Proteobacteria	Deltaproteobacteria	Myxococcales	Sandaracinaceae	Sandaracinus		D3	2.83	0.0158	0.001%	0.023%
Bacteria	Proteobacteria	Deltaproteobacteria	Myxococcales	Sandaracinaceae	Sandaracinus	unknownspec	D3	2.83	0.0158	0.001%	0.023%
Bacteria	Proteobacteria	Epsilonproteobacte	Campylobacterales	Helicobacteraceae			D3	2.80	0.0016	0.005%	0.047%
Bacteria	Proteobacteria	Epsilonproteobacte	Campylobacterales	Helicobacteraceae	Sulfuricurvum		D3	2.73	0.0059	0.000%	0.026%
Bacteria	Proteobacteria	Epsilonproteobacte	Campylobacterales	Helicobacteraceae	Sulfuricurvum	Multi_affiliati	D3	2.73	0.0059	0.000%	0.026%
Bacteria	Proteobacteria	Epsilonproteobacte	Campylobacterales	Helicobacteraceae	unknowngenus		D3	2.93	0.0196	0.005%	0.021%
Bacteria	Proteobacteria	Epsilonproteobacte	Campylobacterales	Helicobacteraceae	unknowngenus	unknownspec	D3	2.92	0.0196	0.005%	0.021%
Bacteria	Proteobacteria	Gammaproteobacte	Legionellales	Legionellaceae	Legionella	Multi_affiliati	D3	2.70	0.0209	0.001%	0.018%
Bacteria	Proteobacteria	Multi_affiliation					D3	3.16	0.0143	0.029%	0.104%
Bacteria	Proteobacteria	Multi_affiliation	Multi_affiliation				D3	3.16	0.0143	0.029%	0.104%
Bacteria	Proteobacteria	Multi_affiliation	Multi_affiliation	Multi_affiliation			D3	3.16	0.0143	0.029%	0.104%
Bacteria	Proteobacteria	Multi_affiliation	Multi_affiliation	Multi_affiliation	Multi_affiliation		D3	3.16	0.0143	0.029%	0.104%
Bacteria	Proteobacteria	Multi_affiliation	Multi_affiliation	Multi_affiliation	Multi_affiliation	Multi_affiliati	D3	3.16	0.0143	0.029%	0.104%
Bacteria	Proteobacteria	TA18					D3	2.82	0.0406	0.043%	0.090%
Bacteria	Proteobacteria	TA18	unknownorder				D3	2.82	0.0406	0.043%	0.090%
Bacteria	Proteobacteria	TA18	unknownorder	unknownfamily			D3	2.82	0.0406	0.043%	0.090%

Bacteria	Proteobacteria	TA18	unknownorder	unknownfamily	unknowngenus		D3	2.82	0.0406	0.043%	0.090%
Bacteria	Proteobacteria	TA18	unknownorder	unknownfamily	unknowngenus	unknownspec	D3	2.82	0.0406	0.043%	0.090%
Bacteria	Spirochaetae	Spirochaetes	Spirochaetales	Spirochaetaceae	Treponema2		D3	2.82	0.0209	0.002%	0.010%
Bacteria	Spirochaetae	Spirochaetes	Spirochaetales	Spirochaetaceae	Treponema2	unknownspec	D3	2.82	0.0209	0.002%	0.010%
Bacteria	Spirochaetae	Spirochaetes	Spirochaetales	Spirochaetaceae	unknowngenus		D3	3.24	0.0252	0.012%	0.067%
Bacteria	Spirochaetae	Spirochaetes	Spirochaetales	Spirochaetaceae	unknowngenus	unknownspec	D3	3.23	0.0252	0.012%	0.067%
Bacteria	Verrucomicrobia	Verrucomicrobiae					D3	3.27	0.0426	0.227%	0.380%
Bacteria	Verrucomicrobia	Verrucomicrobiae	Verrucomicrobiales				D3	3.27	0.0426	0.227%	0.380%
Bacteria	Verrucomicrobia	Verrucomicrobiae	Verrucomicrobiales	Verrucomicrobiaceae			D3	3.27	0.0426	0.227%	0.380%
Bacteria	Verrucomicrobia	Verrucomicrobiae	Verrucomicrobiales	Verrucomicrobiaceae	Haloferula		D3	3.12	0.0247	0.029%	0.087%
Bacteria	Verrucomicrobia	Verrucomicrobiae	Verrucomicrobiales	Verrucomicrobiaceae	Haloferula	unknownspec	D3	3.12	0.0247	0.029%	0.087%
Archaea							D0	2.62	0.0157	0.110%	0.057%
Archaea	Euryarchaeota						D0	2.62	0.0157	0.110%	0.057%
Archaea	Euryarchaeota	Methanobacteria					D0	2.62	0.0157	0.110%	0.057%
Archaea	Euryarchaeota	Methanobacteria	Methanobacteriales				D0	2.62	0.0157	0.110%	0.057%
Archaea	Euryarchaeota	Methanobacteria	Methanobacteriales	Methanobacteriaceae			D0	2.62	0.0157	0.110%	0.057%
Archaea	Euryarchaeota	Methanobacteria	Methanobacteriales	Methanobacteriaceae	Methanobrevibacter		D0	2.62	0.0157	0.110%	0.057%
Archaea	Euryarchaeota	Methanobacteria	Methanobacteriales	Methanobacteriaceae	Methanobrevibacter	Multi_affiliati	D0	2.82	0.0232	0.089%	0.029%
